# Cyclic Amide-Linked Oxazolidinone Triazoles as Inhibitors of the T-Box Riboswitch

**DOI:** 10.3390/molecules31010029

**Published:** 2025-12-22

**Authors:** Eric Parsons, Ali H. Aldhumani, Emily A. Fairchild, Oluwaseun B. Adegbite, Jessica M. Roberts, Jennifer V. Hines, Stephen C. Bergmeier

**Affiliations:** Department of Chemistry & Biochemistry, Ohio University, Athens, OH 45701, USA; parsonse@ohio.edu (E.P.); aa948714@ohio.edu (A.H.A.); ef685320@ohio.edu (E.A.F.); oa080221@ohio.edu (O.B.A.); jroberts@ohio.edu (J.M.R.); hinesj@ohio.edu (J.V.H.)

**Keywords:** RNA, oxazolidinone, triazole, docking

## Abstract

Antimicrobial resistance remains a critical global health challenge, and was intensified by the COVID-19 pandemic. To address this growing threat, novel antibacterial agents targeting unconventional mechanisms are urgently needed. One promising strategy involves inhibiting bacterial riboswitches—RNA elements that regulate gene expression. Unlike most riboswitches that respond to small-molecule metabolites, the T-box riboswitch uniquely binds non-aminoacylated tRNA and is predominantly found in Gram-positive bacteria, making it an attractive target due to its conserved sequences and regulatory role over essential genes. This study explored oxazolidinone- and triazole-based compounds as potential inhibitors of the T-box riboswitch. Prior investigations into tricyclic oxazolidinones revealed an allosteric modulator that effectively inhibited T-box riboswitch transcriptional readthrough in vitro, though it showed limited disruption of the isolated tRNA–antiterminator complex. To enhance RNA-binding affinity and stereoselectivity, a macrocyclic oxazolidinone scaffold was designed, incorporating a strategic substituent to expand the interaction footprint. A synthetically viable candidate was identified, and computational docking studies suggested that one of the designed compounds may interfere with tRNA-induced transcription by forming π–π stacking interactions with G5 in the antiterminator region. These findings support the potential of targeting the T-box riboswitch with structurally optimized small molecules as a novel antibacterial strategy.

## 1. Introduction

Antimicrobial resistance to current treatments continues to be a significant global health threat that was further exacerbated by the COVID pandemic [1,2]. The Centers for Disease Control and Prevention’s list of resistant threats include bacteria and fungi, with one-third (7/20) being Gram-positive bacteria [3]. To overcome this serious threat to human health, completely novel antibacterial agents are needed. Developing inhibitors that target the RNA of bacterial riboswitches is a novel area of antibacterial drug discovery [4,5,6]. Riboswitches are located in the 5′ untranslated region of mRNA and regulate gene expression at the level of transcription or translation by structurally responding to effector molecule binding or environmental conditions [7,8,9,10]. Most riboswitches respond to a small-molecule metabolite as their effector molecule, but the T-box riboswitch specifically binds and structurally responds to cognate non-aminoacylated tRNA. Found primarily in Gram-positive bacteria, the T-box riboswitch is an ideal target for antibacterial drug discovery due to regions of high sequence conservation and the many essential genes regulated [5,11,12].

Previous studies by our group have identified novel oxazolidinone ligands that bind the T-box riboswitch antiterminator element [13] and modulate tRNA-induced transcription readthrough [14,15]. T-box riboswitch inhibition by a small molecule has also been implicated in antibacterial activity specific to Gram-positive bacteria [16,17].

**Figure 1:**
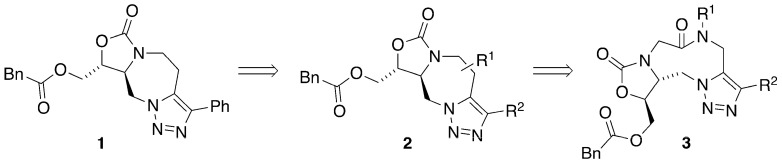
Evolution of current version of oxazolidinones.

We have examined a number of oxazolidinone- and triazole-based compounds in order to understand how small molecules can interact with and inhibit the T-box riboswitch [14,15,18]. Most recently, we reported on the design, synthesis, and evaluation of a preliminary set of tricyclic oxazolidinones (Figure 1) [19]. Overall, the RNA-binding results for compound **1** suggest that these ligands act as allosteric modulators of T-box riboswitch function. While these ligands effectively inhibited transcriptional readthrough in the T-box riboswitch functional assay, there was modest to no inhibition in the formation of the tRNA–antiterminator complex observed in a fluorescence anisotropy assay and minimal stereoselectivity. Thus, rather than disrupting the complex formation via competitive inhibition, these ligands may bind to an allosteric site (a site other than where tRNA forms contacts with the antiterminator) to then cause a conformational change, which results in the observed disruption of the function of the T-box antiterminator [19].

Consequently, we set out to design an oxazolidinone-containing macrocycle that allowed for a larger RNA-binding footprint (to enhance stereoselective interactions) while retaining conformational restriction. We planned to introduce an additional substituent (e.g., R^1^) on the chain linking the triazole to the oxazolidinone, as shown in compound **2**. Compound **3** appeared to be a synthetically tractable version of the proposed compound **2**. Lactam **3** would allow for the introduction of a variety of substituents in the R^1^ position through substitution of the amide.

## 2. Results

For our initial studies, we planned a small selection of substituents for positions R^1^ and R^2^. Our previous studies [15,20], as well as other RNA/ligand studies [21], have indicated the importance of hydrophobic interactions and π–π stacking for conferring specificity, as opposed to electrostatic (ionic) interactions, which are much less specific. Consequently we looked at those analogs as well as H-bonding substituents.

**Scheme 1:**
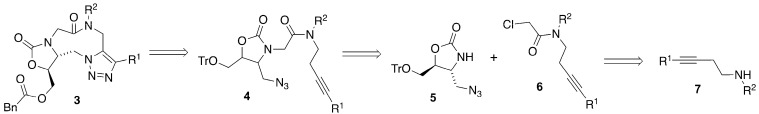
Retrosynthesis of cyclic amide-linked oxazolidinone triazoles.

The synthesis of triazole **3** is outlined in Scheme 1. Triazole **3** should be readily available through an intramolecular dipolar cycloaddition of azido alkyne **4**. An alkylation of the known azido-oxazolidinone **5** with α-chloroamide **6** would provide azidoalkyne **4**. The α-chloroamide **6** can be readily prepared by acylation of amine **7**. This amine will provide a ready scaffold upon which to introduce both substituents R^1^ and R^2^.

**Scheme 2:**
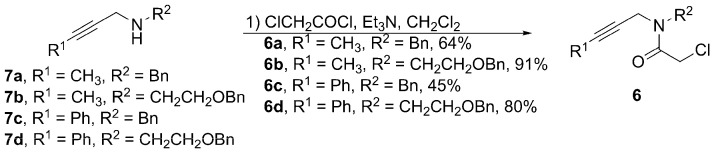
Synthesis of α-chloroamides.

As shown in Scheme 2, treatment of known (**7a** [22], **7c** [23]) or commercially available (**7b**, **7d**) amines with chloroacetyl chloride provided generally good yields of α-chloroamides **6a**–**6d**.

**Scheme 3:**
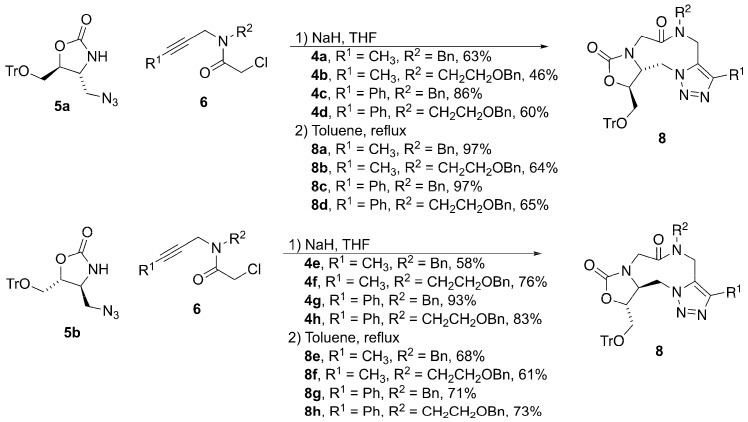
Synthesis of triazoles.

Once the four required α-chloroamides **6a**–**6d** were obtained, they were used for the alkylation of enantiopure oxazolidinone azides **5a** and **5b** (Scheme 3) [15]. Treatment of oxazolidinone **5** with NaH followed by the α-chloroamide provided intermediate alkynyl azides **4a**–**4h**. These enantiopure azidoalkynes were then subjected to a thermal cycloaddition reaction to generate triazoles **8a**–**8h**.

**Scheme 4:**
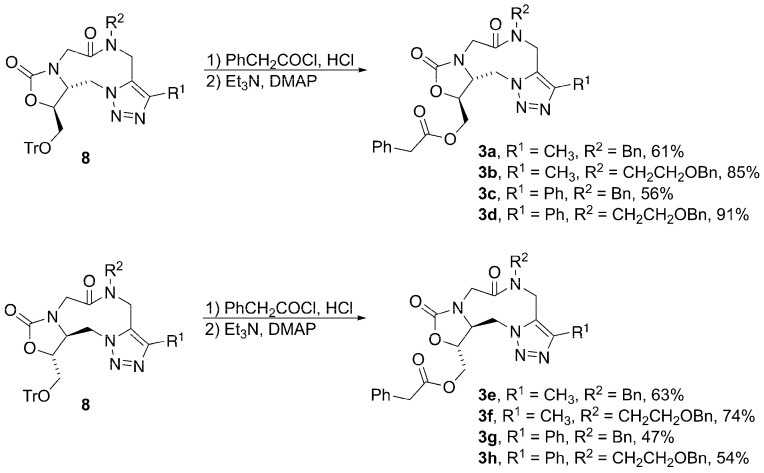
Acylation of cyclic triazoles.

The trityl deprotection–acylation reaction follows a modified version of a one-pot procedure previously reported [24]. The original procedure uses non-distilled acid chloride to both deprotect and acylate a trityl-protected alcohol, as outlined in Scheme 4. This procedure, while effective, produced a byproduct that was difficult to remove. In order to avoid the formation of this byproduct, the phenylacetyl chloride was first distilled and a catalytic amount of anhydrous HCl added. In order to drive the reaction to completion, DMAP and Et_3_N were added to the reaction once it was shown by TLC that all of the trityl-protected triazole starting material had been consumed. This second step ensured that all of the primary alcohol that resulted from trityl group removal was acylated to form the desired ester product. This provided enantiopure final compounds **3a**, **3c**, **3e**, and **3g**.

**Scheme 5:**
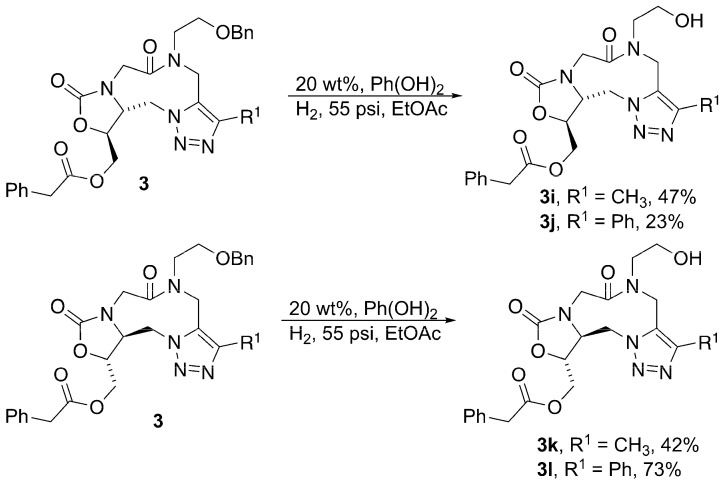
Deprotection.

The final step that was required in the synthesis of the desired enantiopure analogs was the reductive debenzylation of benzyl ethers **3b**, **3d**, **3f**, and **3h** to provide the primary alcohol (Scheme 5). Various reaction conditions were attempted, and eventually palladium hydroxide was determined to be the best choice as the catalyst to remove the benzyl group. With the purification and isolation of these last four analogs (**3i**–**3l**), the synthesis of the eight desired enantiopure analogs was complete.

**Figure 2:**
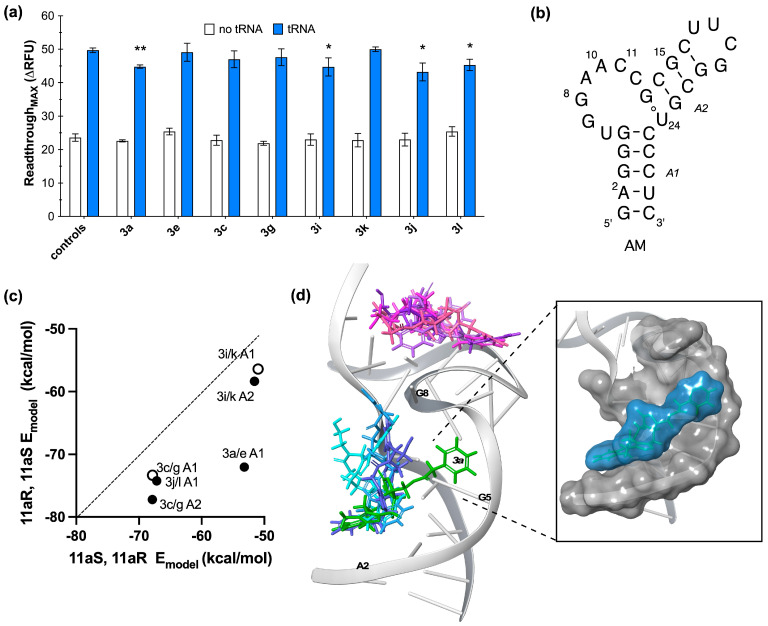
T-box riboswitch inhibition and enantiospecific ligand docking to antiterminator model RNA. (**a**) GlyQS T-box riboswitch transcription readthrough with (filled bar) and without (clear bar) tRNA (n = 3, stars indicate *p*-value significance ** *p* = 0.0015, * 0.034 > *p* > 0.014) in the absence (controls) or presence of ligands (100 µm). (**b**) Antiterminator model RNA AM (helices A1 and A2 as indicated), (**c**) Ligand enantiomeric pair comparison of Glide docking Emodel values with general helix docking location indicated (A1 or A2) for the lowest Emodel docking pose (filled circle). Dashed line represents the y = x identity line. For ligands that bound the A2 helix, the lowest Emodel for a docking pose in the A1 helix is also plotted (open circles). (**d**) Ligand docking poses with lowest Glide Emodel value, **3a** shown in green (inset of **3a** binding site surface).

To investigate the effect of the ligands on T-box riboswitch function, we used a fluorescence-monitored transcription of the gly*QS* riboswitch assay previously developed by our lab [25]. Four of the compounds (**3a**, **3i**, **3j**, and **3l**) inhibited tRNA-induced gly*QS* transcription readthrough (Figure 2a). None of the ligands inhibited the basal level of transcription readthrough (*p* value > 0.5 in the absence of tRNA). For **3a**, **3i**, **3j**, and **3l**, this lack of basal level inhibition also indicates that the observed inhibition of tRNA-induced transcription readthrough is likely specific to the T-box riboswitch mechanism. Compound **3a** showed the most significant inhibition of tRNA-induced transcription readthrough (14% inhibition, *p* value = 0.0015). The remaining compounds (**3c**, **3e**, **3g**, **3k**) had no effect on riboswitch function under the conditions assayed (*p* value > 0.5 with and without tRNA).

Computational docking studies were conducted using Glide (Schrödinger) and our previously optimized protocol [15] to investigate possible ligand–antiterminator interactions. Previous studies with this protocol have demonstrated that the computationally derived E_model_ value for the most stable docking pose can correlate well with experimental binding data [19]. All of the ligands in this study were docked to the NMR-derived structure of antiterminator model RNA, AM [26], and poses selected based on lowest E_model_ value or docking location as indicated (Figure 2b–d).

Notably, **3a** was the only ligand that docked entirely in the *A1* helix. In the docking pose with the lowest E_model_ value, **3a** aligned along the major groove of the *A1* helix forming favorable van der Waals contacts with residues A2 to G4 and π–π stacking with the 3′ face of G5. Three of the compounds (**3e**, **3j**, and **3l**) docked partially in the *A1* helix accompanied by significant interactions with the bulge nucleotides. The four remaining compounds had the lowest energy docked pose in the *A2* helix (**3c**, **3g**, **3k**). Regarding stereospecific interactions, of the enantiomeric pairs, **3a** compared to it’s enantiomer **3e** had the greatest difference in E_model_ values (26%) and docking location (Figure 2c,d). Notably, **3a** was the only ligand that docked entirely in the A1 helix.

For **3a**, the combination of *A1* helix docking location and riboswitch inhibition was consistent with ligand binding interfering with the antiterminator function. Previous studies in our lab identified that ligands that bind along the antiterminator *A1* helix were antagonists, while ligands that docked only the bulge nucleotides in the antiterminator (U6-C12 of AM, Figure 2b) were agonists of T-box riboswitch function [15]. Given the potential significance of docking in the *A1* helix, we also examined alternate docking poses for ligands **3c**, **3g**, **3i**, and **3k** other than their most stable *A2* helix pose. While they all had a docking pose in the *A1* helix with weaker E_model_ values (Figure 2c), each ligand’s pose also had significant contacts with the bulge nucleotides in addition to the *A1* helix (Supplementary Material) and did not align along the groove of the *A1* helix in the way that **3a** did.

The consequences of a ligand binding along the *A1* helix of the antiterminator can be interpreted in light of recent structural studies of the T-box riboswitch. In the cryo-EM structure of the gly*QS* discriminator region complexed with tRNA, the gly*QS* nucleotide U155 (equivalent to U6 in AM, Figure 2) forms a base pair with the 3′ terminal nucleotide of tRNA (A75) and is stacked on the 3′ face of gly*QS* G154 (G5 of AM) and subsequently stabilized by other long-range interactions [27]. Consequently, it is possible that **3a** forming π–π stacking contacts with G5 in the antiterminator may disrupt formation of a tRNA–antiterminator conformer, which is required for full functioning of tRNA-induced transcription readthrough. The conformer of the tRNA–antiterminator complex needed for the subsequent stabilization could be sensitive to allosteric inhibition by a ligand binding along the *A1* helix. This would be consistent with our previous identification of T-box riboswitch antagonists binding along the *A1* helix [15] and identification of other likely allosteric inhibitors [19].

## 3. Materials and Methods

### 3.1. General Experimental

CH_2_Cl_2_ and THF were dried using a SOLV-TEK solvent purification system. All other dry solvents (acetone, DMF, toluene, Et_3_N, EtOAc) were purified by drying overnight with molecular sieves (4 Å) and distilling immediately before use. Unless otherwise noted, all reactions were conducted under an inert atmosphere (N_2_ or Argon). A DigiMelt MPA 160 melting point apparatus (SRS, Sunnyvale, CA, USA) was used to determine melting points, and all melting points are reported uncorrected. An AUTOPOL ^®^ IV (Rudolph Research Analytical, Hackettstown, NJ, USA) polarimeter with a sodium (λ = 589 nm) lamp was used to measure specific rotations, which are reported as follows: [α]_λ_ ^T °C^ (*c* = g/100 mL, solvent). HPLC analysis for all tested compounds was performed using a Shimadzu LC-10AT instrument (Kyoto, Japan) equipped with a UV detector employing a Hypersil Gold C8 HPLC column (Waltham, MA, USA, 150 × 4.6 mm × 5 μm), with a flow rate of 1 mL/min (HPLC solvent systems described in General HPLC methods A and B). Tested compounds showed >95% purity unless otherwise stated. ^1^H NMR and ^13^C NMR spectra were recorded with either a Bruker Ascend 500 MHz spectrometer (Billerica, MA, USA) or a Bruker Avance 300 MHz spectrometer. Chemical shifts are reported in ppm on the δ scale relative to deuterated chloroform as an internal standard. NMR data are reported as follows: chemical shift, multiplicity (s = singlet, d = doublet, t = triplet, q = quartet, m = multiplet), coupling constant in Hz, integration. The capillary HRMS analyses were performed using a Thermo Scientific Q Exactive Plus Orbitrap mass spectrometer (Waltham, MA, USA). HPLC was performed using a 150 × 4.6 mm Hypersil GOLD C8 HPLC Column; Method A, 30% CH_3_CN in H_2_O (containing 1 mM HCO_2_H) going to 90% CH_3_CN over 14 min; Method B, 50% CH_3_CN in H_2_O (containing 1 mM HCO_2_H) going to 90% CH_3_CN over 14 min.

### 3.2. General Procedure A: Deprotection and Acylation Reaction

Triazole **9** was dissolved in dry CH_2_Cl_2_ (0.2 M) and cooled to 0 °C. Distilled phenylacetyl chloride (500 mol %) was added, followed by addition of 1.5 M anhydrous HCl in EtOAc (50 mol %). The reaction was stirred at 0 °C for 5 min, and was then warmed to room temperature. After approximately 18 h, at which point the trityl-protected triazole starting material was shown to be consumed by TLC, the reaction was cooled to 0 °C and DMAP (100 mol %) was added, followed by the addition of Et_3_N (700 mol %). The reaction was stirred for an additional 2 h, then methanol (4500 mol %) was added and stirred for 30 min. The reaction was concentrated and the resulting crude material was dissolved in EtOAc and washed with saturated aqueous NaHCO_3_ (2×) and water (2×). The combined aqueous phases were extracted twice with EtOAc. The combined organic phases were then washed with brine, dried over MgSO_4_, filtered, concentrated, and chromatographed.

*((11S,11aR)-5-benzyl-3-methyl-6,9-dioxo-4,5,6,7,9,11,11a,12-octahydrooxazolo[3,4-a][1,2,3]triazolo[1,5-d][1,4,7]triazonin-11-yl)methyl 2-phenylacetate* (**3a**). Synthesized from triazole **9a** (120 mg, 0.20 mmol) using general procedure A. The crude product was purified by column chromatography (55% EtOAc in hexanes) to afford 58 mg (61%) of **3a** as a white solid, mp 115.0–116.0 °C; [α]_D_^25^ −73.714 (c = 0.175, CHCl_3_). ^1^H NMR (500 MHz, CDCl_3_) δ 7.42–7.24 (m, 8H), 7.15–7.09 (m, 2H), 5.00 (d, *J* = 14.2 Hz, 1H), 4.83 (d, *J* = 16.6 Hz, 1H), 4.67 (d, *J* = 17.7 Hz, 1H), 4.48 (d, *J* = 15.6 Hz, 1H), 4.38 (q, *J* = 3.8 Hz, 1H), 4.27 (d, *J* = 3.5 Hz, 2H), 4.25–4.21 (m, 1H), 4.22–4.17 (m, 1H), 3.87–3.78 (m, 2H), 3.67 (d, *J* = 6.4 Hz, 3H), 2.17 (s, 3H). ^13^C NMR (125 MHz, CDCl_3_) δ 170.9, 168.1, 155.6, 142.9, 134.6, 133.3, 129.5, 129.4, 129.1, 128.9, 128.7, 128.1, 127.7, 72.8, 63.4, 57.7, 50.2, 49.0, 48.9, 41.4, 40.9, 10.3. HRMS: calculated for C_26_H_27_N_5_O_5_·H^+^, 490.2085; found, 490.2102. HPLC (254 nm, method B) 3.00 min, 98%.

*((11S,11aR)-5-(2-(benzyloxy)ethyl)-3-methyl-6,9-dioxo-4,5,6,7,9,11,11a,12-octahydrooxazolo[3,4-a][1,2,3]triazolo[1,5-d][1,4,7]triazonin-11-yl)methyl 2-phenylacetate* (**3b**). Synthesized from triazole **9b** (0.27 g, 0.41 mmol) using general procedure A. The crude product was purified by column chromatography (70% EtOAc in hexanes) to afford 180 mg (85%) of **3b** as a white solid, mp 114.0–116.0 °C; [α]_D_^25^ −76.3 (*c* 0.35, CHCl_3_); ^1^H NMR (500 MHz, CDCl_3_) δ 7.41–7.21 (m, 10H), 4.80 (d, *J* = 7.8 Hz, 1H), 4.77 (d, *J* = 8.8 Hz, 1H), 4.65 (d, *J* = 17.7 Hz, 1H), 4.48–4.35 (m, 3H), 4.35 (d, *J* = 3.6 Hz, 2H), 4.27 (d, *J* = 3.4 Hz, 2H), 3.78–3.74 (m, 1H), 3.66 (s, 2H), 3.65–3.57 (m, 3H), 3.42 (d, *J* = 16.4 Hz, 1H), 3.33–3.24 (m, 1H), 2.21 (s, 3H); ^13^C NMR (125 MHz, CDCl_3_) δ 170.8, 168.4, 155.6, 142.7, 137.5, 133.3, 129.3, 129.1, 128.6, 128.1, 127.8, 127.7, 73.4, 72.8, 68.3, 63.4, 57.7, 57.6, 49.0, 48.9, 43.3, 41.3, 10.2. HRMS: calculated for C_28_H_31_N_5_O_6_·Na^+^, 556.21665; found, 556.21820. HPLC (254 nm, method B) 3.31 min, 100%.

*((11S,11aR)-5-benzyl-6,9-dioxo-3-phenyl-4,5,6,7,9,11,11a,12-octahydrooxazolo[3,4-a][1,2,3]triazolo[1,5-d][1,4,7]triazonin-11-yl)methyl 2-phenylacetate* (**3c**). Synthesized from triazole **9c** (0.12 g, 0.18 mmol) using general procedure A. The crude product was purified by column chromatography (45% EtOAc in hexanes) to afford 0.056 g (56%) of **3c** as a white solid, mp 122.0–124.0 °C; [α]_D_^25^ −65.4 (*c* 0.19, CHCl_3_); ^1^H NMR (500 MHz, CDCl_3_) δ 7.46–7.10 (m, 14H), 6.92 (d, *J* = 7.4 Hz, 2H), 4.86 (d, *J* = 16.7 Hz, 1H), 4.70 (d, *J* = 14.3 Hz, 1H), 4.53–4.45 (m, 2H), 4.41 (q, *J* = 3.9 Hz, 1H), 4.29–4.25 (m, 2H), 4.23 (dd, *J* = 15.6, 2.7 Hz, 1H), 4.06 (d, *J* = 14.2 Hz, 1H), 3.87–3.82 (m, 1H), 3.67 (s, 2H), 3.53 (d, *J* = 16.7 Hz, 1H); ^13^C NMR (125 MHz, CDCl_3_) δ 170.8, 167.9, 155.4, 147.2, 134.1, 133.2, 129.5, 129.3, 129.0, 128.9, 128.8, 128.4, 128.3, 128.1, 127.6, 72.7, 63.4, 57.5, 50.6, 49.2, 48.8, 42.2, 41.2. HRMS: calculated for C_31_H_29_N_5_O_5_·H^+^, 552.2242; found, 552.2268; HPLC (254 nm, method B) 4.4 min, 99%.

*((11S,11aR)-5-(2-(benzyloxy)ethyl)-6,9-dioxo-3-phenyl-4,5,6,7,9,11,11a,12-octahydrooxazolo[3,4-a][1,2,3]triazolo[1,5-d][1,4,7]triazonin-11-yl)methyl 2-phenylacetate* (**3d**). Synthesized from triazole **9d** (0.29 g, 0.40 mmol) using general procedure A. The crude product was purified by column chromatography (57.5% EtOAc in hexanes) to afford 0.22 g (91%) of **3d** as a white solid, mp 127.0–128.3 °C; [α]_D_^25^ −48.219 (*c* 0.36, CHCl_3_); ^1^H NMR (500 MHz, CDCl_3_) δ 7.55–7.49 (m, 2H), 7.44–7.27 (m, 11H), 7.14–7.09 (m, 2H), 5.03 (d, *J* = 17.9 Hz, 1H), 4.85 (t, *J* = 17.3 Hz, 2H), 4.54 (s, 2H), 4.46 (q, *J* = 3.9 Hz, 1H), 4.29 (d, *J* = 3.5 Hz, 2H), 4.24 (s, 2H), 3.85–3.81 (m, 1H), 3.68 (s, 2H), 3.56–3.48 (m, 3H), 3.45 (d, *J* = 16.9 Hz, 1H), 3.43–3.36 (m, 1H); ^13^C NMR (125 MHz, CDCl_3_) δ 170.9, 168.6, 155.6, 147.0, 137.4, 133.3, 129.8, 129.4, 129.1, 129.1, 128.9, 128.8, 128.6, 128.2, 127.9, 127.7, 73.2, 72.9, 67.9, 63.6, 57.6, 49.4, 48.9, 47.6, 44.3, 41.3. HRMS: calculated for C_33_H_33_N_5_O_6_·H^+^, 596.2504; found, 596.2526; HPLC (254 nm, method B) 5.1 min, 100%.

*((11R,11aS)-5-benzyl-3-methyl-6,9-dioxo-4,5,6,7,9,11,11a,12-octahydrooxazolo[3,4-a][1,2,3]triazolo[1,5-d][1,4,7]triazonin-11-yl)methyl 2-phenylacetate* (**3e**). Synthesized from triazole **9e** (120 mg, 0.20 mmol) using general procedure A. The crude product was purified by column chromatography (55% EtOAc in hexanes) to afford 61 mg (63%) of **3e** as a white solid; mp 115.0–116.0 °C; [α]_D_^25^ +73.143 (*c* 0.18, CHCl_3_); ^1^H NMR (500 MHz, CDCl_3_) δ 7.42–7.24 (m, 8H), 7.15–7.09 (m, 2H), 5.00 (d, *J* = 14.2 Hz, 1H), 4.83 (d, *J* = 16.6 Hz, 1H), 4.67 (d, *J* = 17.7 Hz, 1H), 4.48 (d, *J* = 15.6 Hz, 1H), 4.38 (q, *J* = 3.8 Hz, 1H), 4.27 (d, *J* = 3.5 Hz, 2H), 4.25–4.21 (m, 1H), 4.22–4.17 (m, 1H), 3.87–3.78 (m, 2H), 3.67 (d, *J* = 6.4 Hz, 3H), 2.17 (s, 3H); ^13^C NMR (125 MHz, CDCl_3_) δ 170.9, 168.1, 155.6, 142.9, 134.6, 133.3, 129.5, 129.4, 129.1, 128.9, 128.7, 128.1, 127.7, 72.8, 63.4, 57.7, 50.2, 49.0, 48.9, 41.4, 40.9, 10.3. HRMS: calculated for C_26_H_27_N_5_O_5_·H^+^, 490.2085; found, 490.2102; HPLC (254 nm, method B) 3.0 min, 98%.

*((11R,11aS)-5-(2-(benzyloxy)ethyl)-3-methyl-6,9-dioxo-4,5,6,7,9,11,11a,12-octahydrooxazolo[3,4-a][1,2,3]triazolo[1,5-d][1,4,7]triazonin-11-yl)methyl 2-phenylacetate* (**3f**). Synthesized from triazole **9f** (0.26 g, 0.40 mmol) using general procedure A. The crude product was purified by column chromatography (70% EtOAc in hexanes) to afford 0.16 g (74%) of **3f** as a white solid, mp 114.0–116.0 °C; [α]_D_^25^ +75.775 (*c* 0.355, CHCl_3_); ^1^H NMR (500 MHz, CDCl_3_) δ 7.41–7.21 (m, 10H), 4.80 (d, *J* = 7.8 Hz, 1H), 4.77 (d, *J* = 8.8 Hz, 1H), 4.65 (d, *J* = 17.7 Hz, 1H), 4.48–4.35 (m, 3H), 4.35 (d, *J* = 3.6 Hz, 2H), 4.27 (d, *J* = 3.4 Hz, 2H), 3.78–3.74 (m, 1H), 3.66 (s, 2H), 3.65–3.57 (m, 3H), 3.42 (d, *J* = 16.4 Hz, 1H), 3.33–3.24 (m, 1H), 2.21 (s, 3H). ^13^C NMR (125 MHz, CDCl_3_) δ 170.8, 168.4, 155.6, 142.7, 137.5, 133.3, 129.3, 129.1, 128.6, 128.1, 127.8, 127.7, 73.4, 72.8, 68.3, 63.4, 57.7, 57.6, 49.0, 48.9, 43.3, 41.3, 10.2. HRMS: calculated for C_28_H_31_N_5_O_6_·Na^+^, 556.2167; found, 556.2182. HPLC (254 nm, method B) 3.3 min, 100%.

*((11R,11aS)-5-benzyl-6,9-dioxo-3-phenyl-4,5,6,7,9,11,11a,12-octahydrooxazolo[3,4-a][1,2,3]triazolo[1,5-d][1,4,7]triazonin-11-yl)methyl 2-phenylacetate* (**3g**)**.** Synthesized from triazole **9g** (0.10 g, 0.15 mmol) using general procedure A. The crude product was purified by column chromatography (40% EtOAc in toluene) to afford 0.039 g (47%) of **3g** as a white solid, mp 122.0–124.0 °C; [α]_D_^25^ +64.571 (*c* 0.175, CHCl_3_); ^1^H NMR (500 MHz, CDCl_3_) δ 7.46–7.10 (m, 14H), 6.92 (d, *J* = 7.4 Hz, 2H), 4.86 (d, *J* = 16.7 Hz, 1H), 4.70 (d, *J* = 14.3 Hz, 1H), 4.53–4.45 (m, 2H), 4.41 (q, *J* = 3.9 Hz, 1H), 4.29–4.25 (m, 2H), 4.23 (dd, *J* = 15.6, 2.7 Hz, 1H), 4.06 (d, *J* = 14.2 Hz, 1H), 3.87–3.82 (m, 1H), 3.67 (s, 2H), 3.53 (d, *J* = 16.7 Hz, 1H). ^13^C NMR (125 MHz, CDCl_3_) δ 170.8, 167.9, 155.4, 147.2, 134.1, 133.2, 129.5, 129.3, 129.0, 128.9, 128.8, 128.4, 128.3, 128.2, 127.6, 72.7, 63.4, 57.5, 50.6, 49.2, 48.8, 42.2, 41.2. HRMS: calculated for C_31_H_29_N_5_O_5_·H^+^, 552.2242; found, 552.2269; HPLC (254 nm, method B) 4.4 min, 99%.

*((11R,11aS)-5-(2-(benzyloxy)ethyl)-6,9-dioxo-3-phenyl-4,5,6,7,9,11,11a,12-octahydrooxazolo[3,4-a][1,2,3]triazolo[1,5-d][1,4,7]triazonin-11-yl)methyl 2-phenylacetate* (**3h**). Synthesized from triazole **9h** (0.25 g, 0.35 mmol) using general procedure A. The crude product was purified by column chromatography (52.5% EtOAc in hexanes) to afford 0.11 g (54%) of **3h** as a white solid, mp 127.0–128.3 °C; [α]_D_^25^ +48.1 (*c* 0.36, CHCl_3_); ^1^H NMR (500 MHz, CDCl_3_) δ 7.55–7.49 (m, 2H), 7.44–7.27 (m, 11H), 7.14–7.09 (m, 2H), 5.03 (d, *J* = 17.9 Hz, 1H), 4.85 (t, *J* = 17.3 Hz, 2H), 4.54 (s, 2H), 4.46 (q, *J* = 3.9 Hz, 1H), 4.29 (d, *J* = 3.5 Hz, 2H), 4.24 (s, 2H), 3.85–3.81 (m, 1H), 3.68 (s, 2H), 3.56–3.48 (m, 3H), 3.45 (d, *J* = 16.9 Hz, 1H), 3.43–3.36 (m, 1H); ^13^C NMR (125 MHz, CDCl_3_) δ 170.9, 168.6, 155.6, 147.0, 137.4, 133.3, 129.8, 129.4, 129.1, 129.0, 128.9, 128.8, 128.6, 128.2, 127.9, 127.7, 73.2, 72.9, 67.9, 63.6, 57.60, 49.4, 48.9, 47.6, 44.3, 41.3. HRMS: calculated for C_33_H_33_N_5_O_6_·H^+^, 596.2504; found, 596.2526. HPLC (254 nm, method B) 5.1 min, 100%.

### 3.3. General Procedure B: Debenzylation

To a 50 mL PARR reaction vessel was added 20 wt. % Pd(OH)_2_ on carbon (200 weight % added). EtOAc (2 mL) was then added to wet the catalyst. The requisite ester (100 weight %) was dissolved in EtOAc (4 mL) and then added to the PARR reaction flask, which was then sealed and filled with H_2_ at 55 psi. The PARR reaction vessel was evacuated with an aspirator and filled with H_2_ at 55 psi three times and was then left to shake/react overnight. After ~24 h (or until the reaction was shown to be complete), the flask was evacuated and the reaction mixture was filtered through a fritted glass funnel layered with sand, celite, and filter paper (in that order). The filtrate was then concentrated in vacuo and the crude product purified by column chromatography to afford the pure debenzylated product.

*((11S,11aR)-5-(2-hydroxyethyl)-3-methyl-6,9-dioxo-4,5,6,7,9,11,11a,12-octahydrooxazolo[3,4-a][1,2,3]triazolo[1,5-d][1,4,7]triazonin-11-yl)methyl 2-phenylacetate* (**3i**). Synthesized from ester **3b** (0.075 g, 0.14 mmol) using general procedure B. The crude product was purified by column chromatography (3% iPrOH in EtOAc) to afford 0.029 g (47%) of **3i** as a white solid, mp 126.2–128.0 °C; [α]_D_^25^ −97.7 (*c* 0.13, CHCl_3_); ^1^H NMR (500 MHz, CDCl_3_) δ 7.41–7.23 (m, 5H), 4.89–4.80 (m, 2H), 4.60–4.45 (m, 3H), 4.45–4.40 (m, 1H), 4.29 (d, *J* = 3.5 Hz, 2H), 3.84–3.77 (m, 2H), 3.77–3.70 (m, 1H), 3.67 (s, 2H), 3.59 (dd, *J* = 14.2, 4.2 Hz, 1H), 3.44 (d, *J* = 16.2 Hz, 1H), 3.26–3.17 (m, 1H), 2.28 (s, 3H); ^13^C NMR (125 MHz, CDCl_3_) δ 170.9, 169.4, 155.7, 142.9, 133.3, 129.4, 129.1, 128.4, 127.7, 72.9, 63.4, 61.1, 57.7, 57.7, 50.2, 49.1, 48.9, 43.7, 41.4, 10.4. HRMS: calculated for C_21_H_25_N_5_O_6_·H^+^, 444.1877; found, 444.1891. HPLC (254 nm, method B) 2.4 min, 100%.

*((11S,11aR)-5-(2-hydroxyethyl)-6,9-dioxo-3-phenyl-4,5,6,7,9,11,11a,12-octahydrooxazolo[3,4-a][1,2,3]triazolo[1,5-d][1,4,7]triazonin-11-yl)methyl 2-phenylacetate* (**3j**). Synthesized from ester **3d** (0.076 g, 0.127 mmol) using general procedure B. The crude product was purified by column chromatography (80:20:2, EtOAc:hexanes:Et_3_N) to afford 0.015 g (23%) of **3j** as a white solid, mp (decomposed) 186.9–187.5 °C; [α]_D_^25^ −58.8 (*c* 0.08, CHCl_3_). ^1^H NMR (500 MHz, CDCl_3_) δ 7.54–7.26 (m, 10H), 5.11 (d, *J* = 17.8 Hz, 1H), 4.87 (d, *J* = 16.8 Hz, 1H), 4.71 (d, *J* = 18.0 Hz, 1H), 4.68–4.59 (m, 2H), 4.47 (q, *J* = 3.8 Hz, 1H), 4.30 (t, *J* = 3.2 Hz, 2H), 3.91–3.86 (m, 1H), 3.76–3.68 (m, 1H), 3.68 (s, 2H), 3.67–3.59 (m, 1H), 3.51–3.45 (m, 2H), 3.32–3.24 (m, 1H). ^13^C NMR (125 MHz, CDCl_3_) δ 170.8, 169.6, 155.5, 147.1, 133.2, 129.5, 129.3, 129.0, 128.8, 128.4, 128.2, 127.6, 72.8, 63.4, 60.7, 57.5, 50.3, 49.4, 48.7, 44.4, 41.2. HRMS: calculated for C_26_H_27_N_5_O_6_·H^+^, 506.2034; found, 506.2050. HPLC (254 nm, method B) 2.7 min, 100%.

*((11R,11aS)-5-(2-hydroxyethyl)-3-methyl-6,9-dioxo-4,5,6,7,9,11,11a,12-octahydrooxazolo[3,4-a][1,2,3]triazolo[1,5-d][1,4,7]triazonin-11-yl)methyl 2-phenylacetate* (**3k**). Synthesized from ester **3f** (0.052 g, 0.098 mmol) using general procedure B. The crude product was purified by column chromatography (3% iPrOH in EtOAc) to afford 0.018 g (42%) of **3k** as a white solid, mp 126.2–128.0 °C; [α]_D_^25^ +99.2 (*c* 0.12, CHCl_3_); ^1^H NMR (500 MHz, CDCl_3_) δ 7.41–7.23 (m, 5H), 4.89–4.80 (m, 2H), 4.60–4.45 (m, 3H), 4.45–4.40 (m, 1H), 4.29 (d, *J* = 3.5 Hz, 2H), 3.84–3.77 (m, 2H), 3.77–3.70 (m, 1H), 3.67 (s, 2H), 3.59 (dd, *J* = 14.2, 4.2 Hz, 1H), 3.44 (d, *J* = 16.2 Hz, 1H), 3.26–3.17 (m, 1H), 2.28 (s, 3H). ^13^C NMR (125 MHz, CDCl_3_) δ 170.9, 169.4, 155.6, 142.9, 133.3, 129.4, 129.1, 128.4, 127.7, 72.9, 63.4, 61.1, 57.7, 57.7, 50.2, 49.1, 48.9, 43.7, 41.4, 10.4. HRMS: calculated for C_21_H_25_N_5_O_6_·H^+^, 444.1877; found, 444.1891. HPLC (254 nm, method B) 2.4 min, 100%.

*(11R,11aS)-5-(2-hydroxyethyl)-6,9-dioxo-3-phenyl-4,5,6,7,9,11,11a,12-octahydrooxazolo[3,4-a][1,2,3]triazolo[1,5-d][1,4,7]triazonin-11-yl)methyl 2-phenylacetate* (**3l**). Synthesized from ester **3h** (0.052 g, 0.084 mmol) using the general procedure for debenzylation. The crude product was purified by column chromatography (85% EtOAc in hexanes) to afford 0.032 g (73%) of **3l** as a white solid, mp (decomposed) 186.9–187.5 °C; [α]_D_^25^ +58.8 (*c* 0.09, CHCl_3_); ^1^H NMR (500 MHz, CDCl_3_) δ 7.54–7.26 (m, 10H), 5.11 (d, *J* = 17.8 Hz, 1H), 4.87 (d, *J* = 16.8 Hz, 1H), 4.71 (d, *J* = 18.0 Hz, 1H), 4.68–4.59 (m, 2H), 4.47 (q, *J* = 3.8 Hz, 1H), 4.30 (t, *J* = 3.2 Hz, 2H), 3.91–3.86 (m, 1H), 3.76–3.68 (m, 1H), 3.68 (s, 2H), 3.67–3.59 (m, 1H), 3.51–3.45 (m, 2H), 3.32–3.24 (m, 1H); ^13^C NMR (125 MHz, CDCl_3_) δ 170.8, 169.6, 155.5, 147.1, 133.2, 129.5, 129.3, 129.0, 128.8, 128.4, 128.2, 127.6, 72.9, 63.4, 60.7, 57.5, 50.3, 49.4, 48.7, 44.4, 41.2. HRMS: calculated for C_26_H_27_N_5_O_6_·H^+^, 506.2034; found, 506.2050. HPLC (254 nm, method B) 2.7 min, 100%.

### 3.4. General Procedure C: Alkylation of Oxazolidinone

Enantiopure azide **5** [15] (100 mol %) was dissolved in dry THF (0.55 M), cooled to 0 °C, and stirred under argon. In one portion, 60% NaH (150 mol %) was added and the solution was warmed to room temperature and stirred for one hour. After one hour, the solution was cooled back to 0 °C, and α-chloroamide **6** (120 mol %) dissolved in dry THF (1.0 M) was added. The solution was once again warmed to room temperature and stirred until the reaction was shown to have reached completion by TLC (2–4 h is typical). The reaction was quenched with saturated aqueous NH_4_Cl, and the THF was removed in vacuo. The mixture was then diluted with water, and extracted thrice with EtOAc. The combined organic phases were washed twice with brine, dried with MgSO_4_, filtered, and concentrated in vacuo. The crude product was purified by column chromatography to afford the pure azidoalkyne product (**4**) as a white solid.

*2-((4R,5S)-4-(azidomethyl)-2-oxo-5-((trityloxy)methyl)oxazolidin-3-yl)-N-benzyl-N-(but-2-yn-1-yl)acetamide* (**4a**). Synthesized from enantiopure azide **5a** (0.51 g, 1.23 mmol) and **6a** (0.35 g, 1.48 mmol) using general procedure C. The crude product was purified by column chromatography (22.5% EtOAc in hexanes) to afford 0.47 g (63%) of **4a** as a white solid, mp 52.4–56.5 °C; [α]_D_^25^ +4.5 (*c* 0.36, CHCl_3_); ^1^H NMR (500 MHz, CDCl_3_) δ 7.47–7.22 (m, 20H), 4.79–4.52 (m, 2H), 4.33 (dd, *J* = 62.4, 16.9 Hz, 1H), 4.22–3.82 (m, 5H), 3.64–3.35 (m, 4H), 1.80 (d, *J* = 12.0 Hz, 3H). ^13^C NMR (125 MHz, CDCl_3_) δ 167.1, 166.9, 157.8, 143.4, 136.3, 135.7, 129.1, 128.7, 128.6, 128.4, 128.0, 127.7, 127.3, 126.6, 87.3, 81.3, 80.5, 75.7, 73.3, 72.8, 64.1, 57.6, 52.2, 52.1, 49.4, 48.9, 44.5, 44.3, 36.2, 35.4, 3.5, 3.5. HRMS: calculated for C_37_H_35_N_5_O_4_·Na^+^, 636.2581; found, 636.2587; HPLC (254 nm, method B) 8.1 min, 99%.

*2-((4R,5S)-4-(azidomethyl)-2-oxo-5-((trityloxy)methyl)oxazolidin-3-yl)-N-(2-(benzyloxy)ethyl)-N-(but-2-yn-1-yl)acetamide* (**4b**). Synthesized from enantiopure azide **5a** (0.75 g, 1.81 mmol) and **6b** (0.61 g, 2.17 mmol) using general procedure C. The crude product was purified by column chromatography (25% EtOAc in hexanes) to afford 0.55 g (46%) of **4b** as a white solid, mp 45.9–48.3 °C; [α]_D_^25^ +10.5 (*c* 0.32, CHCl_3_). ^1^H NMR (500 MHz, CDCl_3_) δ 7.46–7.40 (m, 7H), 7.34–7.29 (m, 11H), 7.24 (d, *J* = 7.3 Hz, 2H), 4.55–4.44 (m, 2H), 4.38–4.28 (m, 1H), 4.27–4.11 (m, 2H), 4.13–4.02 (m, 2H), 3.85 (dd, *J* = 57.6, 5.3 Hz, 1H), 3.77–3.52 (m, 4H), 3.38 (ddd, *J* = 27.8, 10.1, 5.3 Hz, 3H), 3.06 (ddd, *J* = 67.2, 13.2, 4.1 Hz, 1H), 1.83–1.76 (m, 3H); ^13^C NMR (125 MHz, CDCl_3_) δ 167.5, 158.0, 143.6, 143.4, 137.8, 128.8, 128.7, 128.2, 128.1, 127.5, 127.4, 87.4, 80.3, 78.0, 75.8, 73.8, 67.7, 64.4, 57.6, 54.0, 51.0, 46.4, 35.2, 3.6. HRMS: calculated for C_39_H_39_N_5_O_5_·Na^+^, 680.2843; found, 680.2843. HPLC (254 nm, method B) 9.9 min, 93%.

*2-((4R,5S)-4-(azidomethyl)-2-oxo-5-((trityloxy)methyl)oxazolidin-3-yl)-N-benzyl-N-(3-phenylprop-2-yn-1-yl)acetamide* (**4c**). Synthesized from enantiopure azide **5a** (0.45 g, 1.09 mmol) and **6c** (0.39 g, 1.3 mmol) using general procedure C. The crude product was purified by column chromatography (20% EtOAc in hexanes) to afford 0.63 g (86%) of **4c** as a white solid, mp 55.6–66.0 °C; [α]_D_^25^ +2.9 (*c* 0.39, EtOAc); ^1^H NMR (500 MHz, CDCl_3_) δ 7.46–7.21 (m, 25H), 4.84–4.63 (m, 2H), 4.58–4.44 (m, 1H), 4.44–4.14 (m, 3H), 4.04–3.89 (m, 2H), 3.54 (ddd, *J* = 46.9, 12.8, 3.7 Hz, 1H), 3.48–3.37 (m, 3H). ^13^C NMR (125 MHz, CDCl_3_) δ 167.3, 157.9, 143.4, 133.2, 131.7, 130.0, 129.1, 128.7, 128.6, 128.3, 127.9, 127.3, 126.7, 87.3, 85.2, 84.6, 83.6, 82.9, 75.8, 64.1, 57.7, 52.2, 49.5, 44.5, 36.9, 35.9. HRMS: calculated for C_42_H_37_N_5_O_4_·Na^+^, 698.2739; found, 698.2749; HPLC (254 nm, method B) 10.0 min, 95%.

*2-((4R,5S)-4-(azidomethyl)-2-oxo-5-((trityloxy)methyl)oxazolidin-3-yl)-N-(2-(benzyloxy)ethyl)-N-(3-phenylprop-2-yn-1-yl)acetamide* (**4d**). Synthesized from enantiopure azide **5a** (0.66 g, 1.59 mmol) and **6d** (0.65 g, 1.9 mmol) using general procedure C. The crude product was purified by column chromatography (30% EtOAc in hexanes) to afford 0.40 g (60%) of **4d** as a white solid, mp 50.2–52.9 °C; [α]_D_^25^ +7.4 (*c* 0.29, CHCl_3_). ^1^H NMR (500 MHz, CDCl_3_) δ 7.47–7.37 (m, 8H), 7.31 (dq, *J* = 7.5, 4.7 Hz, 14H), 7.26–7.21 (m, 3H), 4.56–4.44 (m, 3H), 4.44–4.32 (m, 2H), 4.26–4.16 (m, 1H), 4.16–4.06 (m, 1H), 3.92–3.62 (m, 5H), 3.58–3.34 (m, 3H), 3.07 (ddd, *J* = 64.1, 13.2, 4.2 Hz, 1H). ^13^C NMR (125 MHz, CDCl_3_) δ 167.5, 157.9, 143.6, 137.7, 131.8, 128.7, 128.6, 128.5, 128.5, 128.4, 128.1, 128.0, 127.7, 127.3, 87.3, 84.3, 84.1, 75.7, 73.8, 67.7, 64.3, 57.6, 50.9, 46.6, 43.9, 35.6. HRMS: calculated for C_44_H_41_N_5_O_5_·Na^+^, 742.2999; found, 742.3037. HPLC (254 nm, method B) 10.4 min, 93%.

*2-((4S,5R)-4-(azidomethyl)-2-oxo-5-((trityloxy)methyl)oxazolidin-3-yl)-N-benzyl-N-(but-2-yn-1-yl)acetamide* (**4e**)**.** Synthesized from enantiopure azide **5b** (0.51 g, 1.22 mmol) and **6a** (0.35 g, 1.47 mmol) using general procedure C. The crude product was purified by column chromatography (22.5% EtOAc in hexanes) to afford 0.43 g (58%) of **4e** as a white solid, mp 52.4–56.5 °C; [α]_D_^25^ −4.6 (*c* 0.35, CHCl_3_). ^1^H NMR (500 MHz, CDCl_3_) δ 7.47–7.22 (m, 20H), 4.79–4.52 (m, 2H), 4.33 (dd, *J* = 62.4, 16.9 Hz, 1H), 4.22–3.82 (m, 5H), 3.64–3.35 (m, 4H), 1.80 (d, *J* = 12.0 Hz, 3H). ^13^C NMR (125 MHz, CDCl_3_) δ 167.1, 166.9, 157.8, 143.4, 136.3, 135.7, 129.1, 128.7, 128.6, 128.4, 128.0, 127.7, 127.3, 126.6, 87.3, 81.3, 80.5, 75.7, 73.3, 72.8, 64.1, 57.6, 52.2, 52.1, 49.4, 48.9, 44.5, 44.3, 36.2, 35.4, 3.5, 3.5. HRMS: calculated for C_37_H_35_N_5_O_4_·Na^+^, 636.2581; found, 636.2587; HPLC (254 nm, method B) 8.1 min, 99%.

*2-((4S,5R)-4-(azidomethyl)-2-oxo-5-((trityloxy)methyl)oxazolidin-3-yl)-N-(2-(benzyloxy)ethyl)-N-(but-2-yn-1-yl)acetamide* (**4f**). Synthesized from enantiopure azide **5b** (0.75 g, 1.81 mmol) and **6c** (0.61 g, 2.17 mmol) using the general procedure for making azidoalkynes. The crude product was purified by column chromatography (30% EtOAc in hexanes) to afford 0.91 g (76%) of **4f** as a white solid, mp 45.9–48.3 °C; [α]_D_^25^ −10.2 (*c* 0.33, CHCl_3_); ^1^H NMR (500 MHz, CDCl_3_) δ 7.46–7.40 (m, 7H), 7.34–7.29 (m, 11H), 7.24 (d, *J* = 7.3 Hz, 2H), 4.55–4.44 (m, 2H), 4.38–4.28 (m, 1H), 4.27–4.11 (m, 2H), 4.13–4.02 (m, 2H), 3.85 (dd, *J* = 57.6, 5.3 Hz, 1H), 3.77–3.52 (m, 4H), 3.38 (ddd, *J* = 27.8, 10.1, 5.3 Hz, 3H), 3.06 (ddd, *J* = 67.2, 13.2, 4.1 Hz, 1H), 1.83–1.76 (m, 3H). ^13^C NMR (125 MHz, CDCl_3_) δ 167.5, 158.0, 143.6, 143.4, 137.8, 128.8, 128.7, 128.2, 128.1, 127.5, 127.4, 87.4, 80.3, 78.0, 75.8, 73.8, 67.7, 64.4, 57.6, 54.1, 51.0, 46.4, 35.2, 3.6. HRMS: calculated for C_39_H_39_N_5_O_5_·Na^+^, 680.2843; found, 680.2843; HPLC (254 nm, method B) 9.9 min, 93%.

*2-((4S,5R)-4-(azidomethyl)-2-oxo-5-((trityloxy)methyl)oxazolidin-3-yl)-N-benzyl-N-(3-phenylprop-2-yn-1-yl)acetamide* (**4g**). Synthesized from enantiopure azide **5b** (0.45 g, 1.09 mmol) and **6c** (0.39 g, 1.3 mmol) using general procedure C. The crude product was purified by column chromatography (5% EtOAc in toluene) to afford 0.69 g (93%) of **4g** as a white solid, mp 55.6–66.0 °C; [α]_D_^25^ −2.9 (*c* 0.38, EtOAc); ^1^H NMR (500 MHz, CDCl_3_) δ 7.46–7.21 (m, 25H), 4.84–4.63 (m, 2H), 4.58–4.44 (m, 1H), 4.44–4.14 (m, 3H), 4.04–3.89 (m, 2H), 3.54 (ddd, *J* = 46.9, 12.8, 3.7 Hz, 1H), 3.48–3.37 (m, 3H). ^13^C NMR (125 MHz, CDCl_3_) δ 167.3, 157.9, 143.4, 133.2, 131.7, 130.0, 129.1, 128.8, 128.6, 128.3, 127.9, 127.3, 126.7, 87.3, 85.2, 84.6, 83.6, 82.9, 75.8, 64.1, 57.7, 52.2, 49.5, 44.5, 36.9, 35.9. HRMS: calculated for C_42_H_37_N_5_O_4_·Na^+^, 698.2739; found, 698.2749; HPLC (254 nm, method B) 10.0 min, 95%.

*2-((4S,5R)-4-(azidomethyl)-2-oxo-5-((trityloxy)methyl)oxazolidin-3-yl)-N-(2-(benzyloxy)ethyl)-N-(3-phenylprop-2-yn-1-yl)acetamide* (**4h**). Synthesized from enantiopure azide **5b** (0.70 g, 1.69 mmol) and **6d** (0.70 g, 2.03 mmol) using general procedure C. The crude product was purified by column chromatography (30% EtOAc in hexanes) to afford 1.01 g (83%) of **4h** as a white solid, mp 50.2–52.9 °C; [α]_D_^25^ −7.6 (*c* 0.29, CHCl_3_); ^1^H NMR (500 MHz, CDCl_3_) δ 7.47–7.37 (m, 8H), 7.31 (dq, *J* = 7.5, 4.7 Hz, 14H), 7.26–7.21 (m, 3H), 4.56–4.44 (m, 3H), 4.44–4.32 (m, 2H), 4.26–4.16 (m, 1H), 4.16–4.06 (m, 1H), 3.92–3.62 (m, 5H), 3.58–3.34 (m, 3H), 3.07 (ddd, *J* = 64.1, 13.2, 4.2 Hz, 1H). ^13^C NMR (125 MHz, CDCl_3_) δ 167.5, 157.9, 143.6, 137.7, 131.8, 128.7, 128.6, 128.5, 128.5, 128.4, 128.1, 128.0, 127.7, 127.3, 87.3, 84.3, 84.1, 75.7, 73.8, 67.7, 64.3, 57.6, 50.9, 46.6, 43.9, 35.6. HRMS: calculated for C_44_H_41_N_5_O_5_·Na^+^, 742.2999; found, 742.3037; HPLC (254 nm, method B) 10.4 min, 93%.

### 3.5. General Procedure C: Preparation of α-Chloroamides

Secondary amine **7** (100 mol %) was dissolved in anhydrous CH_2_Cl_2_ (0.6 M). Freshly distilled Et_3_N (300 mol %) was added to the solution, which was then cooled to 0 °C. Freshly distilled chloroacetyl chloride (200 mol %) dissolved in anhydrous CH_2_Cl_2_ (5.5 M) was then added dropwise over 20 min. The solution was stirred for an additional 5 min at 0 °C, and was then warmed to room temperature. When the reaction was shown to be complete by TLC (typically 1–2 h), it was quenched with water and diluted with CH_2_Cl_2_. The layers were separated and the organic phase was then washed twice with water. The combined aqueous layers were then extracted twice with CH_2_Cl_2_. The combined organic layers were washed with brine, dried with MgSO_4_, filtered, and concentrated in vacuo. The crude product was then purified by column chromatography to afford the α-chloroamide product (**6**) as a dark-brown oil.

*N-benzyl-N-(but-2-yn-1-yl)-2-chloroacetamide* (**6a**). Synthesized from amine **7a** (0.30 g, 1.9 mmol) using general procedure C. The crude product was purified by column chromatography (10% EtOAc in hexanes) to afford 0.36 g (80%) of **6a**. ^1^H NMR (300 MHz, CDCl_3_) δ 7.42–7.20 (m, 5H), 4.72 (s, 1H), 4.69 (s, 1H), 4.24 (s, 1H), 4.18 (s, 1H), 4.10 (s, 1H), 3.94 (d, *J* = 2.6 Hz, 1H), 1.86–1.76 (m, 3H). ^13^C NMR (125 MHz, CDCl_3_) δ 167.0, 136.6, 129.3, 129.1, 129.0, 128.9, 128.9, 128.6, 128.4, 128.2, 128.2, 128.1, 128.0, 127.9, 127.8, 127.1, 81.7, 80.9, 73.4, 50.7, 49.2, 44.1, 42.9, 41.7, 37.4, 35.8, 3.7. HRMS: calculated for C_13_H_14_ClNO·H^+^, 236.0837; found, 236.0837. HPLC (254 nm, method B) 3.4 min, 100%.

*N-(2-(benzyloxy)ethyl)-N-(but-2-yn-1-yl)-2-chloroacetamide* (**6b**). Synthesized from **7b** (0.45 g, 2.21 mmol) using general procedure C. The crude product was purified by column chromatography (15% EtOAc in hexanes) to afford 0.37 g (59%) of **6b**. ^1^H NMR (500 MHz, CDCl_3_) δ 7.39–7.27 (m, 5H), 4.57 (d, *J* = 2.5 Hz, 2H), 4.28 (d, *J* = 2.0 Hz, 1H), 4.17 (d, *J* = 3.9 Hz, 4H), 4.11 (s, 1H), 3.29 (dd, *J* = 19.0, 7.6 Hz, 2H), 2.12–1.94 (m, 1H), 0.94 (dd, *J* = 21.5, 6.7 Hz, 6H); ^13^C NMR (125 MHz, CDCl_3_) δ 166.7, 166.3, 137.2, 136.9, 128.3, 128.2, 127.8, 127.8, 127.7, 80.8, 80.8, 80.5, 79.7, 71.7, 71.3, 57.2, 57.1, 54.8, 53.7, 41.3, 40.9, 38.1, 35.3, 26.9, 26.6, 19.8, 19.7; HRMS: calculated for C_15_H_18_ClNO_2_·H^+^, 280.1098; found, 280.1099; HPLC (254 nm, method B) 3.5 min, 100%.

*N-benzyl-2-chloro-N-(3-phenylprop-2-yn-1-yl)acetamide* (**6c**). Synthesized from **7c** (0.35 g, 1.59 mmol) using general procedure C. The crude product was purified by column chromatography (80% CH_2_Cl_2_ in hexanes) to afford 0.28 g (60%) of **6c.** ^1^H NMR (300 MHz, CDCl_3_) δ 7.45–7.25 (m, 10H), 4.78 (d, *J* = 7.1 Hz, 2H), 4.48 (s, 1H), 4.27 (d, *J* = 13.8 Hz, 2H), 4.14 (s, 1H); ^13^C NMR (125 MHz, CDCl_3_) δ 166.8, 136.0, 135.5, 131.7, 130.1, 129.1, 128.8, 128.8, 128.5, 128.4, 128.4, 128.3, 128.3, 128.1, 127.8, 126.8, 122.4, 121.9, 85.2, 84.6, 83.2, 82.6, 50.6, 49.1, 41.3, 37.5, 35.9; HRMS: calculated for C_18_H_16_ClNO·H^+^, 298.0993; found, 298.0993; HPLC (254 nm, method B) 4.9 min, 95%.

*N-(2-(benzyloxy)ethyl)-2-chloro-N-(3-phenylprop-2-yn-1-yl)acetamide* (**6d**). Synthesized from **7d** (0.70 g, 2.64 mmol) using general procedure C. The crude product was purified by column chromatography (15% EtOAc in hexanes) to afford 0.61 g (67%) of **6d**. ^1^H NMR (500 MHz, CDCl_3_) δ 7.40–7.27 (m, 10H), 4.53 (s, 2H), 4.49 (s, 2H), 4.26 (d, *J* = 11.1 Hz, 2H), 3.80–3.75 (m, 2H), 3.73–3.70 (m, 2H); ^13^C NMR (125 MHz, CDCl_3_) δ 166.9, 166.5, 137.9, 137.5, 131.6, 128.6, 128.4, 128.2, 127.7, 127.4, 122.4, 121.9, 84.1, 83.8, 83.3, 73.3, 73.1, 68.5, 67.3, 47.3, 46.7, 41.4, 39.8, 35.9; HRMS: calculated for C_20_H_20_ClNO_2_·H^+^, 342.1255; found, 342.1255; HPLC (254 nm, method B) 5.0, 87%.

### 3.6. General Procedure E: Intramolecular Dipolar Cyclization (**9**)

A solution of azidoalkyne (**4**) was dissolved in distilled toluene (0.008 M) was heated to reflux until TLC showed the reaction to have reached completion (18–48 h). The reaction was then concentrated in vacuo, and the crude product was purified by column chromatography to afford the pure triazole product (**9**) as a white solid.

*(11S,11aR)-5-benzyl-3-methyl-11-((trityloxy)methyl)-4,5,11a,12-tetrahydrooxazolo [3,4-a][1,2,3]triazolo[1,5-d][1,4,7]triazonine-6,9(7H,11H)-dione* (**8a**). Synthesized from azidoalkyne **4a** (0.29 g, 0.48 mmol) using general procedure E. The crude product was purified by column chromatography (40% EtOAc in hexanes) to afford 0.28 g (97%) of **8a** as a white solid, mp 168.0–169.0 °C; [α]_D_^25^ −32.0 (*c* 0.3, CHCl_3_); ^1^H NMR (500 MHz, CDCl_3_) δ 7.38 (d, *J* = 7.8 Hz, 6H), 7.35–7.29 (m, 9H), 7.29–7.24 (m, 3H), 7.19–7.11 (m, 2H), 5.08 (dd, *J* = 25.7, 15.3 Hz, 2H), 4.78 (d, *J* = 17.7 Hz, 1H), 4.49 (d, *J* = 15.4 Hz, 1H), 4.36 (q, *J* = 3.6 Hz, 1H), 4.30–4.21 (m, 2H), 4.01–3.96 (m, 1H), 3.94 (d, *J* = 16.5 Hz, 1H), 3.82 (d, *J* = 14.3 Hz, 1H), 3.49 (dd, *J* = 10.6, 3.7 Hz, 1H), 3.08 (dd, *J* = 10.6, 2.8 Hz, 1H), 2.17 (s, 3H); ^13^C NMR (125 MHz, CDCl_3_) δ 168.5, 156.4, 143.1, 134.6, 129.1, 128.8, 128.6, 128.5, 128.2, 128.0, 127.9, 127.5, 87.2, 74.6, 63.2, 57.9, 50.0, 49.5, 49.1, 41.1, 10.2. HRMS: calculated for C_37_H_35_N_5_O_4_·Na^+^, 636.2581; found, 636.2589; HPLC (254 nm, method B) 5.8 min, 94%.

*(11S,11aR)-5-(2-(benzyloxy)ethyl)-3-methyl-11-((trityloxy)methyl)-4,5,11a,12-tetrahydrooxazolo[3,4-a][1,2,3]triazolo[1,5-d][1,4,7]triazonine-6,9(7H,11H)-dione* (**8b**). Synthesized from azidoalkyne **4b** (0.50 g, 0.76 mmol) using general procedure E. The crude product was purified by column chromatography (60% EtOAc in hexanes) to afford 0.32 g (64%) of **8b** as a white solid, mp 209.0–211.0 °C; [α]_D_^25^ −25.0 (*c* 0.3, CHCl_3_); ^1^H NMR (500 MHz, CDCl_3_) δ 7.38–7.24 (m, 24H), 5.06 (d, *J* = 16.8 Hz, 1H), 4.89 (d, *J* = 17.7 Hz, 1H), 4.69 (d, *J* = 17.7 Hz, 1H), 4.49–4.37 (m, 2H), 4.36 (s, 3H), 3.95–3.91 (m, 1H), 3.85 (d, *J* = 16.2 Hz, 1H), 3.70–3.63 (m, 1H), 3.64–3.59 (m, 2H), 3.48 (dd, *J* = 10.7, 3.8 Hz, 1H), 3.28 (dt, *J* = 13.2, 6.2 Hz, 1H), 3.07 (dd, *J* = 10.8, 2.8 Hz, 1H), 2.22 (s, 3H); ^13^C NMR (125 MHz, CDCl_3_) δ 168.8, 156.4, 143.1, 142.6, 137.5, 128.6, 128.6, 128.5, 128.2, 128.1, 127.8, 127.5, 87.2, 74.6, 73.4, 68.4, 63.2, 57.9, 49.5, 49.2, 47.3, 43.5, 10.2; HRMS: calculated for C_39_H_39_N_5_O_5_·H^+^, 658.3024; found, 658.3050; HPLC (254 nm, method B) 8.6 min, 98%.

*(11R,11aS)-5-benzyl-3-phenyl-11-((trityloxy)methyl)-4,5,11a,12-tetrahydrooxazolo [3,4-a][1,2,3]triazolo[1,5-d][1,4,7]triazonine-6,9(7H,11H)-dione* (**8c**). Synthesized from azidoalkyne **4c** (0.29 g, 0.43 mmol) using general procedure E. The crude product was purified by column chromatography (30% EtOAc in hexanes) to afford 0.28 g (97%) of **8c** as a white solid, mp (decomposed) 241 °C; [α]_D_^25^ +3.1 (*c* 0.4, CHCl_3_); ^1^H NMR (500 MHz, CDCl_3_) δ 7.45–7.12 (m, 23H), 6.97–6.92 (m, 2H), 5.15 (d, *J* = 16.6 Hz, 1H), 5.05 (d, *J* = 17.9 Hz, 1H), 4.77 (d, *J* = 14.2 Hz, 1H), 4.57–4.49 (m, 2H), 4.37 (q, *J* = 3.5 Hz, 1H), 4.30 (dd, *J* = 15.4, 2.6 Hz, 1H), 4.06 (d, *J* = 14.2 Hz, 1H), 4.04–4.02 (m, 1H), 3.98 (d, *J* = 16.6 Hz, 1H), 3.50 (dd, *J* = 10.6, 3.8 Hz, 1H), 3.08 (dd, *J* = 10.7, 2.8 Hz, 1H); ^13^C NMR (125 MHz, CDCl_3_) δ 168.4, 156.4, 143.1, 134.2, 129.7, 128.9, 128.8, 128.8, 128.5, 128.5, 128.3, 127.6, 87.3, 74.6, 63.3, 57.8, 50.6, 49.7, 49.1, 42.4; HRMS: calculated for C_42_H_37_N_5_O_4_·Na^+^, 698.2738; found, 698.2752; HPLC (254 nm, method B) 8.0 min, 98%.

*(11R,11aS)-5-(2-(benzyloxy)ethyl)-3-phenyl-11-((trityloxy)methyl)-4,5,11a,12-tetrahydrooxazolo[3,4-a][1,2,3]triazolo[1,5-d][1,4,7]triazonine-6,9(7H,11H)-dione* (**8d**). Synthesized from azidoalkyne **4d** (0.53 g, 0.73 mmol) using general procedure E. The crude product was purified by column chromatography (35% EtOAc in hexanes) to afford 0.34 g (65%) of **8d** as a white solid, mp 211.0–213.0 °C; [α]_D_^25^ +16.7 (*c* 0.3, CHCl_3_); ^1^H NMR (500 MHz, CDCl_3_) δ 7.54 (dd, *J* = 7.5, 2.2 Hz, 2H), 7.44–7.36 (m, 9H), 7.35–7.23 (m, 12H), 7.11 (dd, *J* = 7.1, 2.5 Hz, 2H), 5.12 (t, *J* = 17.3 Hz, 2H), 4.90 (d, *J* = 17.9 Hz, 1H), 4.60–4.49 (m, 2H), 4.41 (q, *J* = 3.5 Hz, 1H), 4.24 (s, 2H), 3.99 (dt, *J* = 4.1, 2.0 Hz, 1H), 3.89 (d, *J* = 16.6 Hz, 1H), 3.56–3.46 (m, 4H), 3.45–3.35 (m, 1H), 3.09 (dd, *J* = 10.7, 2.8 Hz, 1H); ^13^C NMR (125 MHz, CDCl_3_) δ 169.0, 156.4, 146.9, 143.1, 137.4, 129.9, 129.0, 128.8, 128.7, 128.6, 128.5, 128.3, 128.2, 127.9, 127.7, 127.6, 87.3, 74.7, 73.3, 68.0, 63.3, 57.9, 49.8, 49.1, 47.6, 44.5; HRMS: calculated for C_44_H_41_N_5_O_5_·H^+^, 720.3180; found, 720.3161; HPLC (254 nm, method B) 8.7 min, 100%.

*(11R,11aS)-5-benzyl-3-methyl-11-((trityloxy)methyl)-4,5,11a,12-tetrahydrooxazolo [3,4-a][1,2,3]triazolo[1,5-d][1,4,7]triazonine-6,9(7H,11H)-dione* (**8e**). Synthesized from azidoalkyne **4a** (0.35 g, 0.58 mmol) using general procedure E. The crude product was purified by column chromatography (40% EtOAc in hexanes) to afford 0.24 g (68%) of **8e** as a white solid, mp 168.0–169.0 °C; [α]_D_^25^ +32.6 (*c* 0.3, CHCl_3_); ^1^H NMR (500 MHz, CDCl_3_) δ 7.38 (d, *J* = 7.8 Hz, 6H), 7.35–7.29 (m, 9H), 7.29–7.24 (m, 3H), 7.19–7.11 (m, 2H), 5.08 (dd, *J* = 25.7, 15.3 Hz, 2H), 4.78 (d, *J* = 17.7 Hz, 1H), 4.49 (d, *J* = 15.4 Hz, 1H), 4.36 (q, *J* = 3.6 Hz, 1H), 4.30–4.21 (m, 2H), 4.01–3.96 (m, 1H), 3.94 (d, *J* = 16.5 Hz, 1H), 3.82 (d, *J* = 14.3 Hz, 1H), 3.49 (dd, *J* = 10.6, 3.7 Hz, 1H), 3.08 (dd, *J* = 10.6, 2.8 Hz, 1H), 2.17 (s, 3H); ^13^C NMR (125 MHz, CDCl_3_) δ 168.5, 156.4, 143.1, 134.6, 129.1, 128.8, 128.6, 128.5, 128.2, 128.0, 127.9, 127.5, 87.2, 74.6, 63.2, 57.9, 50.0, 49.5, 49.1, 41.1, 10.2; HRMS: calculated for C_37_H_35_N_5_O_4_·Na^+^, 636.2581; found, 636.2589; HPLC (254 nm, method B) 5.8 min, 94%.

*(11R,11aS)-5-(2-(benzyloxy)ethyl)-3-methyl-11-((trityloxy)methyl)-4,5,11a,12-tetrahydrooxazolo[3,4-a][1,2,3]triazolo[1,5-d][1,4,7]triazonine-6,9(7H,11H)-dione* (**8f**). Synthesized from azidoalkyne **4b** (0.51 g, 0.78 mmol) using general procedure E. The crude product was purified by column chromatography (60% EtOAc in hexanes) to afford 0.32 g (61%) of **8f** as a white solid, mp 209.0–211.0 °C; [α]_D_^25^ +24.6 (*c* 0.3, CHCl_3_); ^1^H NMR (500 MHz, CDCl_3_) δ 7.38–7.24 (m, 24H), 5.06 (d, *J* = 16.8 Hz, 1H), 4.89 (d, *J* = 17.7 Hz, 1H), 4.69 (d, *J* = 17.7 Hz, 1H), 4.49–4.37 (m, 2H), 4.36 (s, 3H), 3.95–3.91 (m, 1H), 3.85 (d, *J* = 16.2 Hz, 1H), 3.70–3.63 (m, 1H), 3.64–3.59 (m, 2H), 3.48 (dd, *J* = 10.7, 3.8 Hz, 1H), 3.28 (dt, *J* = 13.2, 6.2 Hz, 1H), 3.07 (dd, *J* = 10.8, 2.8 Hz, 1H), 2.22 (s, 3H); ^13^C NMR (125 MHz, CDCl_3_) δ 168.8, 156.4, 143.1, 142.6, 137.5, 128.6, 128.6, 128.5, 128.2, 128.1, 127.8, 127.5, 87.2, 74.6, 73.4, 68.4, 63.2, 58.0, 49.5, 49.2, 47.3, 43.5, 10.21; HRMS: calculated for C_39_H_39_N_5_O_5_·H^+^, 658.3024; found, 658.3050; HPLC (254 nm, method B) 8.6 min, 98%.

*(11R,11aS)-5-benzyl-3-phenyl-11-((trityloxy)methyl)-4,5,11a,12-tetrahydrooxazolo [3,4-a][1,2,3]triazolo[1,5-d][1,4,7]triazonine-6,9(7H,11H)-dione* (**8g**). Synthesized from azidoalkyne **4c** (0.34 g, 0.51 mmol) using general procedure E. The crude product was purified by column chromatography (30% EtOAc in hexanes) to afford 0.25 g (71%) of **8g** as a white solid, mp (decomposed) 241 °C; [α]_D_^25^ −3.4 (*c* 0.4, CHCl_3_;); ^1^H NMR (500 MHz, CDCl_3_) δ 7.45–7.12 (m, 23H), 6.97–6.92 (m, 2H), 5.15 (d, *J* = 16.6 Hz, 1H), 5.05 (d, *J* = 17.9 Hz, 1H), 4.77 (d, *J* = 14.2 Hz, 1H), 4.57–4.49 (m, 2H), 4.37 (q, *J* = 3.5 Hz, 1H), 4.30 (dd, *J* = 15.4, 2.6 Hz, 1H), 4.06 (d, *J* = 14.2 Hz, 1H), 4.04–4.02 (m, 1H), 3.98 (d, *J* = 16.6 Hz, 1H), 3.50 (dd, *J* = 10.6, 3.8 Hz, 1H), 3.08 (dd, *J* = 10.7, 2.8 Hz, 1H); ^13^C NMR (125 MHz, CDCl_3_) δ 168.4, 156.4, 143.1, 134.2, 129.7, 128.9, 128.9, 128.8, 128.5, 128.5, 128.3, 127.6, 87.3, 74.6, 63.3, 57.8, 50.6, 49.7, 49.1, 42.4; HRMS: calculated for C_42_H_37_N_5_O_4_·Na^+^, 698.2738; found, 698.2752; HPLC (254 nm, method B) 8.0 min, 98%.

*(11R,11aS)-5-(2-(benzyloxy)ethyl)-3-phenyl-11-((trityloxy)methyl)-4,5,11a,12-tetrahydrooxazolo[3,4-a][1,2,3]triazolo[1,5-d][1,4,7]triazonine-6,9(7H,11H)-dione* (**8h**). Synthesized from azidoalkyne **4d** (0.50 g, 0.70 mmol) using general procedure E. The crude product was purified by column chromatography (40% EtOAc in hexanes) to afford 0.37 g (73%) of **8h** as a white solid, mp 211.0–213.0 °C; [α]_D_^25^ −16.3 (*c* 0.3, CHCl_3_); ^1^H NMR (500 MHz, CDCl_3_) δ 7.54 (dd, *J* = 7.5, 2.2 Hz, 2H), 7.44–7.36 (m, 9H), 7.35–7.23 (m, 12H), 7.11 (dd, *J* = 7.1, 2.5 Hz, 2H), 5.12 (t, *J* = 17.3 Hz, 2H), 4.90 (d, *J* = 17.9 Hz, 1H), 4.60–4.49 (m, 2H), 4.41 (q, *J* = 3.5 Hz, 1H), 4.24 (s, 2H), 3.99 (dt, *J* = 4.1, 2.0 Hz, 1H), 3.89 (d, *J* = 16.6 Hz, 1H), 3.56–3.46 (m, 4H), 3.45–3.35 (m, 1H), 3.09 (dd, *J* = 10.7, 2.8 Hz, 1H). ^13^C NMR (125 MHz, CDCl_3_) δ 169.0, 156.4, 146.9, 143.1, 137.4, 129.9, 129.0, 128.8, 128.7, 128.6, 128.5, 128.3, 128.2, 127.9, 127.7, 127.6, 87.3, 74.7, 73.3, 68.0, 63.3, 57.9, 49.8, 49.1, 47.6, 44.5. HRMS: calculated for C_44_H_41_N_5_O_5_·H^+^, 720.3180; found, 720.3161; HPLC (254 nm, method B) 8.7 min, 100%.

### 3.7. Transcription Assays

The T-box riboswitch in vitro transcription readthrough assay was conducted as previously described [25]. Triplicate reactions of 0.1 mM ligand, 30 nM tRNA, 5 mM spermidine, 0.05 U/uL RNAP, and 2.5% DMSO were run for 6 h and the data analyzed as previously described [25] to determine the Readthrough_max_. Unpaired, two-tailed *t* tests were conducted using Prism 9.0 (GraphPad) of each ligand reaction compared to the respective control in the absence of ligand. Percentage inhibition of tRNA-induced transcription readthrough was calculated as [1 − [(ΔRFU_tRNA+L_ − ΔRFU_L_)/(ΔRFU_tRNA+0_ − ΔRFU_0_)]] × 100, where ΔRFU_tRNA+0_ and ΔRFU_0_ are the average Readthrough_max_ values in the absence of ligand for the control reactions with or without tRNA, respectively, and ΔRFU_tRNA+L_ and ΔRFU_L_ are the respective average values in the presence of ligand.

### 3.8. Computational Docking

The computational docking was conducted similarly to that previously described using the Glide module of the Schrödinger software suite (version 2022-2, Schrödinger, New York, NY, USA) [15]. Ligands were initially built in ChemDraw 22.0.0.22, exported as .sdf files and then prepared for docking using Ligprep (Schrödinger) with the OPLS4 forcefield. The docking grid was generated from 1N53 PDB [26] using Glide with the OPLS2005 forcefield (non-default settings were: grid-centered on residues 2–15, 22–28 (numbering as in Figure 2); dock ligands ≤ 20 Å; ligand midpoint box 25 Å in x,y,z) and the ligands docked using Glide with the OPLS2005 forcefield (non-default settings were: standard precision docking; include aromatic H and halogens as hydrogen bond acceptors; keep at most 5 poses, no minimization).

## Data Availability

No new datasets were created. The original contributions presented in this study are included in the article/Supplementary Material. Further inquiries can be directed to the corresponding author.

## Supplementary Materials

The following supporting information can be downloaded at https://www.mdpi.com/article/10.3390/molecules31010029/s1. ^1^H and ^13^C NMR spectra for all new and tested compounds; HRMS spectra for tested compounds; Methods for the preparation of amines **7a**–**7d**; Mosher ester data for the precursor of aziridines **5a** and **5b**; Image of lowest E_model_ scores for ligand-docking the A1 helix. Ref. [28] is cited in Supplementary Materials.

## Abbreviations

The following abbreviations are used in this manuscript.

RNA	ribonucleic acid
tRNA	transfer ribonucleic acid
TLC	thin-layer chromatography
COVID	coronavirus disease
HPLC	high-pressure liquid chromatography
NMR	nuclear magnetic resonance
