# Supplementary material for: Cyclic Amide-Linked Oxazolidinone Triazoles as Inhibitors of the T-Box Riboswitch
Source: Molecules. 2025 Dec 22;31(1):29. doi: 10.3390/molecules31010029 (PMC12786550; doi:10.3390/molecules31010029)

Supplementary Material

Cyclic amide-linked oxazolidinone triazoles as inhibitors of the T-Box Riboswitch

Table of Contents

Mosher's ester data for the precursor to azides **5a** and **5b**. 1, 2
Preparation of propargyl amines 7a-7d...2, 3
NMR spectra for compounds for 1 enantiomer (**3a** (identical to **3e**), **3c** (identical to **3g**), **3i**, (identical to **3k**), **3j** (identical to **3l**) of all tested compounds...4 – 12
NMR spectra for all additional new compounds **3b** (identical to **3f**), **3d** (identical to **3h**), **4a** (identical to **4e**), **4b** (identical to **4f**), **4c** (identical to **4g**), **4d** (identical to **4h**) **9a** (identical to **9e**), **9b** (identical to **9f**), **9c** (identical to **9g**) **9d** (identical to **9h**), **6a**, **6b**, **6c**, **6d**.........................13 – 36
HRMS spectra for final compounds ....37 – 45
Docking Figures – A1 Helix 46

Mosher's ester data for the precursor to azides 5a and 5b

(*R*)-(*S*)-1-(trityloxy)but-3-en-2-yl 3,3,3-trifluoro-2-methoxy-2-phenylpropanoate (91a)

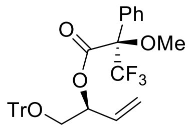

91a

Trityl protected alcohol **27a** (0.091 g, 0.27 mmol) was dissolved in 1 mL dry CH_2Cl_2 and stirred under argon. DMAP (0.013 g, 0.1 mmol) was added to the solution, followed by distilled Et_3N (0.09 mL, 0.65 mmol). This solution was then added to a vial containing (*R*)-(+)-α-Methoxy-α-(trifluoromethyl)phenylacetyl chloride (**90**) (0.074 mL, 0.40 mmol) kept at 0 °C. The reaction was warmed to room temperature and stirred for 3 hours. The solution was diluted with CH_2Cl_2 (10 mL) and washed with 1 M aq. HCl (10 mL), sat. aq. $NaHCO_3$ (10 mL), and brine (10 mL). The organic layer was dried with $MgSO_4$, filtered and concentrated *in vacuo*. The crude product (**91a**) was adequately pure to assess the enantiomeric purity of the product. ^{1}H NMR (500 MHz, C_6D_6) δ 7.80 (m, 2H), 7.43 – 7.38 (m, 6H), 7.06 (dd, J = 8.4, 6.8 Hz, 6H), 7.01 – 6.93 (m, 6H), 5.77 (ddd, J = 8.2, 6.8, 2.9 Hz, 1H), 5.41 (ddd, J = 17.4, 10.5, 6.9 Hz, 1H), 5.11 (dd, J = 17.2, 1.2 Hz, 1H), 4.85 (dd, J = 10.6, 1.2 Hz, 1H), 3.46 (s, 3H), 3.30 (dd, J = 10.3, 8.1 Hz, 1H), 3.16 (dd, J = 10.3, 3.0 Hz, 1H). ^{19}F NMR (500 MHz, C_6D_6) δ -71.61.

(*R*)-(*R*)-1-(trityloxy)but-3-en-2-yl 3,3,3-trifluoro-2-methoxy-2-phenylpropanoate (91b)

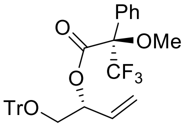

91b

Synthesized from **27b** (0.089 g, 0.27 mmol) using the same procedure described for the synthesis of **91a**. The crude product was adequately pure to assess the enantiomeric purity of the product. ^{1}H NMR (500 MHz, C_6D_6) δ 7.85 – 7.79 (m, 2H), 7.53 – 7.47 (m, 6H), 7.09 (t, J = 7.6 Hz, 6H), 7.02 (qd, J = 8.7, 2.3 Hz, 6H), 5.76 (td, J = 8.2, 7.7, 3.0 Hz, 1H), 5.32 (ddd, J = 17.1, 10.6, 6.3 Hz, 1H), 5.04 (dd, J = 17.3, 1.3 Hz, 1H), 4.81 (dd, J = 10.7, 1.2 Hz, 1H), 3.53 (s, 3H), 3.33 (dd, J = 10.5, 8.3 Hz, 1H), 3.24 (dd, J = 10.6, 2.8 Hz, 1H). ^{19}F NMR (500 MHz, C_6D_6) δ -71.31

^{19}F NMR spectra of the precursors to azides 5a and 5b

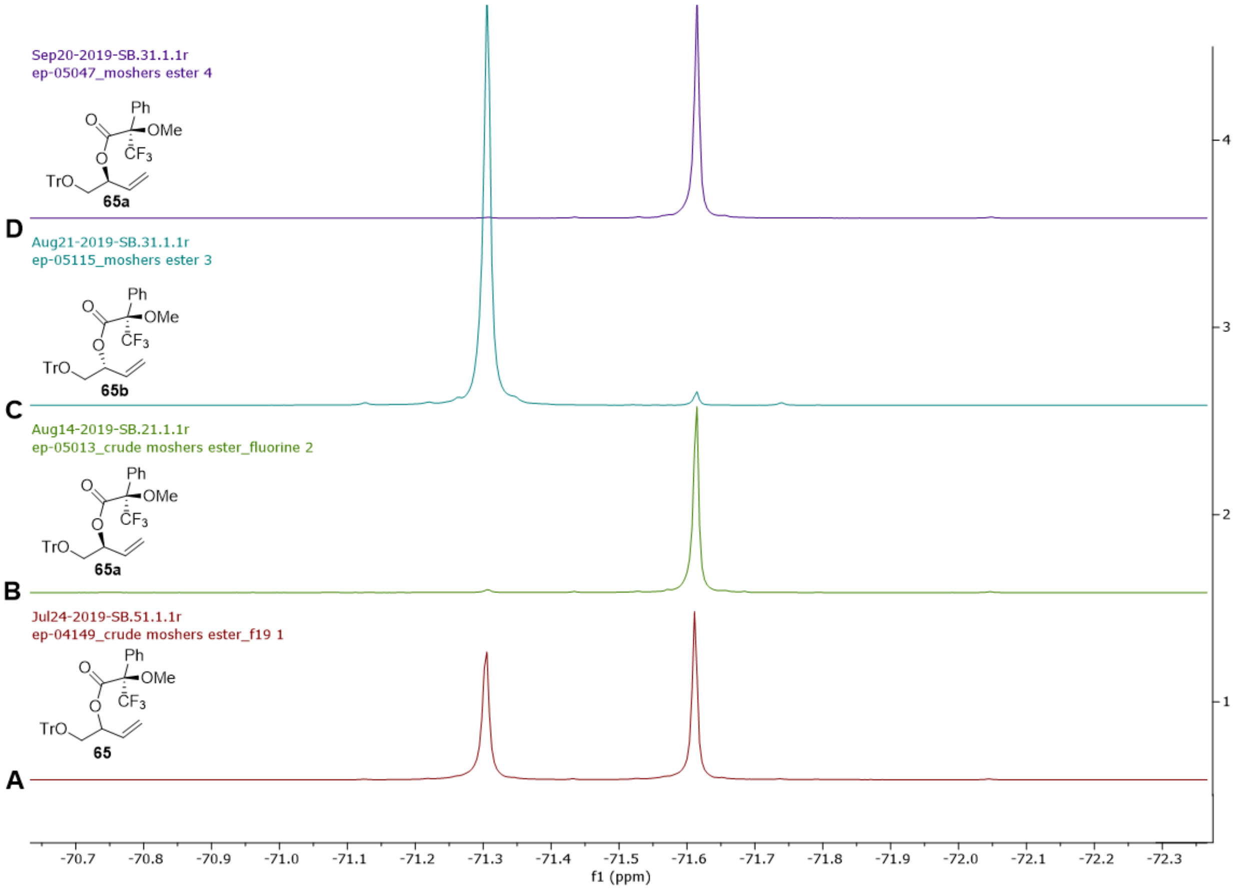

General Procedure D: Preparation of propargylic amines (7). The tosylate derived from the requisite commercially available alcohol[1] was dissolved in anhydrous DMF (0.5 M) and added dropwise to a flask containing the requisite freshly distilled primary amine (500 mol %), at 0 °C. The reaction was slowly

warmed to room temperature (the ice was not removed and was permitted to melt slowly) and was stirred overnight, at which point TLC indicated the reaction to be complete. The reaction was diluted with EtOAc, washed with saturated aqueous sodium bicarbonate (3x), dried over $MgSO_4$, filtered, and concentrated *in vacuo*. The crude product was purified by column chromatography to furnish the desired secondary amine (**7**) as a light-brown oil.

N-benzylbut-2-yn-1-amine (7a). Synthesized from benzyl amine (1.58 mL, 14.4 mmol) and the tosylate derived from but-2-yn-1-ol (0.54 g, 2.4 mmol) using general procedure D. The crude product was purified by column chromatography (30:70:2, EtOAc:Hexanes:Et_3N) to afford 0.31 g (80%) of **7a**. ^{1}H NMR (500 MHz, $CDCl_3$) δ 7.36 – 7.23 (m, 5H), 3.86 (s, 2H), 3.38 (q, *J* = 2.3 Hz, 2H), 1.84 (t, *J* = 2.4 Hz, 3H), 1.50 (s, 1H); ^{13}C NMR (125 MHz, $CDCl_3$) δ 139.9, 128.4, 128.4, 127.1, 79.2, 77.3, 52.6, 37.9, 3.5. All spectra matched that previously reported.[2]

N-(2-(benzyloxy)ethyl)but-2-yn-1-amine (7b). Synthesized from 2-(benzyloxy)ethanamine (4.04 g, 26.7 mmol) and the tosylate derived from but-2-yn-1-ol (1.20 g, 5.34 mmol) using general procedure D. The crude product was purified by column chromatography (30:70:2, EtOAc:Hexanes:Et_3N) to afford 0.50 g (46%) of **7b**. ^{1}H NMR (500 MHz, $CDCl_3$) δ 7.37 – 7.25 (m, 5H), 4.53 (s, 2H), 3.64 – 3.58 (m, 2H), 3.39 (q, *J* = 2.4 Hz, 2H), 2.91 – 2.85 (m, 2H), 1.81 (t, *J* = 2.4 Hz, 3H); ^{13}C NMR (125 MHz, $CDCl_3$) δ 138.3, 128.4, 127.8, 127.7, 79.1, 77.1, 73.2, 69.6, 48.3, 38.6, 3.5; HPLC (254 nm, method A) 2.8 min., 90%.

N-benzyl-3-phenylprop-2-yn-1-amine (7c). Synthesized from freshly distilled benzyl amine (1.6 mL, 14.8 mmol) and the tosylate derived from 3-phenylprop-2-yn-1-ol (0.71 g, 2.5 mmol) using general procedure D. The crude product was purified by column chromatography (30:70:2, EtOAc:Hexanes:Et_3N) to afford 0.41 g (76%) of **7c**. ^{1}H NMR (500 MHz, $CDCl_3$) δ 7.45 – 7.43 (m, 2H), 7.39 – 7.26 (m, 8H), 3.95 (s, 2H), 3.65 (s, 2H), 1.62 (s, 1H); ^{13}C NMR (125 MHz, $CDCl_3$) δ 139.7, 131.8, 128.6, 128.5, 128.4, 128.1, 127.2, 123.4, 87.7, 83.9, 52.7, 38.4. All spectra matched that previously reported.[3]

N-(2-(benzyloxy)ethyl)-3-phenylprop-2-yn-1-amine (7d). Synthesized from 2-(benzyloxy)ethanamine (2.90 g, 19.1 mmol) and the tosylate derived from 3-phenylprop-2-yn-1-ol (1.10 g, 3.83 mmol) using general procedure D. The crude product was purified by column chromatography (20:80:2, EtOAc:Hexanes:Et_3N) to afford 0.79 g (78%) of **7d**. ^{1}H NMR (500 MHz, $CDCl_3$) δ 7.42 – 7.27 (m, 10H), 4.54 (s, 2H), 3.68 (s, 2H), 3.65 (t, *J* = 5.1 Hz, 2H), 2.97 (t, *J* = 5.2 Hz, 2H), 2.25 (br s, 1H); ^{13}C NMR (125 MHz, $CDCl_3$) δ 138.3, 131.7, 128.4, 128.2, 127.9, 127.7, 127.6, 123.4, 87.6, 83.6, 73.2, 69.6, 48.3, 39.1; HPLC (254 nm, method A) 3.9 min., 99%.

(1) Oda, S.; Sam, B.; Krische, M. J. Hydroaminomethylation beyond Carbonylation: Allene-Imine Reductive Coupling by Ruthenium-Catalyzed Transfer Hydrogenation. . *Angew. Chem. Int. Ed.* **2015**, *54* (29), 8525-8528. DOI: https://doi.org/10.1002/anie.201503250.
(2) Hirata, Y.; Yukawa, T.; Kashihara, N.; Nakao, Y.; Hiyama, T. Nickel-Catalyzed Carbocyanation of Alkynes with Allyl Cyanides. *J. Am. Chem. Soc.* **2009**, *131* (31), 10964-10973. DOI: https://doi.org/10.1021/ja901374v.
(3) Wang, L.; Cai, C. Reusable Polymer-Anchored Amino Acid Copper Complex for the Synthesis of Propargylamines. *J. Chem. Res.* **2008**, *2008*, 538–541. https://doi.org/10.3184/030823408X349989

NMR Spectra for all tested compounds

3a

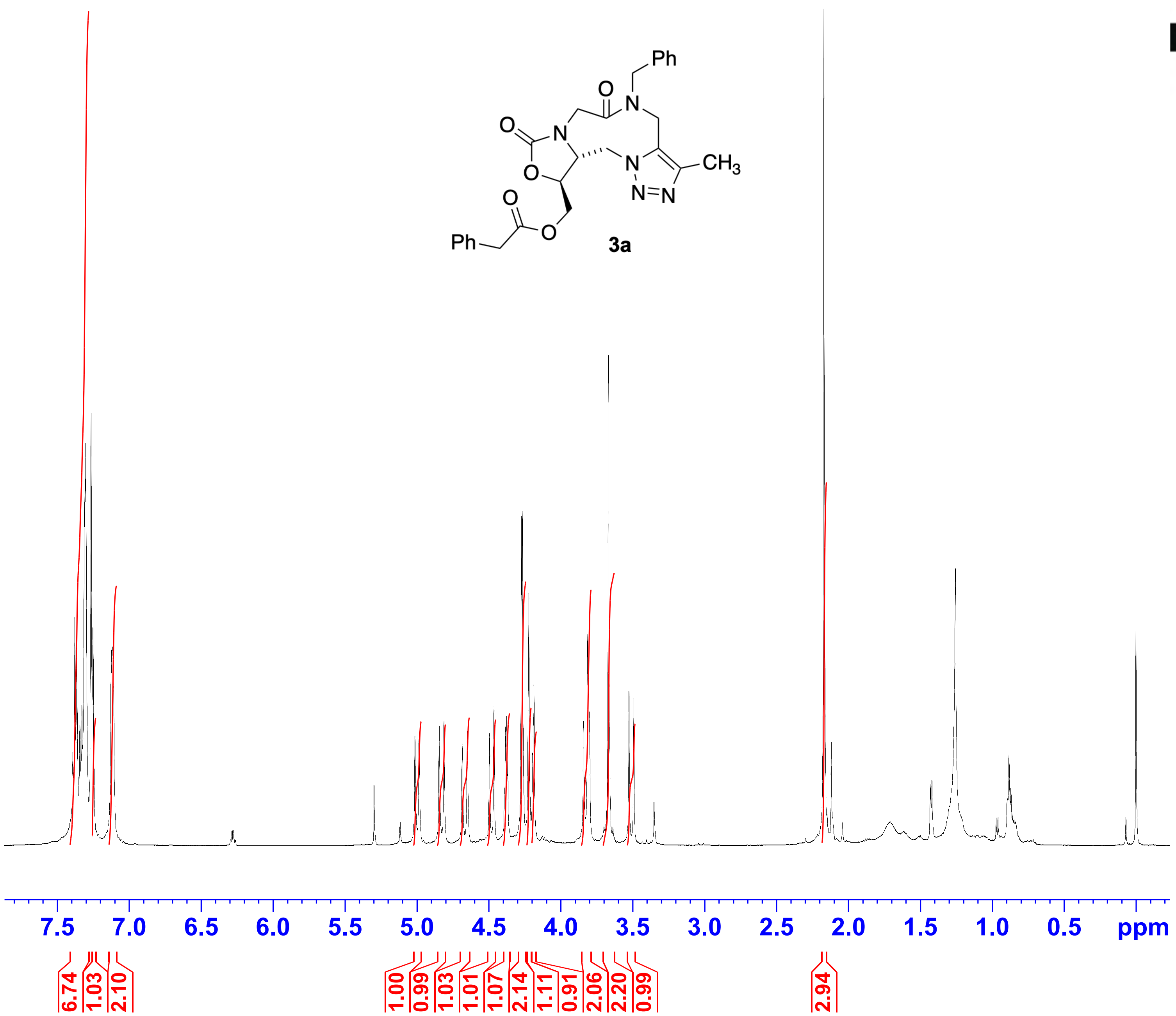


```
Current Data Parameters
NAME       Aug08-2020-SB
EXPNO                 80
PROCNO                 1

F2 - Acquisition Parameters
Date_           20200808
Time               15.17
INSTRUM            spect
PROBHD   5 mm PABBO BB/
PULPROG             zg30
SOLVENT            CDCl3
NS                    16
DS                     2
SWH            10000.000 Hz
FIDRES          0.152588 Hz
AQ             3.2767999 sec
RG                 87.21
DW                50.000 usec
DE                  6.50 usec
TE                 298.0 K
TD                 65536
D1            1.00000000 sec
TD0                    1

======== CHANNEL f1 ========
SFO1         500.1930889 MHz
NUC1                  1H
P1                 10.00 usec
PLW1         18.75000000 W

F2 - Processing parameters
SI                 65536
SF           500.1900102 MHz
WDW                   EM
SSB                    0
LB                  0.30 Hz
GB                     0
PC                  1.00
```

3a

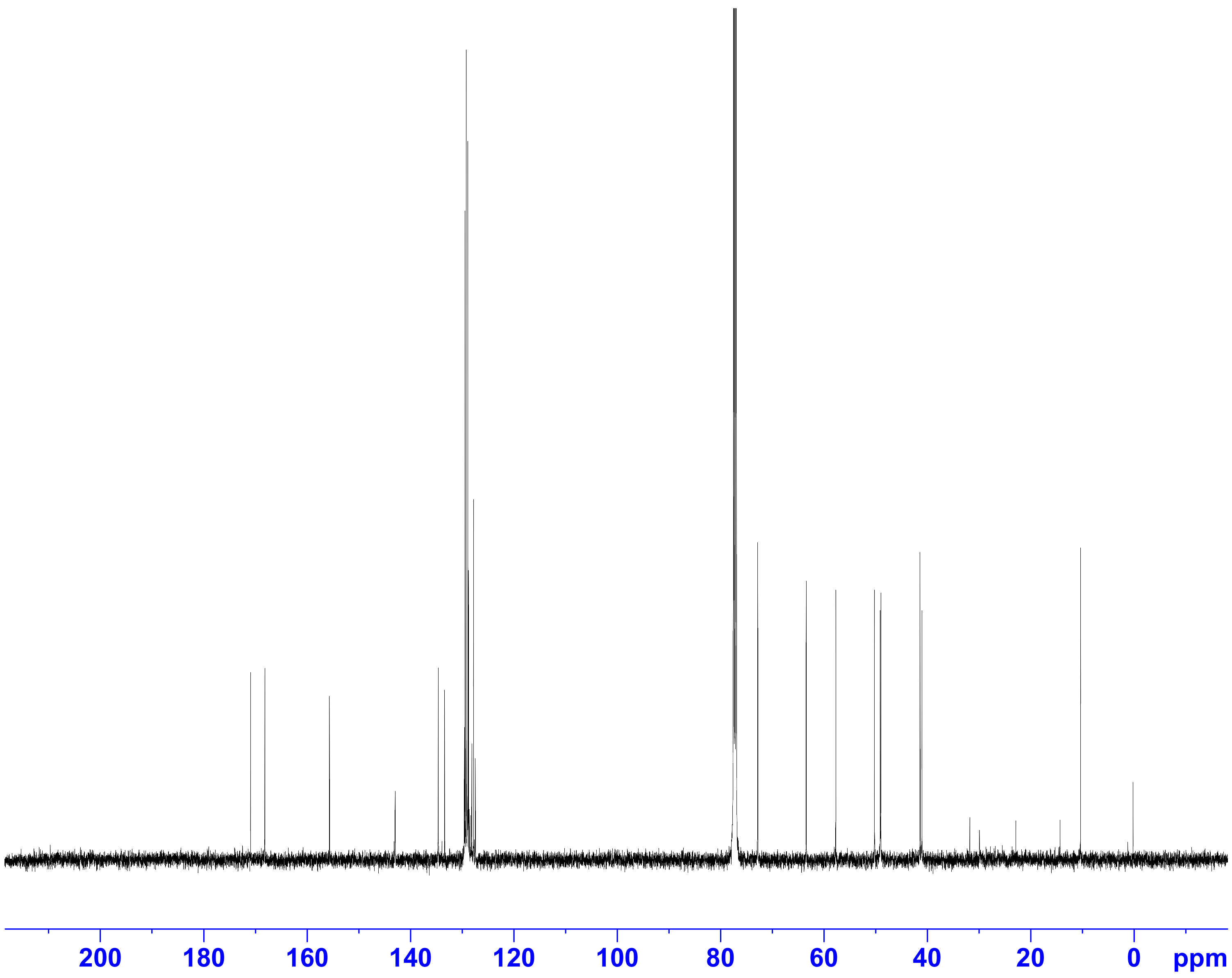

Current Data Parameters
NAME Nov07-2019-SB
EXPNO 10
PROCNO 1

F2 - Acquisition Parameters
Date_ 20191107
Time 13.53
INSTRUM spect
PROBHD 5 mm PABBO BB/
PULPROG zgpg30
SOLVENT CDCl3
NS 3072
DS 4
SWH 29761.904 Hz
FIDRES 0.454131 Hz
AQ 1.1010048 sec
RG 191.93
DW 16.800 usec
DE 6.50 usec
TE 298.0 K
TD 65536
D1 2.00000000 sec
D11 0.03000000 sec
TD0 1

======== CHANNEL f1 ========
SFO1 125.7854522 MHz
NUC1 13C
P1 9.65 usec
PLW1 78.00000000 W

======== CHANNEL f2 ========
SFO2 500.1920008 MHz
NUC2 1H
CPDPRG[2 waltz16
PCPD2 80.00 usec
PLW2 18.75000000 W
PLW12 0.29297000 W
PLW13 0.18750000 W

F2 - Processing parameters
SI 32768
SF 125.7728541 MHz
WDW EM
SSB 0
LB 1.00 Hz
GB 0
PC 1.40

3c

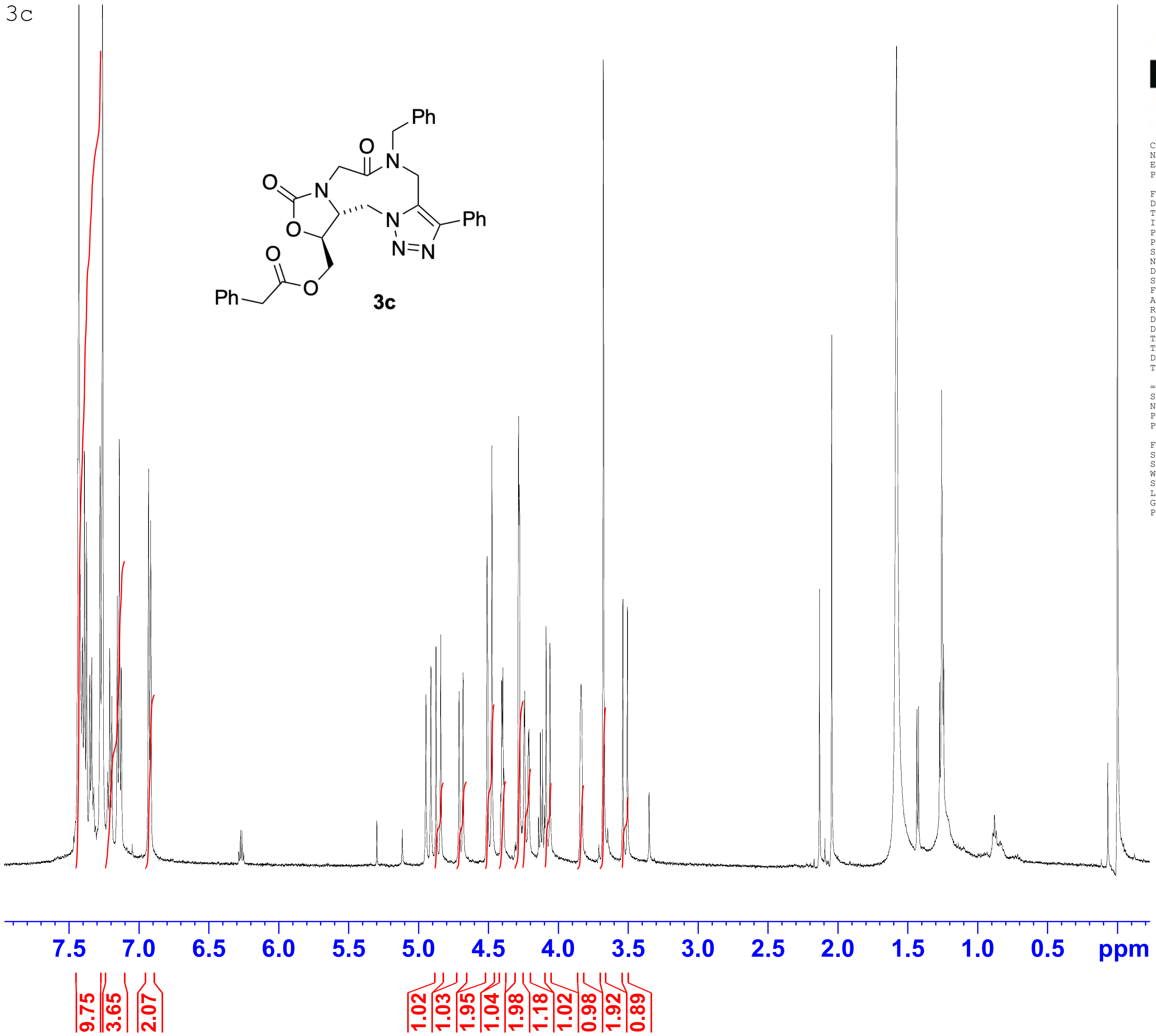


```
Current Data Parameters
NAME       Jul02-2020-SB
EXPNO                 70
PROCNO                 1

F2 - Acquisition Parameters
Date_           20200702
Time               15.09
INSTRUM            spect
PROBHD   5 mm PABBO BB/
PULPROG             zg30
SOLVENT            CDCl3
NS                    16
DS                     2
SWH            10000.000 Hz
FIDRES          0.152588 Hz
AQ             3.2767999 sec
RG                191.93
DW                50.000 usec
DE                  6.50 usec
TE                 298.0 K
TD                 65536
D1            1.00000000 sec
TD0                    1

======== CHANNEL f1 ========
SFO1         500.1930889 MHz
NUC1                  1H
P1                 10.00 usec
PLW1         18.75000000 W

F2 - Processing parameters
SI                 65536
SF           500.1900128 MHz
WDW                   EM
SSB                    0
LB                  0.30 Hz
GB                     0
PC                  1.00
```

3c

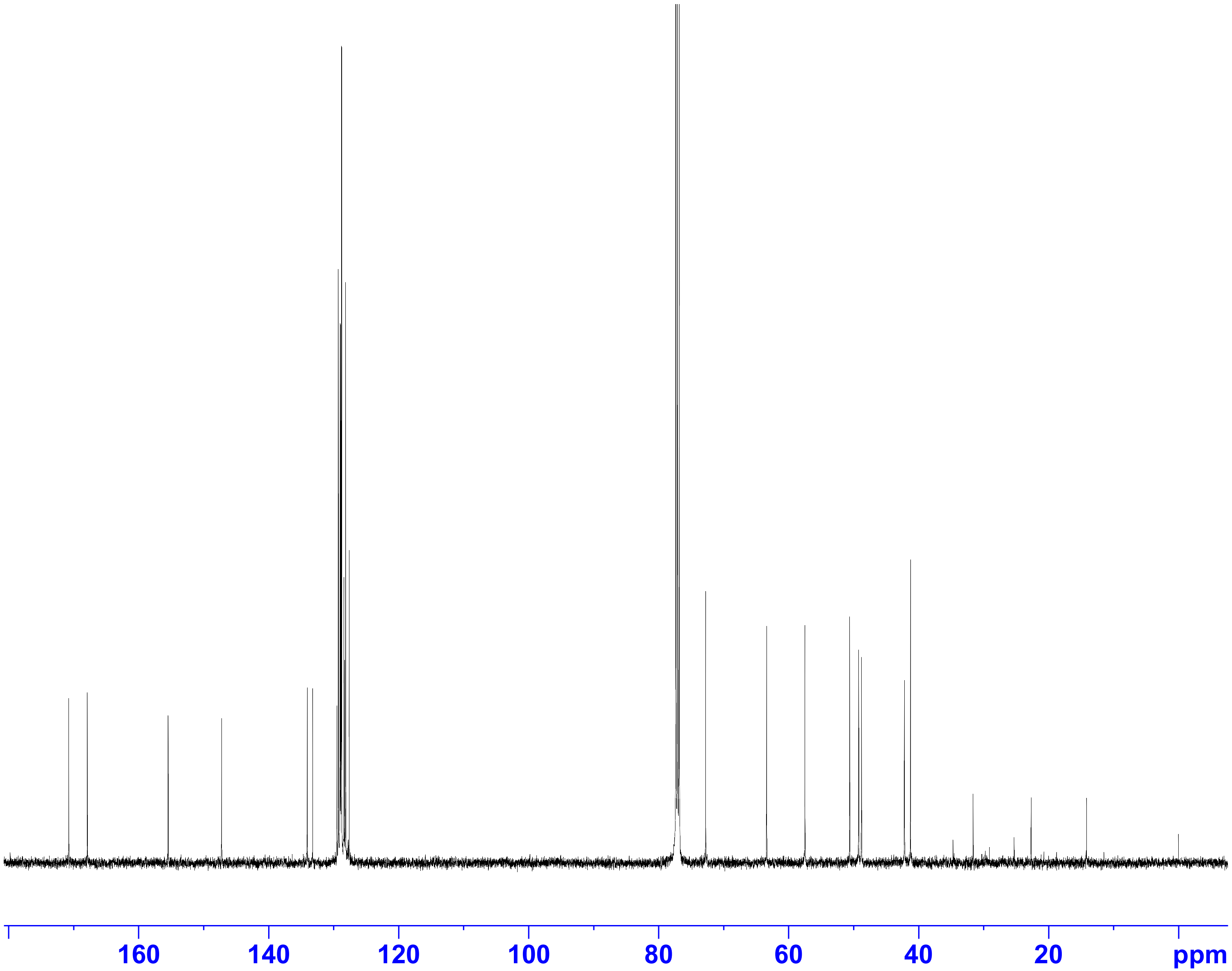


```
Current Data Parameters
NAME        Nov04-2019-SB
EXPNO                  10
PROCNO                  1

F2 - Acquisition Parameters
Date_            20191104
Time                14.16
INSTRUM             spect
PROBHD    5 mm PABBO BB/
PULPROG           zgpg30
SOLVENT             CDCl3
NS                   1280
DS                      4
SWH             29761.904 Hz
FIDRES           0.454131 Hz
AQ              1.1010048 sec
RG                 191.93
DW                 16.800 usec
DE                   6.50 usec
TE                  298.0 K
TD                  65536
D1             2.00000000 sec
D11            0.03000000 sec
TD0                     1

======== CHANNEL f1 ========
SFO1          125.7854522 MHz
NUC1                  13C
P1                   9.65 usec
PLW1          78.00000000 W

======== CHANNEL f2 ========
SFO2          500.1920008 MHz
NUC2                   1H
CPDPRG[2          waltz16
PCPD2               80.00 usec
PLW2          18.75000000 W
PLW12          0.29297000 W
PLW13          0.18750000 W

F2 - Processing parameters
SI                  32768
SF            125.7728755 MHz
WDW                    EM
SSB                     0
LB                   1.00 Hz
GB                      0
PC                   1.40
```

3i

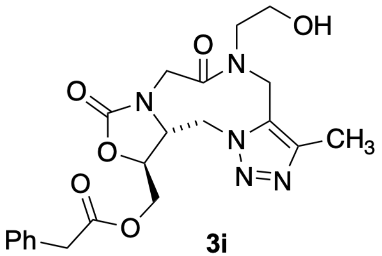

3i

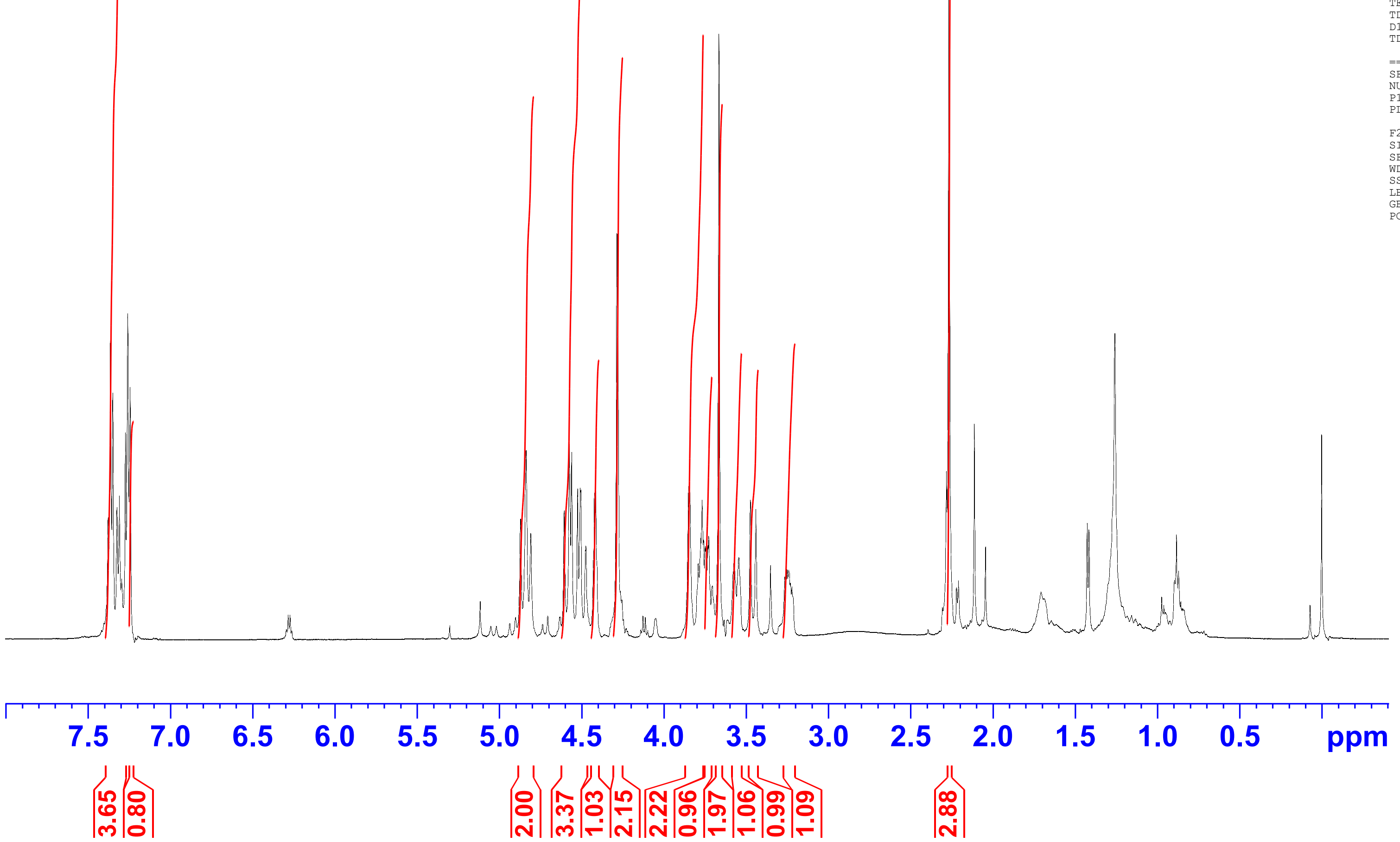


```
Current Data Parameters
NAME       Aug15-2020-SB
EXPNO                 10
PROCNO                 1

F2 - Acquisition Parameters
Date_           20200815
Time               14.10
INSTRUM            spect
PROBHD    5 mm PABBO BB/
PULPROG             zg30
SOLVENT            CDCl3
NS                    16
DS                     2
SWH            10000.000 Hz
FIDRES          0.152588 Hz
AQ             3.2767999 sec
RG                 77.72
DW                50.000 usec
DE                  6.50 usec
TE                 298.0 K
TD                 65536
D1            1.00000000 sec
TD0                    1

======== CHANNEL f1 ========
SFO1         500.1930889 MHz
NUC1                  1H
P1                 10.00 usec
PLW1         18.75000000 W

F2 - Processing parameters
SI                 65536
SF           500.1900054 MHz
WDW                   EM
SSB                    0
LB                  0.30 Hz
GB                     0
PC                  1.00
```

3i

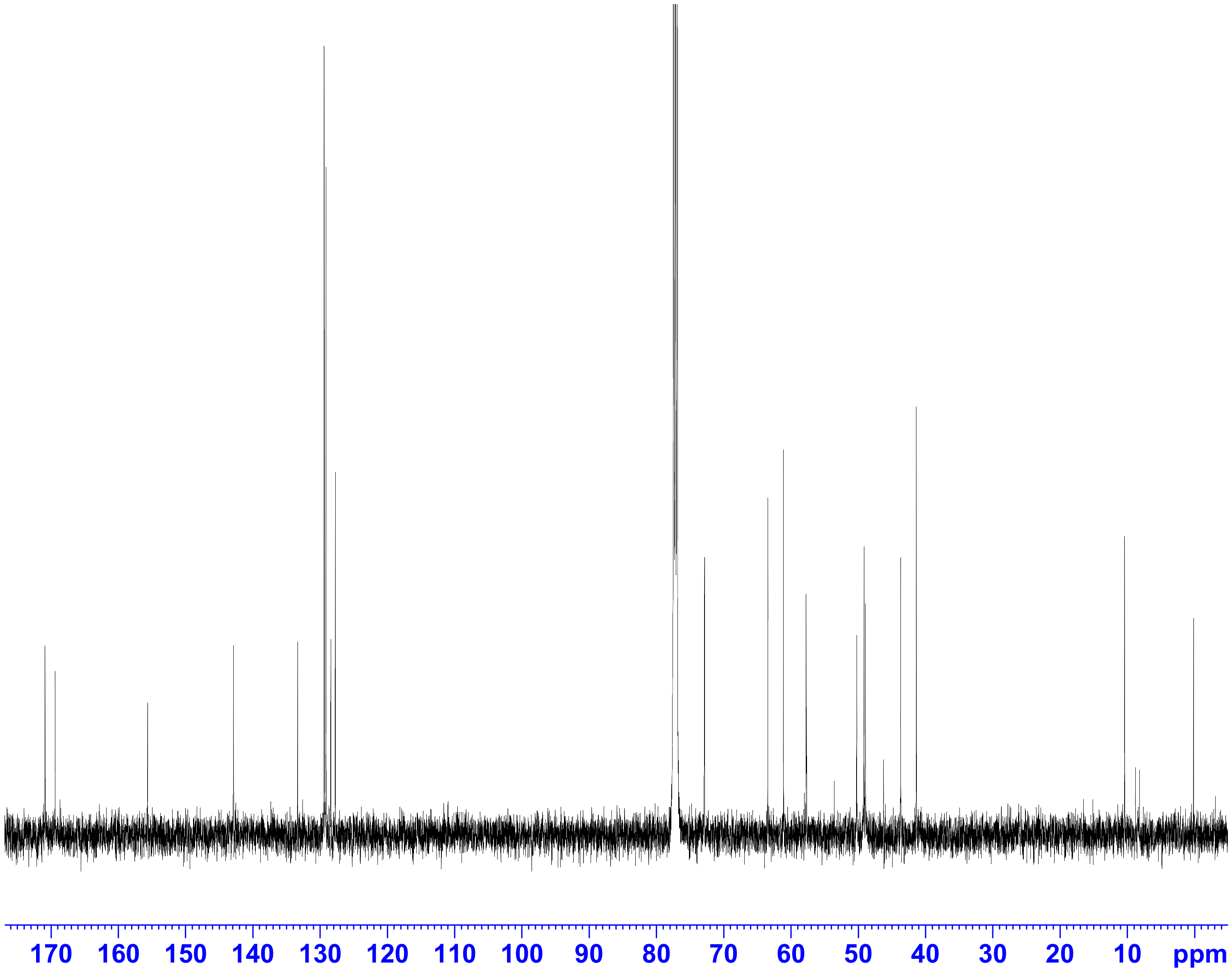


```
Current Data Parameters
NAME          Nov13-2019-SB
EXPNO                    11
PROCNO                    1

F2 - Acquisition Parameters
Date_              20191113
Time                  14.29
INSTRUM               spect
PROBHD    5 mm PABBO BB/
PULPROG             zgpg30
SOLVENT              CDCl3
NS                     2816
DS                        4
SWH              29761.904 Hz
FIDRES            0.454131 Hz
AQ               1.1010048 sec
RG                   191.93
DW                   16.800 usec
DE                     6.50 usec
TE                    298.0 K
TD                    65536
D1               2.00000000 sec
D11              0.03000000 sec
TD0                       1

======== CHANNEL f1 ========
SFO1           125.7854522 MHz
NUC1                    13C
P1                     9.65 usec
PLW1            78.00000000 W

======== CHANNEL f2 ========
SFO2           500.1920008 MHz
NUC2                     1H
CPDPRG[2            waltz16
PCPD2                 80.00 usec
PLW2            18.75000000 W
PLW12            0.29297000 W
PLW13            0.18750000 W

F2 - Processing parameters
SI                    32768
SF             125.7728598 MHz
WDW                      EM
SSB                       0
LB                     1.00 Hz
GB                        0
PC                     1.40
```

3j

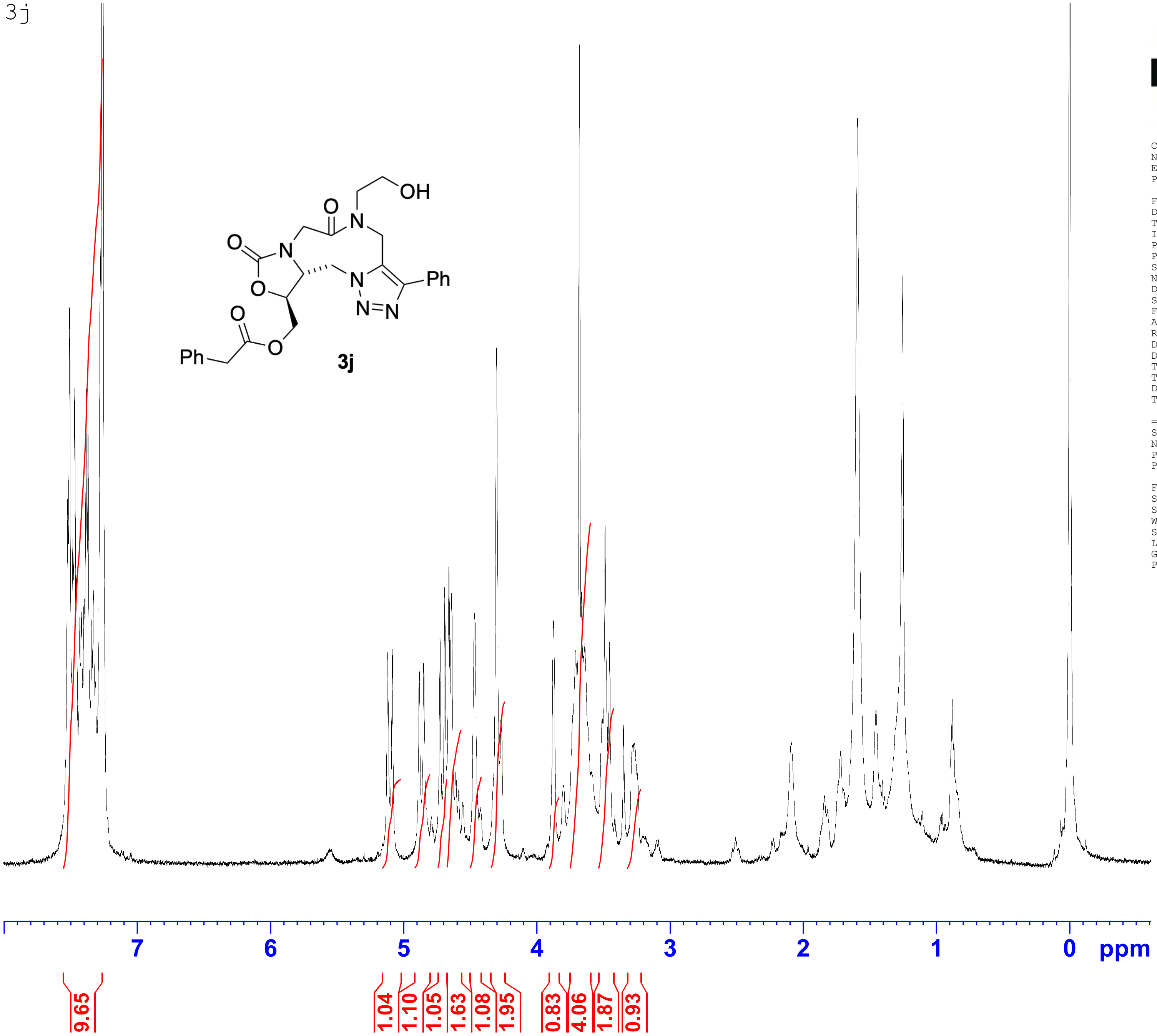


```
Current Data Parameters
NAME       Aug22-2020-SB
EXPNO                170
PROCNO                 1

F2 - Acquisition Parameters
Date_           20200822
Time               14.23
INSTRUM            spect
PROBHD   5 mm PABBO BB/
PULPROG             zg30
SOLVENT            CDCl3
NS                    16
DS                     2
SWH            10000.000 Hz
FIDRES          0.152588 Hz
AQ             3.2767999 sec
RG                191.93
DW                50.000 usec
DE                  6.50 usec
TE                 298.0 K
TD                 65536
D1            1.00000000 sec
TD0                    1

======== CHANNEL f1 ========
SFO1         500.1930889 MHz
NUC1                  1H
P1                 10.00 usec
PLW1         18.75000000 W

F2 - Processing parameters
SI                 65536
SF           500.1900128 MHz
WDW                   EM
SSB                    0
LB                  0.30 Hz
GB                     0
PC                  1.00
```

3j

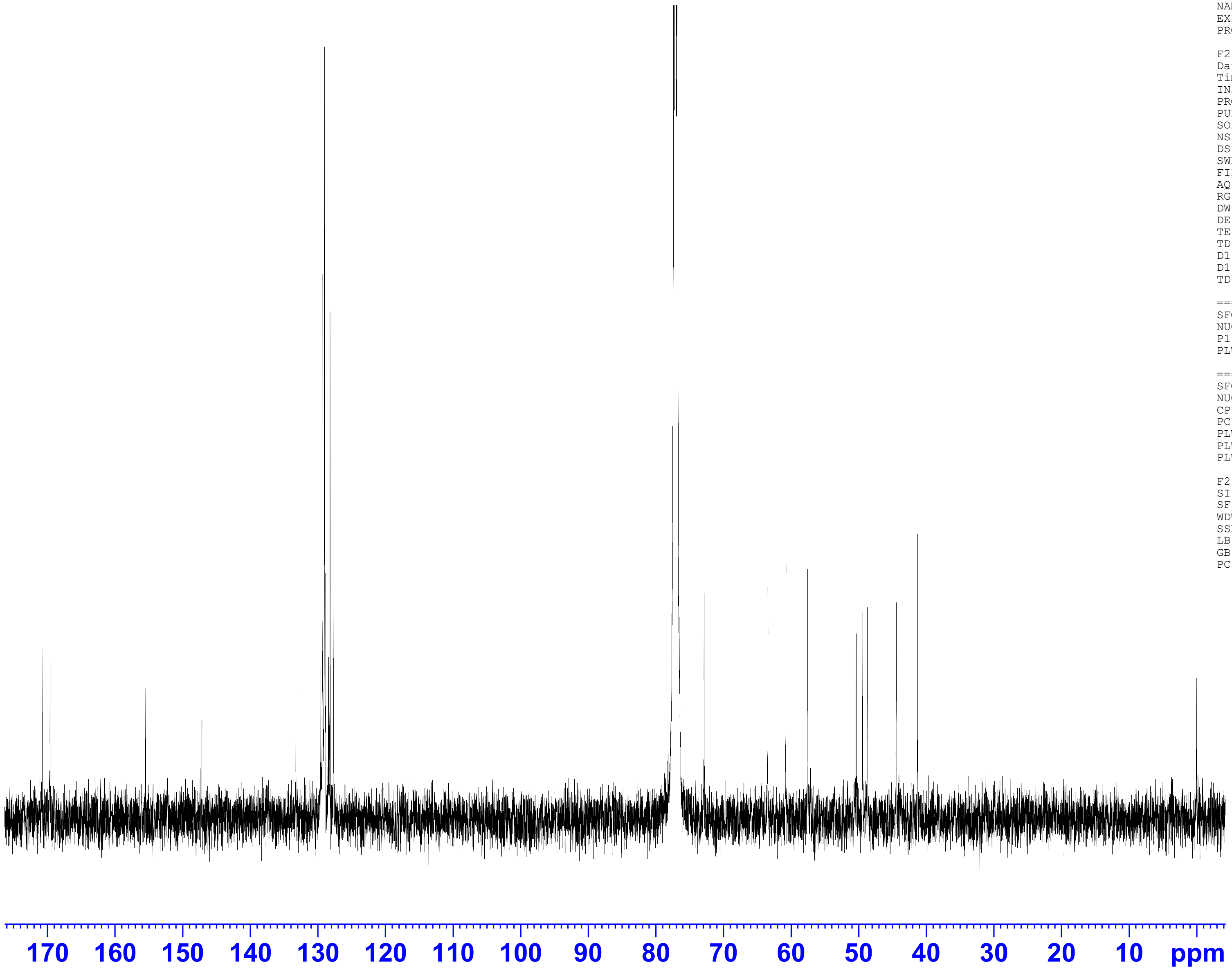

Current Data Parameters
NAME Nov15-2019-SB
EXPNO 21
PROCNO 1

F2 - Acquisition Parameters
Date_ 20191115
Time 13.48
INSTRUM spect
PROBHD 5 mm PABBO BB/
PULPROG zgpg30
SOLVENT CDCl3
NS 3072
DS 4
SWH 29761.904 Hz
FIDRES 0.454131 Hz
AQ 1.1010048 sec
RG 191.93
DW 16.800 usec
DE 6.50 usec
TE 298.0 K
TD 65536
D1 2.00000000 sec
D11 0.03000000 sec
TD0 1

======== CHANNEL f1 ========
SFO1 125.7854522 MHz
NUC1 13C
P1 9.65 usec
PLW1 78.00000000 W

======== CHANNEL f2 ========
SFO2 500.1920008 MHz
NUC2 1H
CPDPRG[2 waltz16
PCPD2 80.00 usec
PLW2 18.75000000 W
PLW12 0.29297000 W
PLW13 0.18750000 W

F2 - Processing parameters
SI 32768
SF 125.7728755 MHz
WDW EM
SSB 0
LB 1.00 Hz
GB 0
PC 1.40

NMR spectra for all additional new compounds

3b

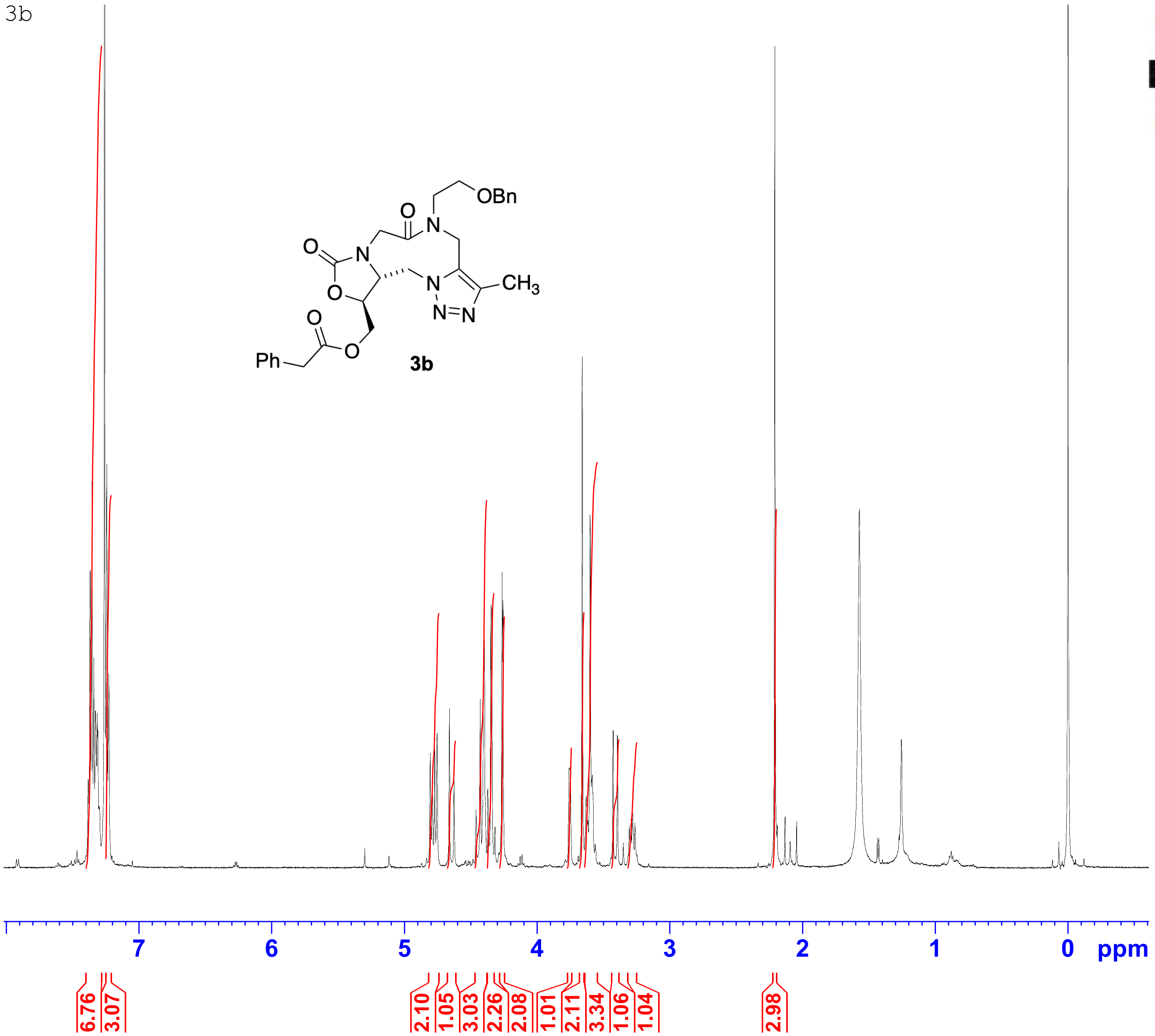


```
Current Data Parameters
NAME       Jul31-2020-SB
EXPNO                 20
PROCNO                 1

F2 - Acquisition Parameters
Date_           20200731
Time               15.09
INSTRUM            spect
PROBHD   5 mm PABBO BB/
PULPROG             zg30
SOLVENT            CDCl3
NS                    16
DS                     2
SWH            10000.000 Hz
FIDRES          0.152588 Hz
AQ             3.2767999 sec
RG                191.93
DW                50.000 usec
DE                  6.50 usec
TE                 298.0 K
TD                 65536
D1            1.00000000 sec
TD0                    1

======== CHANNEL f1 ========
SFO1         500.1930889 MHz
NUC1                  1H
P1                 10.00 usec
PLW1         18.75000000 W

F2 - Processing parameters
SI                 65536
SF           500.1900125 MHz
WDW                   EM
SSB                    0
LB                  0.30 Hz
GB                     0
PC                  1.00
```

3b

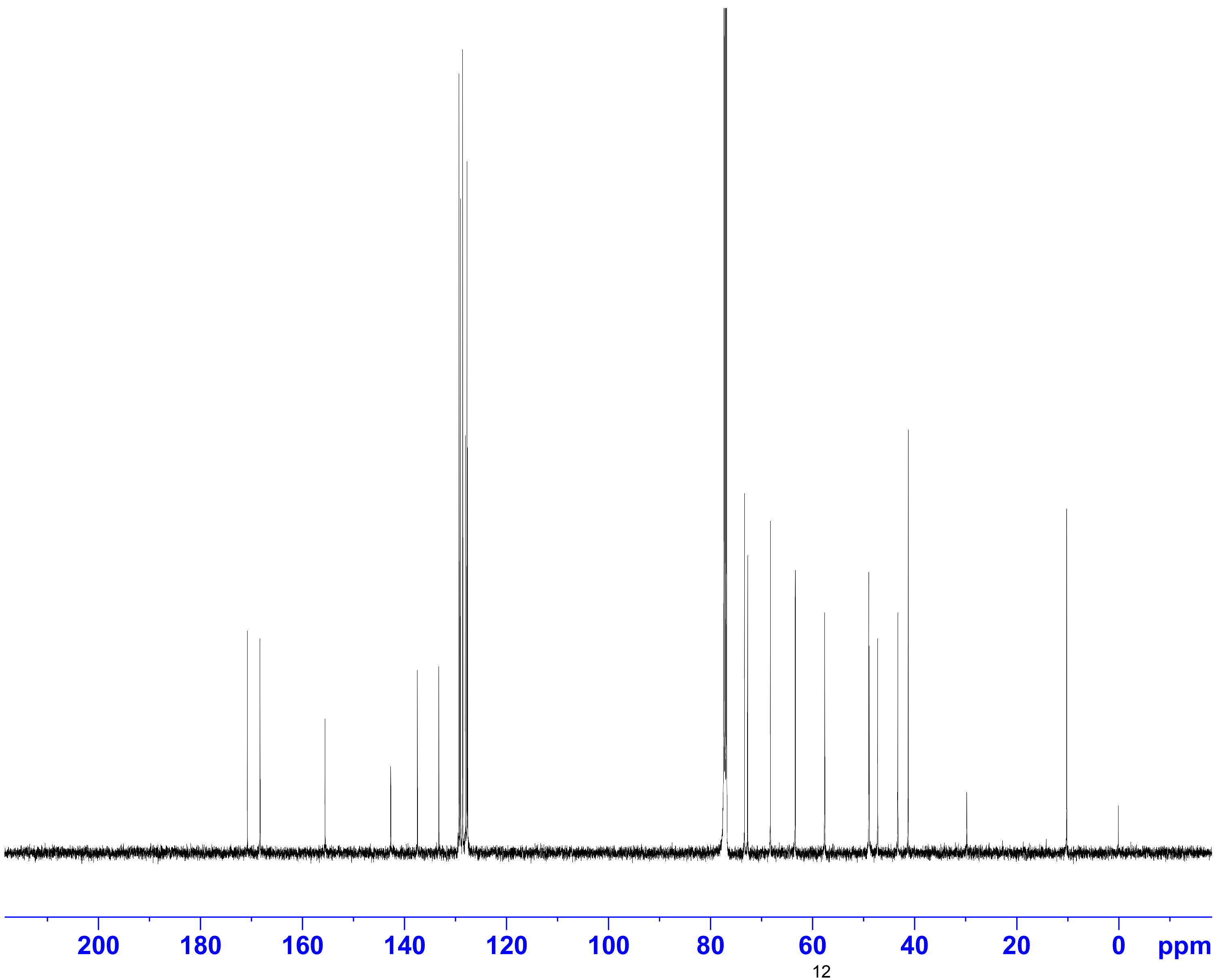


```
Current Data Parameters
NAME       Nov05-2019-SB
EXPNO                 21
PROCNO                 1

F2 - Acquisition Parameters
Date_           20191105
Time               12.23
INSTRUM            spect
PROBHD    5 mm PABBO BB/
PULPROG          zgpg30
SOLVENT            CDCl3
NS                  1536
DS                     4
SWH            29761.904 Hz
FIDRES          0.454131 Hz
AQ             1.1010048 sec
RG                191.93
DW                16.800 usec
DE                  6.50 usec
TE                 298.0 K
TD                 65536
D1            2.00000000 sec
D11           0.03000000 sec
TD0                    1

======== CHANNEL f1 ========
SFO1         125.7854522 MHz
NUC1                 13C
P1                  9.65 usec
PLW1         78.00000000 W

======== CHANNEL f2 ========
SFO2         500.1920008 MHz
NUC2                  1H
CPDPRG[2         waltz16
PCPD2              80.00 usec
PLW2         18.75000000 W
PLW12         0.29297000 W
PLW13         0.18750000 W

F2 - Processing parameters
SI                 32768
SF           125.7728699 MHz
WDW                   EM
SSB                    0
LB                  1.00 Hz
GB                     0
PC                  1.40
```

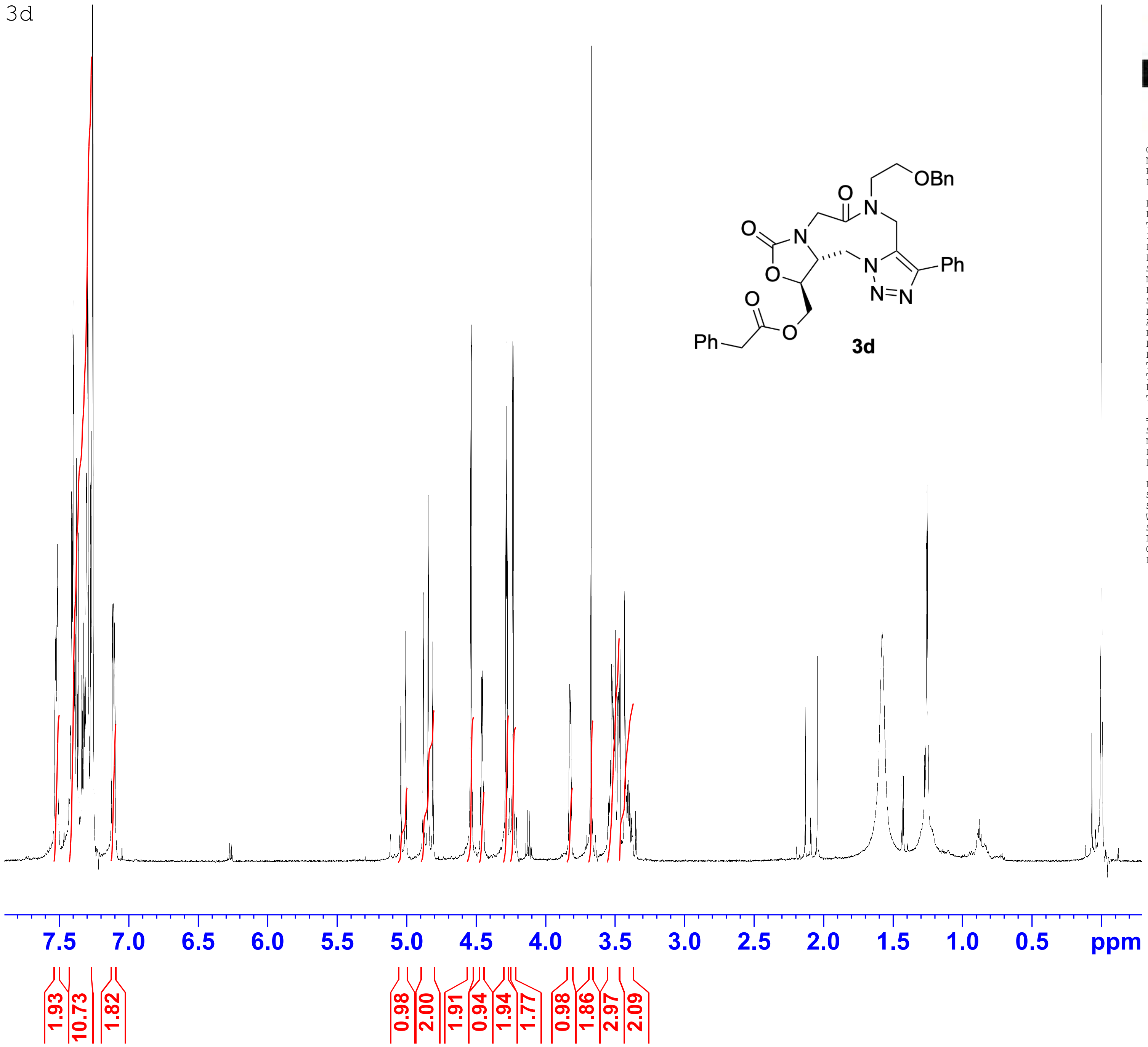


```
Current Data Parameters
NAME        Jul30-2020-SB
EXPNO                  50
PROCNO                  1

F2 - Acquisition Parameters
Date_            20200730
Time                15.12
INSTRUM             spect
PROBHD   5 mm PABBO BB/
PULPROG              zg30
SOLVENT             CDCl3
NS                     16
DS                      2
SWH             10000.000 Hz
FIDRES           0.152588 Hz
AQ              3.2767999 sec
RG                 172.59
DW                 50.000 usec
DE                   6.50 usec
TE                  298.0 K
TD                  65536
D1             1.00000000 sec
TD0                     1

======== CHANNEL f1 ========
SFO1          500.1930889 MHz
NUC1                   1H
P1                  10.00 usec
PLW1          18.75000000 W

F2 - Processing parameters
SI                  65536
SF            500.1900126 MHz
WDW                    EM
SSB                     0
LB                   0.30 Hz
GB                      0
PC                   1.00
```

3d

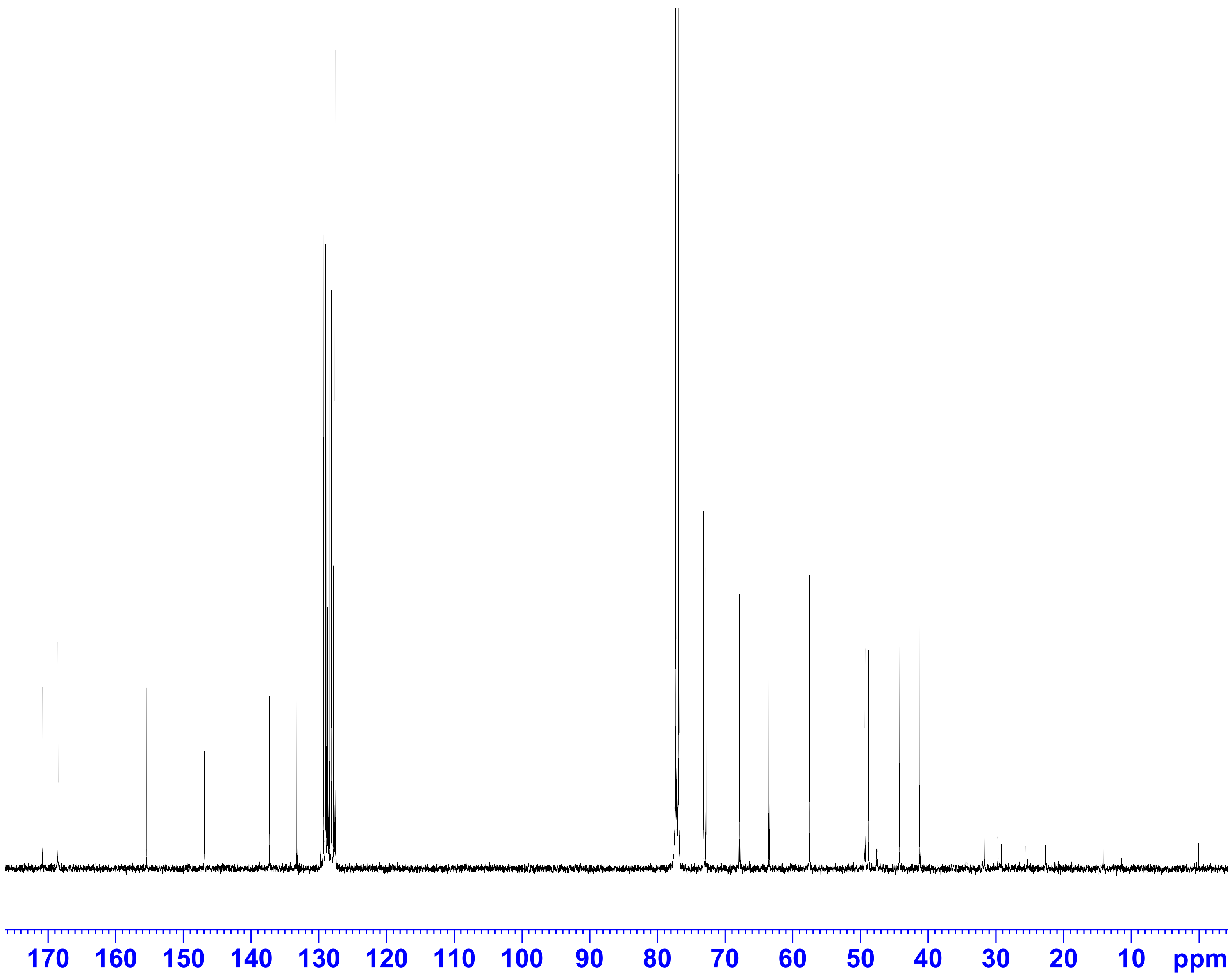

Current Data Parameters
NAME Nov05-2019-SB
EXPNO 41
PROCNO 1

F2 - Acquisition Parameters
Date_ 20191105
Time 13.51
INSTRUM spect
PROBHD 5 mm PABBO BB/
PULPROG zgpg30
SOLVENT CDCl3
NS 1536
DS 4
SWH 29761.904 Hz
FIDRES 0.454131 Hz
AQ 1.1010048 sec
RG 191.93
DW 16.800 usec
DE 6.50 usec
TE 298.0 K
TD 65536
D1 2.00000000 sec
D11 0.03000000 sec
TD0 1

======== CHANNEL f1 ========
SFO1 125.7854522 MHz
NUC1 13C
P1 9.65 usec
PLW1 78.00000000 W

======== CHANNEL f2 ========
SFO2 500.1920008 MHz
NUC2 1H
CPDPRG[2 waltz16
PCPD2 80.00 usec
PLW2 18.75000000 W
PLW12 0.29297000 W
PLW13 0.18750000 W

F2 - Processing parameters
SI 32768
SF 125.7728755 MHz
WDW EM
SSB 0
LB 1.00 Hz
GB 0
PC 1.40

4a

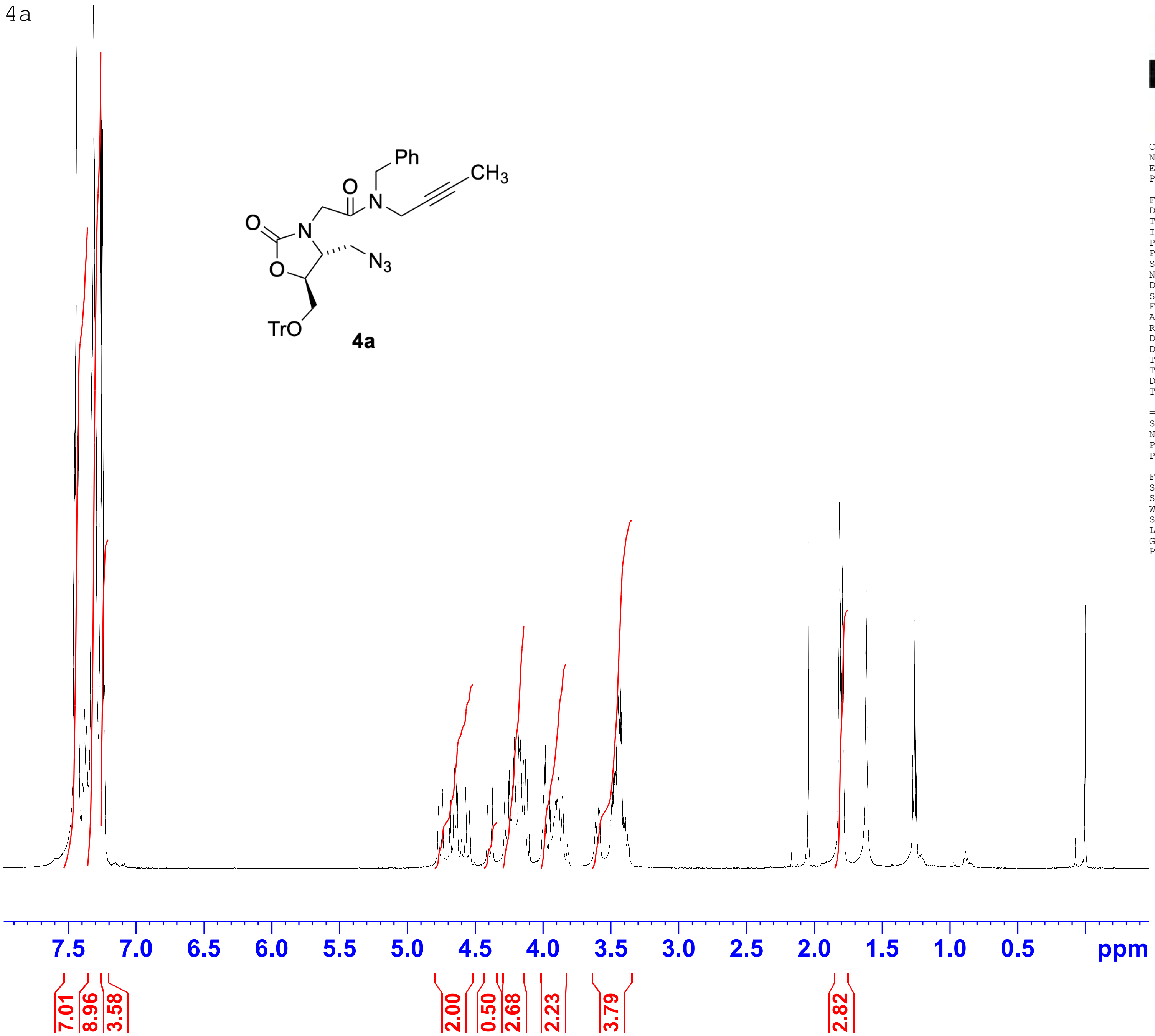


```
Current Data Parameters
NAME       Jun18-2020-SB
EXPNO                 70
PROCNO                 1

F2 - Acquisition Parameters
Date_           20200618
Time               11.56
INSTRUM            spect
PROBHD   5 mm PABBO BB/
PULPROG             zg30
SOLVENT            CDCl3
NS                    16
DS                     2
SWH            10000.000 Hz
FIDRES          0.152588 Hz
AQ             3.2767999 sec
RG                108.06
DW                50.000 usec
DE                  6.50 usec
TE                 298.0 K
TD                 65536
D1            1.00000000 sec
TD0                    1

======== CHANNEL f1 ========
SFO1         500.1930889 MHz
NUC1                  1H
P1                 10.00 usec
PLW1         18.75000000 W

F2 - Processing parameters
SI                 65536
SF           500.1900142 MHz
WDW                   EM
SSB                    0
LB                  0.30 Hz
GB                     0
PC                  1.00
```

4a

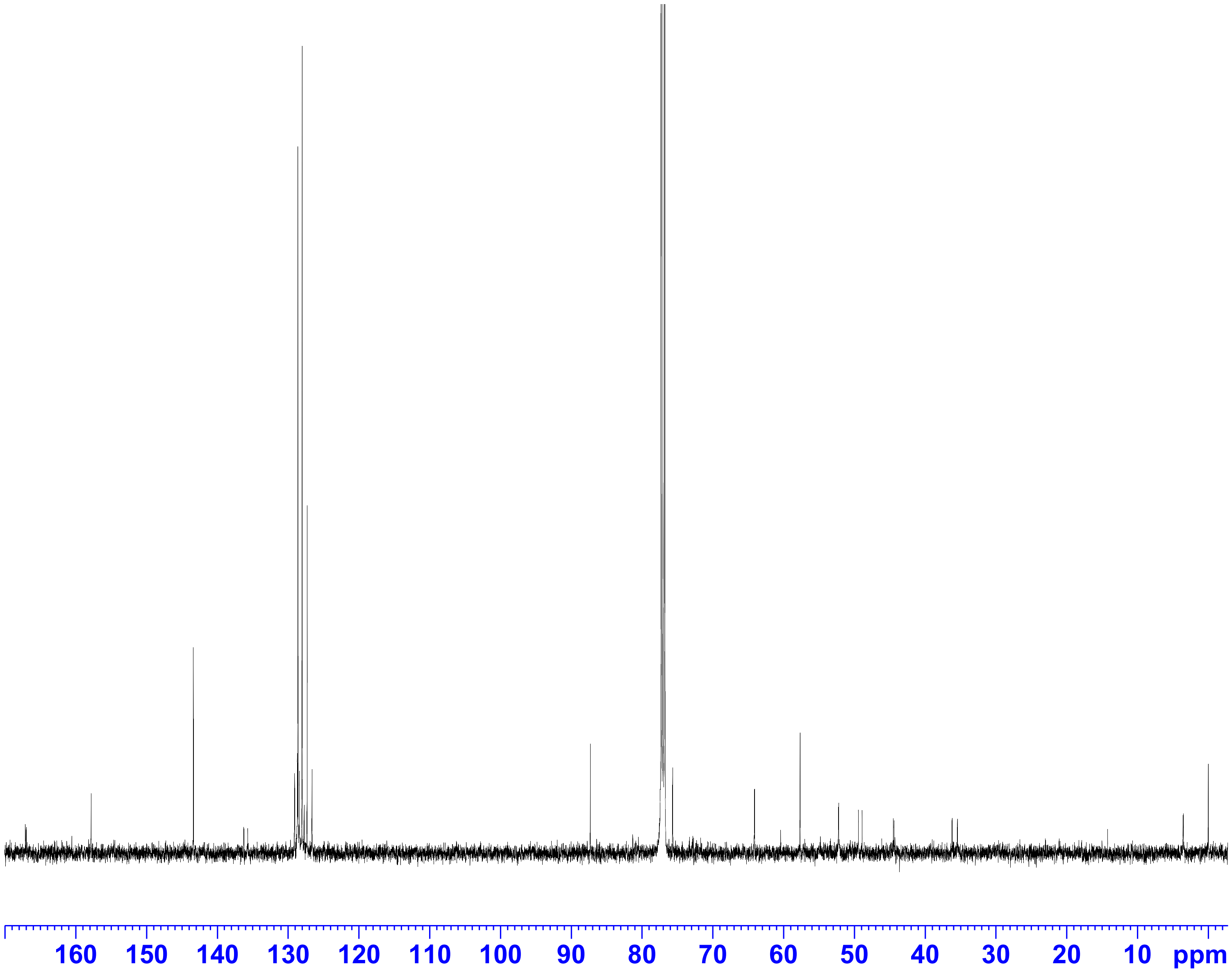


```
Current Data Parameters
NAME       Jul12-2019-SB
EXPNO                 61
PROCNO                 1

F2 - Acquisition Parameters
Date_           20190712
Time               14.51
INSTRUM            spect
PROBHD    5 mm PABBO BB/
PULPROG          zgpg30
SOLVENT            CDCl3
NS                  1024
DS                     4
SWH            29761.904 Hz
FIDRES          0.454131 Hz
AQ             1.1010048 sec
RG                191.93
DW                16.800 usec
DE                  6.50 usec
TE                 298.0 K
TD                 65536
D1            2.00000000 sec
D11           0.03000000 sec
TD0                    1

======== CHANNEL f1 ========
SFO1         125.7854522 MHz
NUC1                 13C
P1                  9.65 usec
PLW1         78.00000000 W

======== CHANNEL f2 ========
SFO2         500.1920008 MHz
NUC2                  1H
CPDPRG[2         waltz16
PCPD2              80.00 usec
PLW2         18.75000000 W
PLW12         0.29297000 W
PLW13         0.18750000 W

F2 - Processing parameters
SI                 32768
SF           125.7728773 MHz
WDW                   EM
SSB                    0
LB                  1.00 Hz
GB                     0
PC                  1.40
```

4b

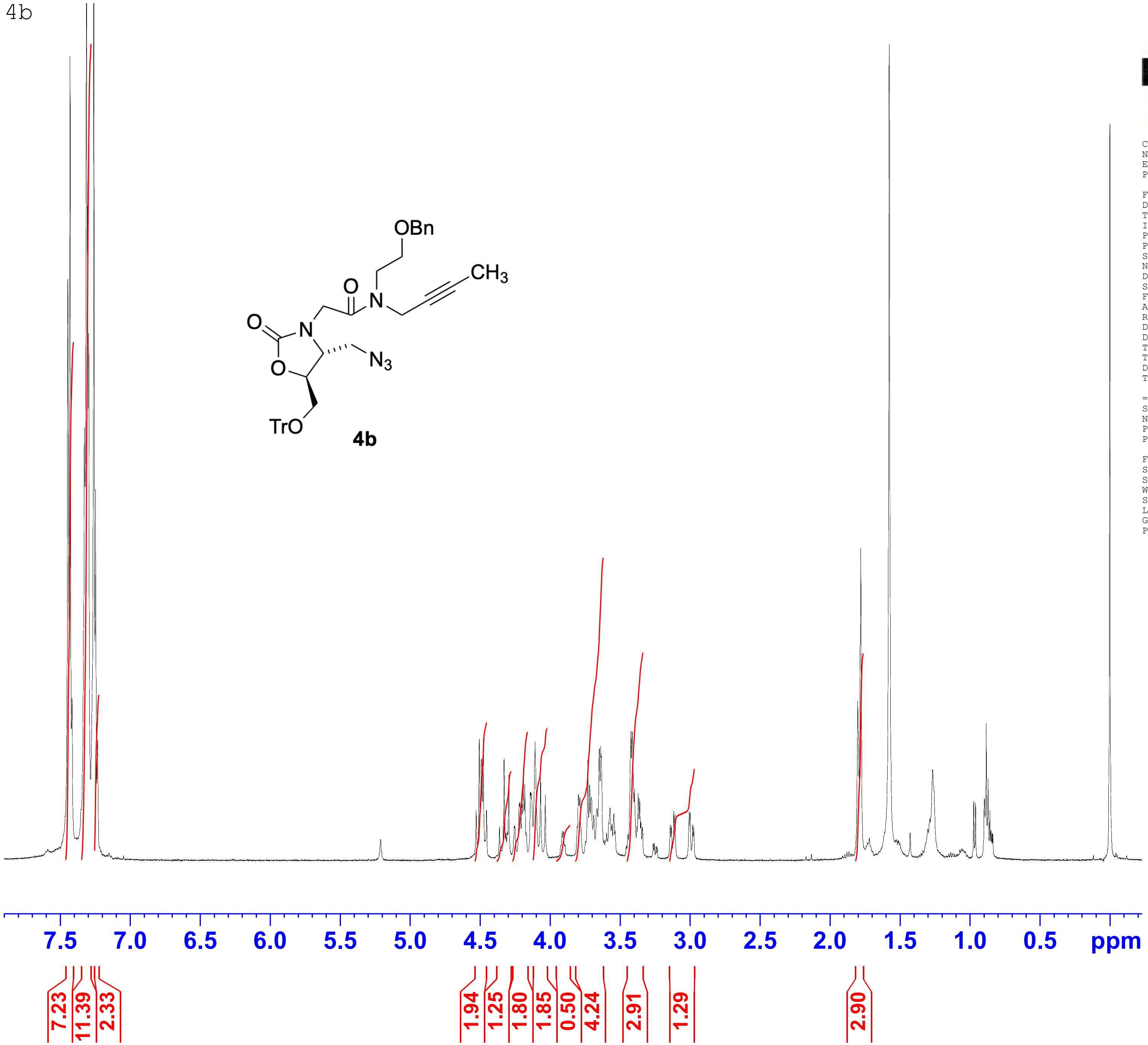


```
Current Data Parameters
NAME       Jun26-2020-SB
EXPNO                 10
PROCNO                 1

F2 - Acquisition Parameters
Date_           20200626
Time               12.22
INSTRUM            spect
PROBHD   5 mm PABBO BB/
PULPROG             zg30
SOLVENT            CDCl3
NS                    16
DS                     2
SWH            10000.000 Hz
FIDRES          0.152588 Hz
AQ             3.2767999 sec
RG                191.93
DW                50.000 usec
DE                  6.50 usec
TE                 298.0 K
TD                 65536
D1            1.00000000 sec
TD0                    1

======== CHANNEL f1 ========
SFO1         500.1930889 MHz
NUC1                  1H
P1                 10.00 usec
PLW1         18.75000000 W

F2 - Processing parameters
SI                 65536
SF           500.1900130 MHz
WDW                   EM
SSB                    0
LB                  0.30 Hz
GB                     0
PC                  1.00
```

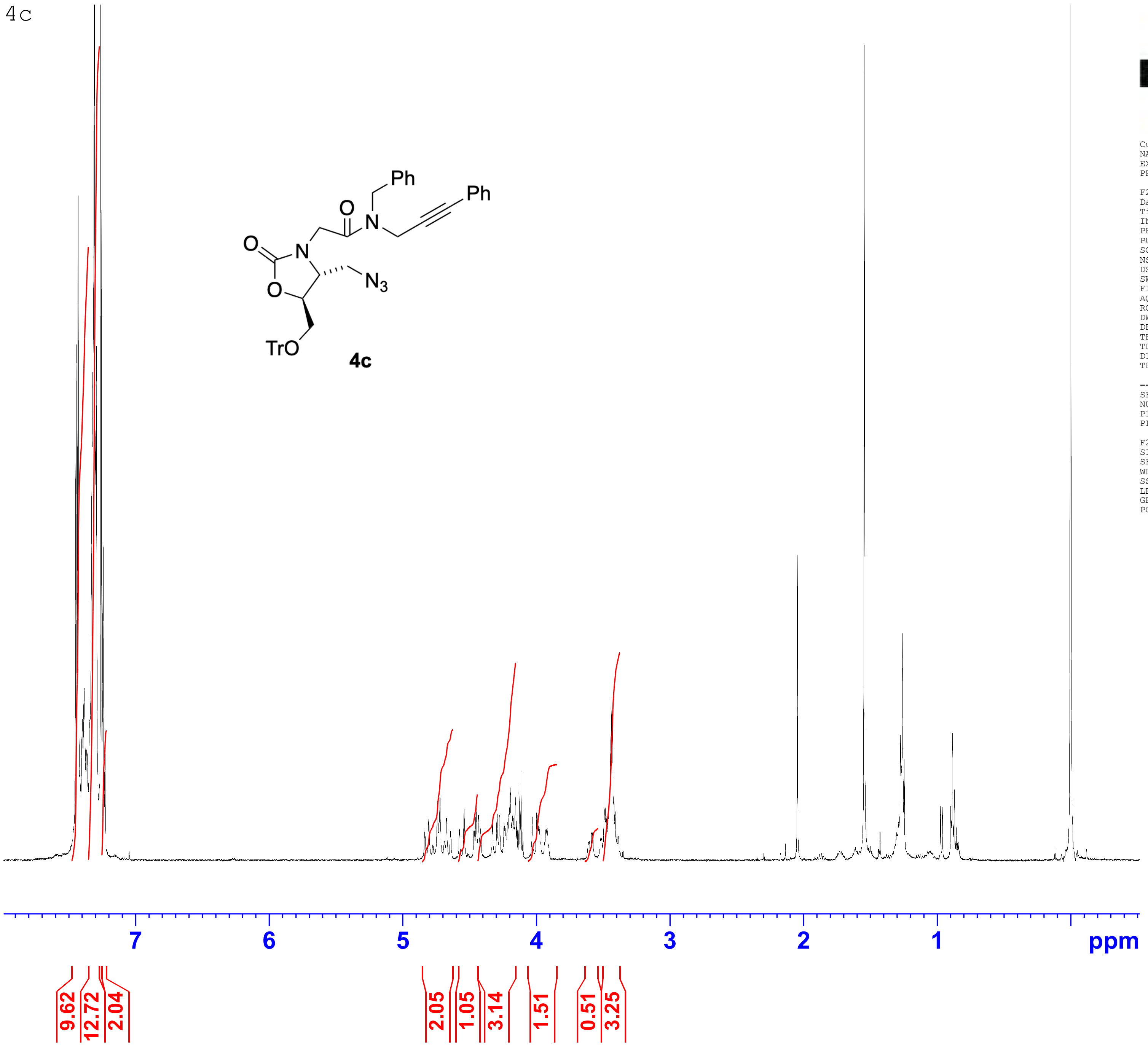


```
Current Data Parameters
NAME        Feb13-2020-SB
EXPNO                  60
PROCNO                  1

F2 - Acquisition Parameters
Date_            20200213
Time                16.17
INSTRUM             spect
PROBHD    5 mm PABBO BB/
PULPROG              zg30
SOLVENT             CDCl3
NS                     16
DS                      2
SWH             10000.000 Hz
FIDRES           0.152588 Hz
AQ              3.2767999 sec
RG                 191.93
DW                 50.000 usec
DE                   6.50 usec
TE                  298.0 K
TD                  65536
D1             1.00000000 sec
TD0                     1

======== CHANNEL f1 ========
SFO1          500.1930889 MHz
NUC1                   1H
P1                  10.00 usec
PLW1          18.75000000 W

F2 - Processing parameters
SI                  65536
SF            500.1900128 MHz
WDW                    EM
SSB                     0
LB                   0.30 Hz
GB                      0
PC                   1.00
```

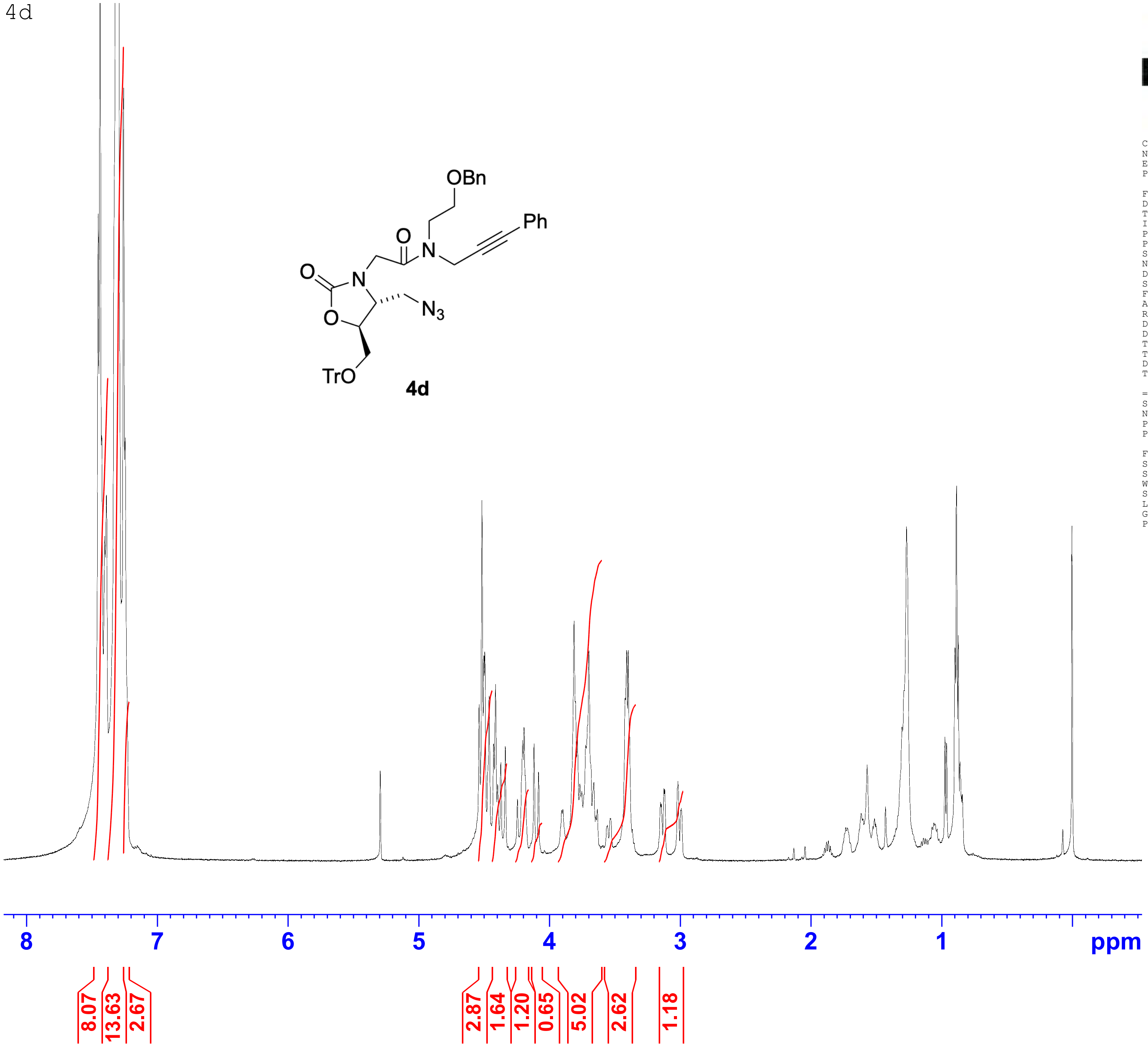


```
Current Data Parameters
NAME       Feb28-2020-SB
EXPNO                 50
PROCNO                 1

F2 - Acquisition Parameters
Date_           20200228
Time               14.52
INSTRUM            spect
PROBHD   5 mm PABBO BB/
PULPROG             zg30
SOLVENT            CDCl3
NS                    16
DS                     2
SWH          10000.000 Hz
FIDRES        0.152588 Hz
AQ           3.2767999 sec
RG              151.74
DW              50.000 usec
DE                6.50 usec
TE               298.0 K
TD                65536
D1          1.00000000 sec
TD0                    1

======== CHANNEL f1 ========
SFO1       500.1930889 MHz
NUC1                  1H
P1               10.00 usec
PLW1       18.75000000 W

F2 - Processing parameters
SI                 65536
SF         500.1900144 MHz
WDW                   EM
SSB                    0
LB                  0.30 Hz
GB                     0
PC                  1.00
```

9a

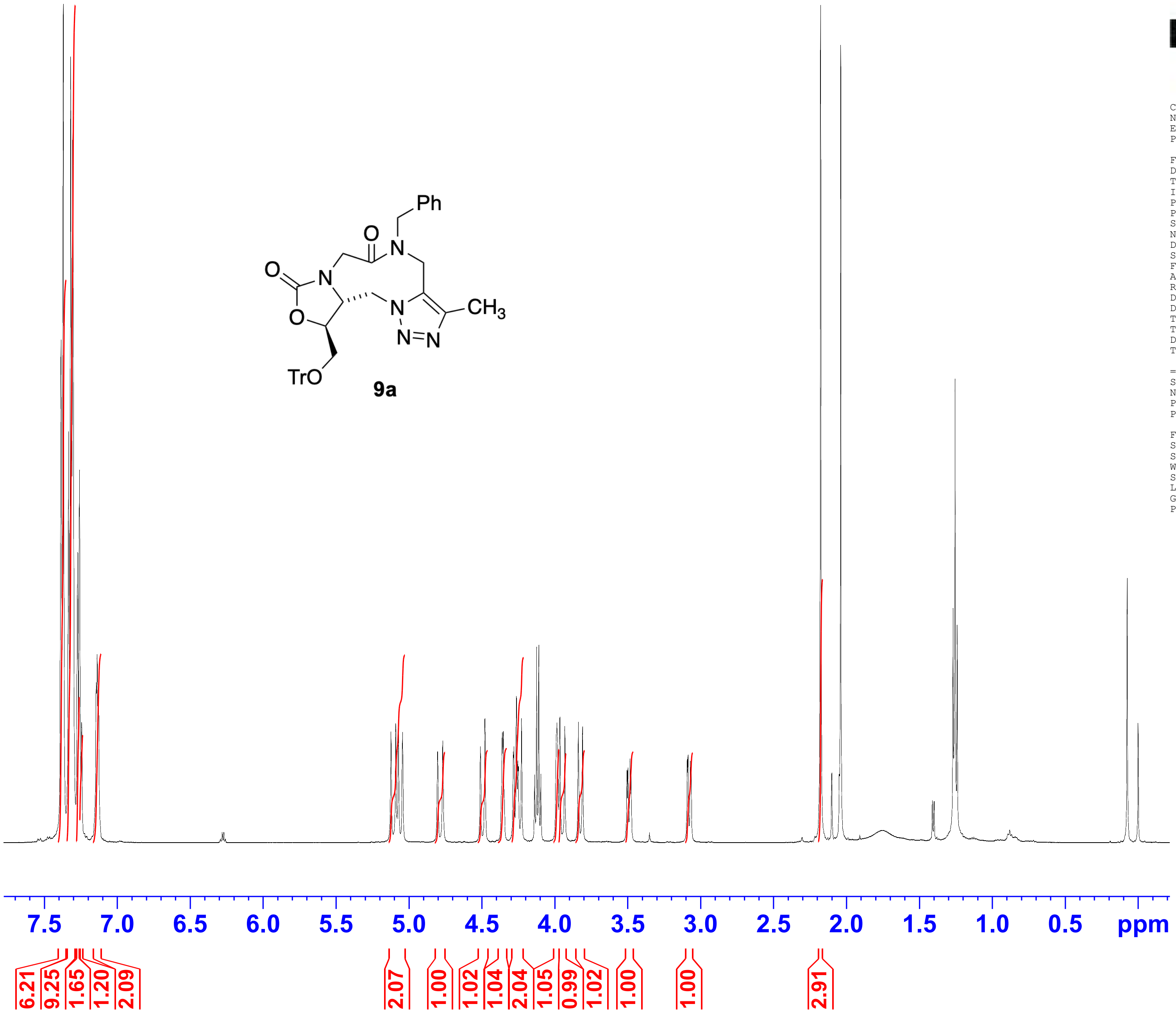


```
Current Data Parameters
NAME        Jul10-2020-SB
EXPNO                  80
PROCNO                  1

F2 - Acquisition Parameters
Date_            20200710
Time                15.17
INSTRUM             spect
PROBHD   5 mm PABBO BB/
PULPROG              zg30
SOLVENT             CDCl3
NS                     16
DS                      2
SWH             10000.000 Hz
FIDRES           0.152588 Hz
AQ              3.2767999 sec
RG                  70.72
DW                 50.000 usec
DE                   6.50 usec
TE                  298.2 K
TD                  65536
D1             1.00000000 sec
TD0                     1

======== CHANNEL f1 ========
SFO1          500.1930889 MHz
NUC1                   1H
P1                  10.00 usec
PLW1          18.75000000 W

F2 - Processing parameters
SI                  65536
SF            500.1900112 MHz
WDW                    EM
SSB                     0
LB                   0.30 Hz
GB                      0
PC                   1.00
```

9a

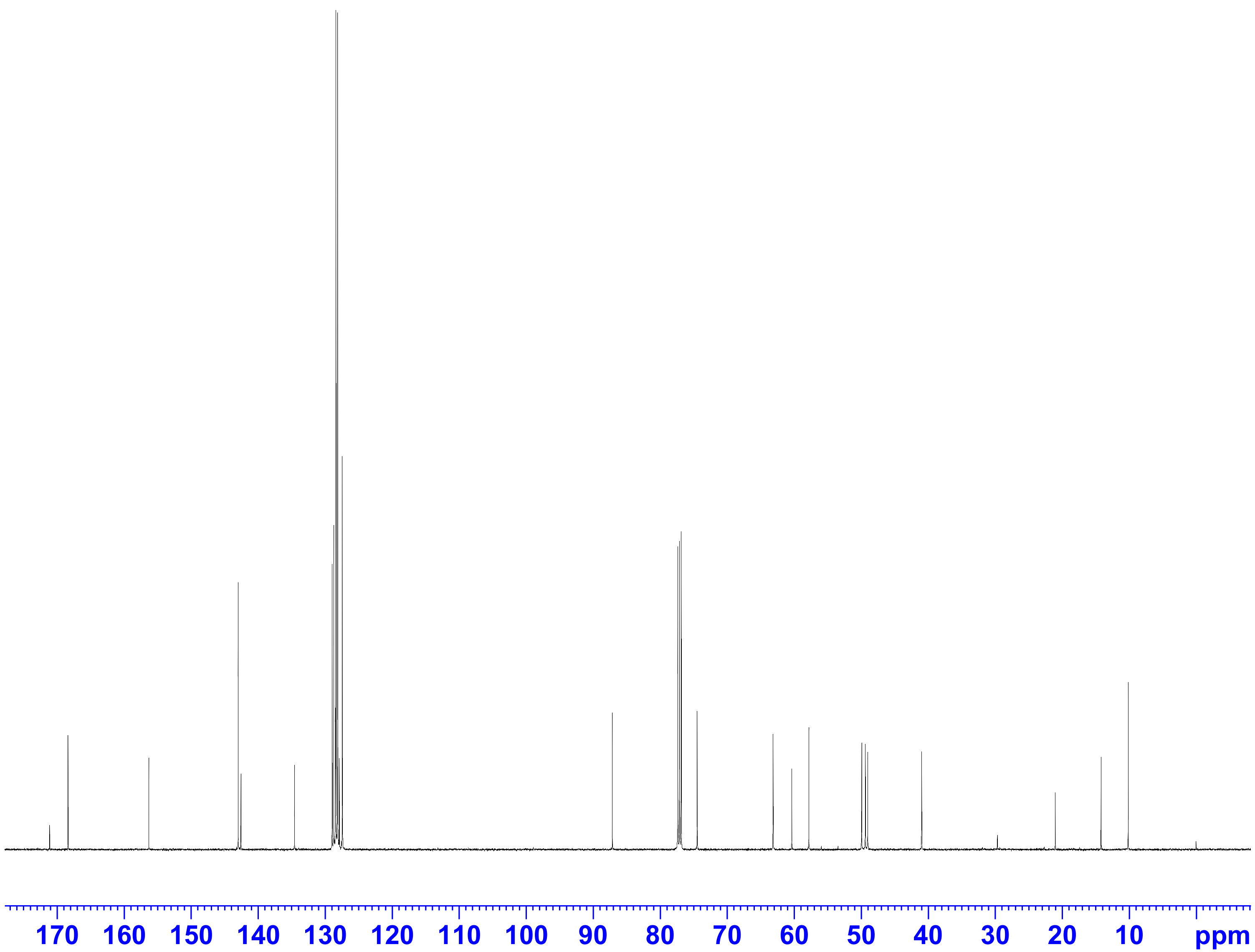


```
Current Data Parameters
NAME       Jun11-2018-SB
EXPNO                 10
PROCNO                 1

F2 - Acquisition Parameters
Date_           20180611
Time               13.24
INSTRUM            spect
PROBHD     5 mm PABBO BB/
PULPROG          zgpg30
SOLVENT            CDCl3
NS                  1024
DS                     4
SWH            29761.904 Hz
FIDRES          0.454131 Hz
AQ             1.1010048 sec
RG                191.93
DW                16.800 usec
DE                  6.50 usec
TE                 298.0 K
TD                 65536
D1            2.00000000 sec
D11           0.03000000 sec
TD0                    1

======== CHANNEL f1 ========
SFO1         125.7854522 MHz
NUC1                 13C
P1                  9.65 usec
PLW1         78.00000000 W

======== CHANNEL f2 ========
SFO2         500.1920008 MHz
NUC2                  1H
CPDPRG[2         waltz16
PCPD2              80.00 usec
PLW2         18.75000000 W
PLW12         0.29297000 W
PLW13         0.18750000 W

F2 - Processing parameters
SI                 32768
SF           125.7728811 MHz
WDW                   EM
SSB                    0
LB                  1.00 Hz
GB                     0
PC                  1.40
```

9b

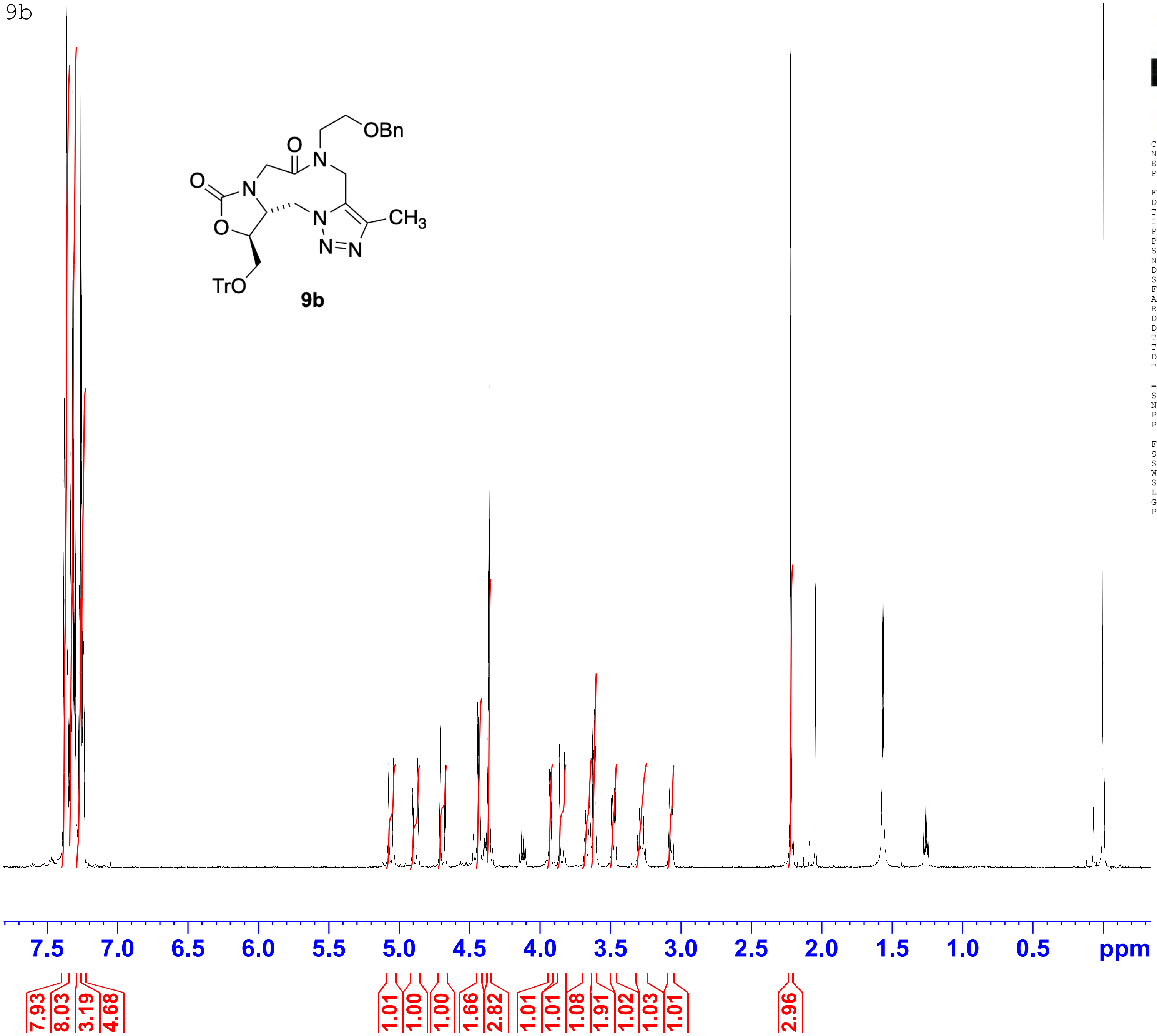

BRUKER

Current Data Parameters
NAME Jul12-2020-SB
EXPNO 80
PROCNO 1

F2 - Acquisition Parameters
Date_ 20200712
Time 14.59
INSTRUM spect
PROBHD 5 mm PABBO BB/
PULPROG zg30
SOLVENT CDCl3
NS 16
DS 2
SWH 10000.000 Hz
FIDRES 0.152588 Hz
AQ 3.2767999 sec
RG 191.93
DW 50.000 usec
DE 6.50 usec
TE 298.2 K
TD 65536
D1 1.00000000 sec
TD0 1

======== CHANNEL f1 ========
SFO1 500.1930889 MHz
NUC1 1H
P1 10.00 usec
PLW1 18.75000000 W

F2 - Processing parameters
SI 65536
SF 500.1900122 MHz
WDW EM
SSB 0
LB 0.30 Hz
GB 0
PC 1.00

9b

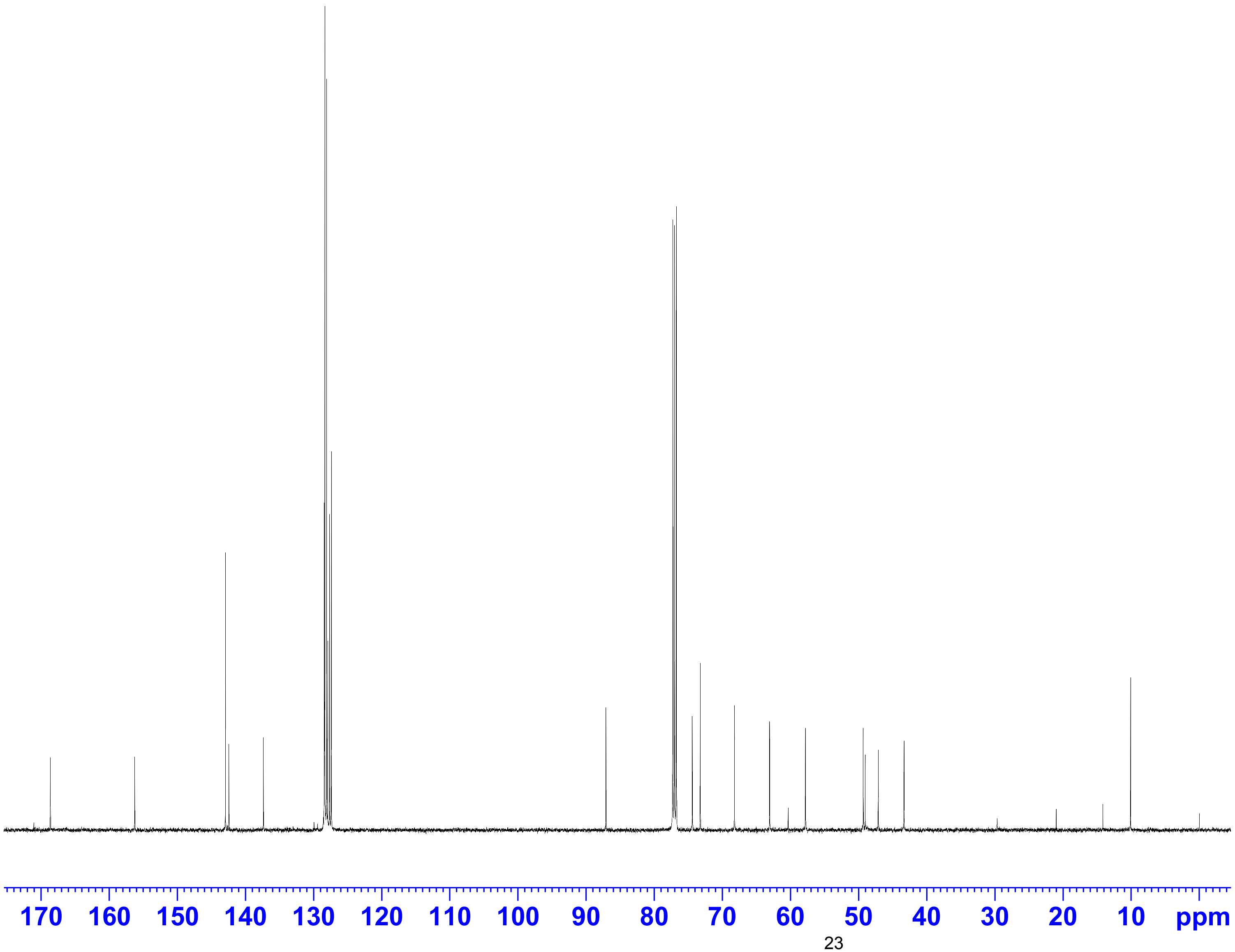


```
Current Data Parameters
NAME       Sep25-2019-SB
EXPNO                 11
PROCNO                 1

F2 - Acquisition Parameters
Date_           20190925
Time               11.57
INSTRUM            spect
PROBHD   5 mm PABBO BB/
PULPROG          zgpg30
SOLVENT            CDCl3
NS                  1024
DS                     4
SWH            29761.904 Hz
FIDRES          0.454131 Hz
AQ             1.1010048 sec
RG                191.93
DW                16.800 usec
DE                  6.50 usec
TE                 298.0 K
TD                 65536
D1            2.00000000 sec
D11           0.03000000 sec
TD0                    1

======== CHANNEL f1 ========
SFO1         125.7854522 MHz
NUC1                 13C
P1                  9.65 usec
PLW1         78.00000000 W

======== CHANNEL f2 ========
SFO2         500.1920008 MHz
NUC2                  1H
CPDPRG[2         waltz16
PCPD2              80.00 usec
PLW2         18.75000000 W
PLW12         0.29297000 W
PLW13         0.18750000 W

F2 - Processing parameters
SI                 32768
SF           125.7728800 MHz
WDW                   EM
SSB                    0
LB                  1.00 Hz
GB                     0
PC                  1.40
```

9c

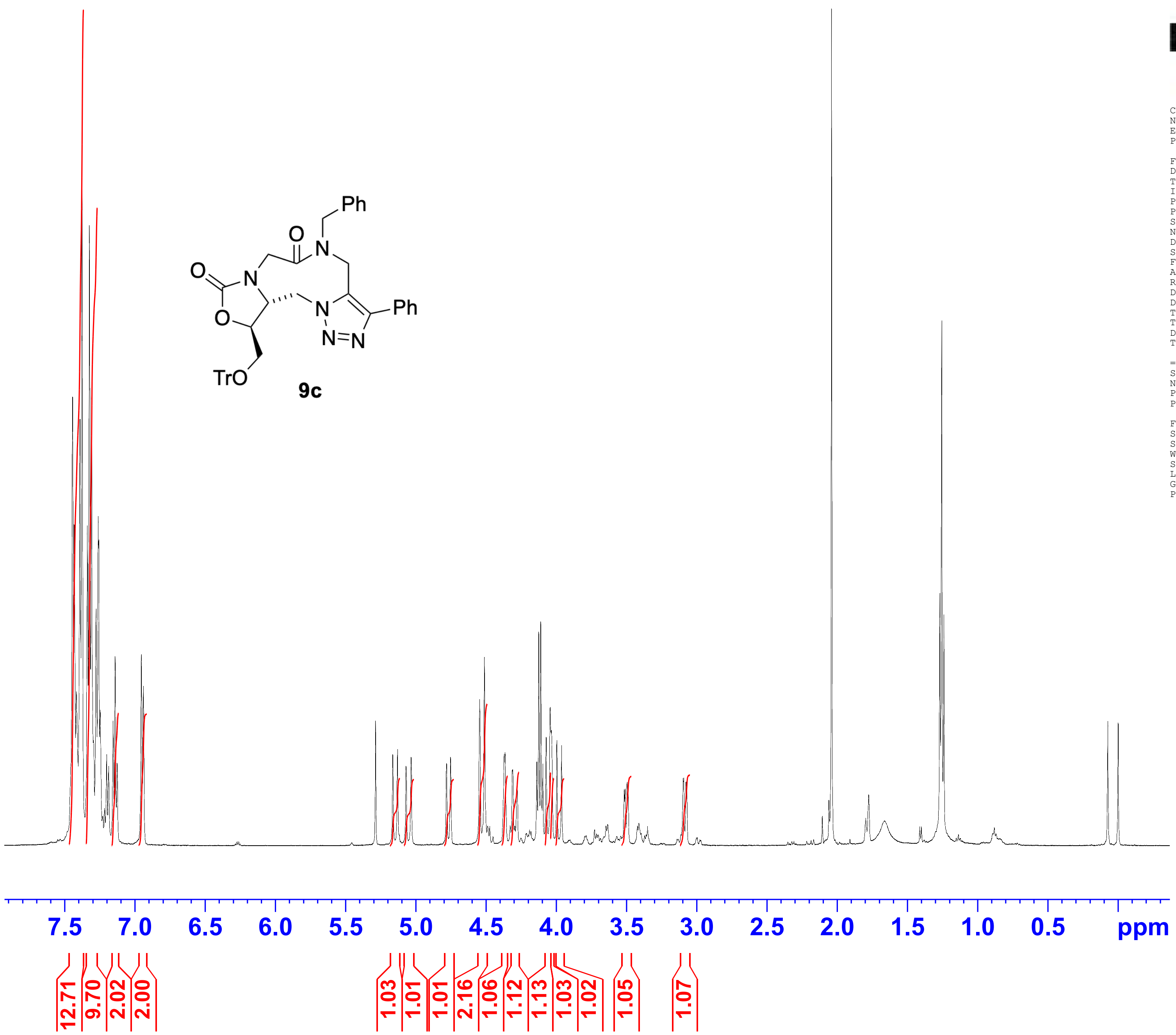

Current Data Parameters
NAME Jun07-2020-SB
EXPNO 10
PROCNO 1

F2 - Acquisition Parameters
Date_ 20200607
Time 15.41
INSTRUM spect
PROBHD 5 mm PABBO BB/
PULPROG zg30
SOLVENT CDCl3
NS 16
DS 2
SWH 10000.000 Hz
FIDRES 0.152588 Hz
AQ 3.2767999 sec
RG 108.06
DW 50.000 usec
DE 6.50 usec
TE 298.0 K
TD 65536
D1 1.00000000 sec
TD0 1

======== CHANNEL f1 ========
SFO1 500.1930889 MHz
NUC1 1H
P1 10.00 usec
PLW1 18.75000000 W

F2 - Processing parameters
SI 65536
SF 500.1900136 MHz
WDW EM
SSB 0
LB 0.30 Hz
GB 0
PC 1.00

9c

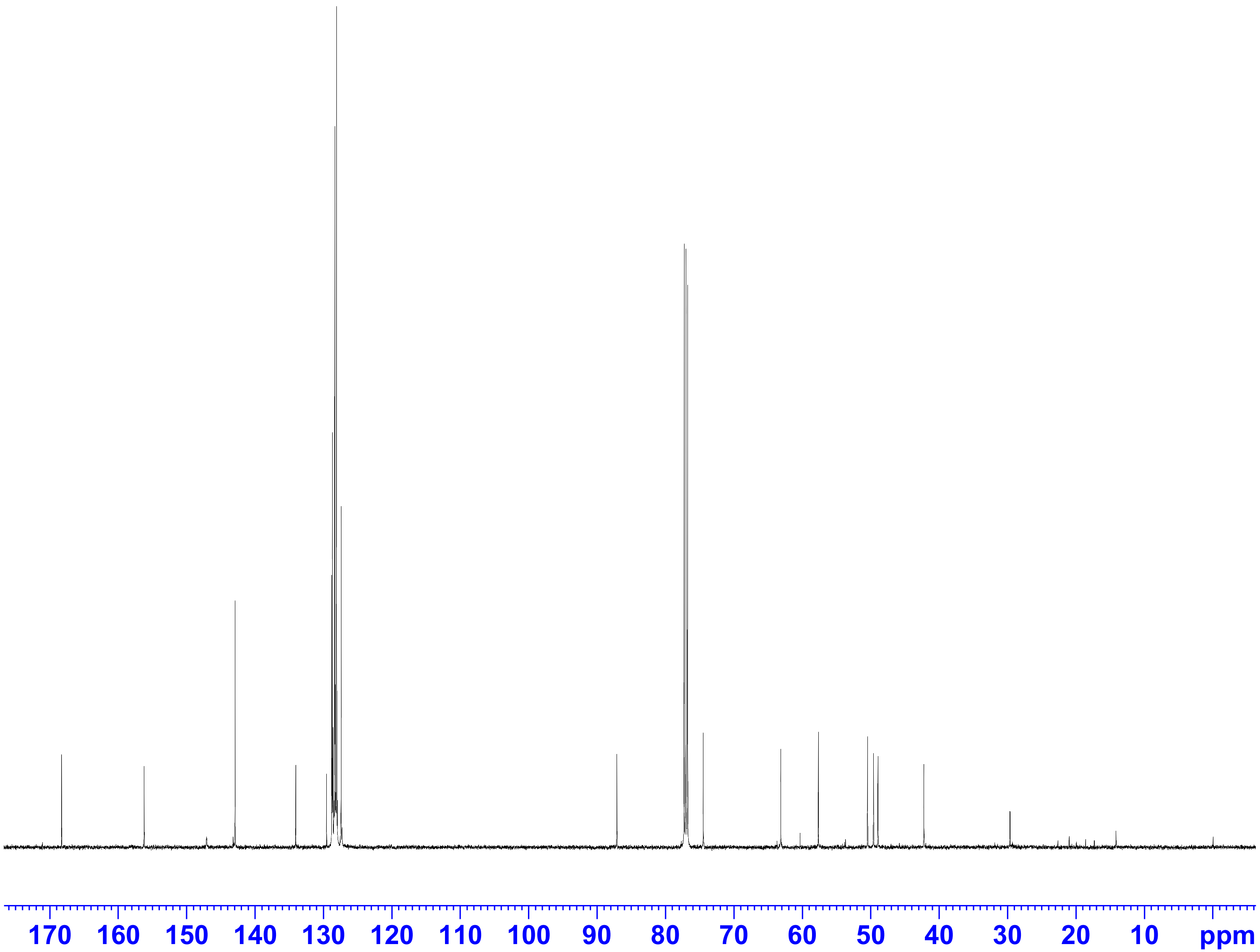

Current Data Parameters
NAME Jul18-2019-SB
EXPNO 31
PROCNO 1

F2 - Acquisition Parameters
Date_ 20190718
Time 11.53
INSTRUM spect
PROBHD 5 mm PABBO BB/
PULPROG zgpg30
SOLVENT CDCl3
NS 1024
DS 4
SWH 29761.904 Hz
FIDRES 0.454131 Hz
AQ 1.1010048 sec
RG 191.93
DW 16.800 usec
DE 6.50 usec
TE 298.0 K
TD 65536
D1 2.00000000 sec
D11 0.03000000 sec
TD0 1

======== CHANNEL f1 ========
SFO1 125.7854522 MHz
NUC1 13C
P1 9.65 usec
PLW1 78.00000000 W

======== CHANNEL f2 ========
SFO2 500.1920008 MHz
NUC2 1H
CPDPRG[2 waltz16
PCPD2 80.00 usec
PLW2 18.75000000 W
PLW12 0.29297000 W
PLW13 0.18750000 W

F2 - Processing parameters
SI 32768
SF 125.7728895 MHz
WDW EM
SSB 0
LB 1.00 Hz
GB 0
PC 1.40

9d

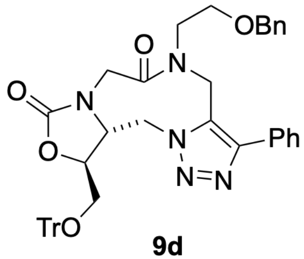

9d

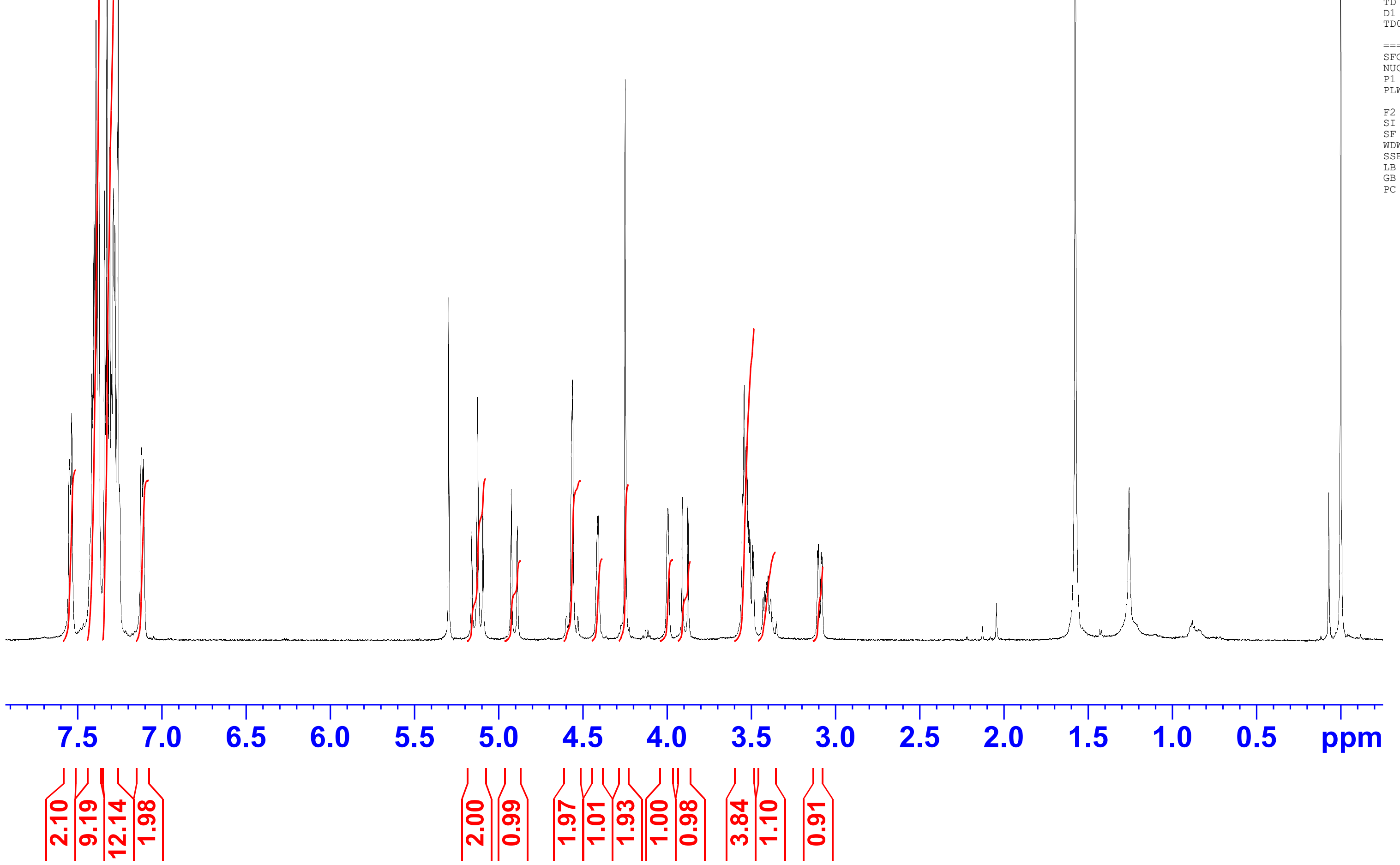


```
Current Data Parameters
NAME       Jun07-2020-SB
EXPNO                 20
PROCNO                 1

F2 - Acquisition Parameters
Date_           20200607
Time               15.45
INSTRUM            spect
PROBHD   5 mm PABBO BB/
PULPROG             zg30
SOLVENT            CDCl3
NS                    16
DS                     2
SWH            10000.000 Hz
FIDRES          0.152588 Hz
AQ             3.2767999 sec
RG                191.93
DW                50.000 usec
DE                  6.50 usec
TE                 298.0 K
TD                 65536
D1            1.00000000 sec
TD0                    1

======== CHANNEL f1 ========
SFO1         500.1930889 MHz
NUC1                  1H
P1                 10.00 usec
PLW1         18.75000000 W

F2 - Processing parameters
SI                 65536
SF           500.1900128 MHz
WDW                   EM
SSB                    0
LB                  0.30 Hz
GB                     0
PC                  1.00
```

9c

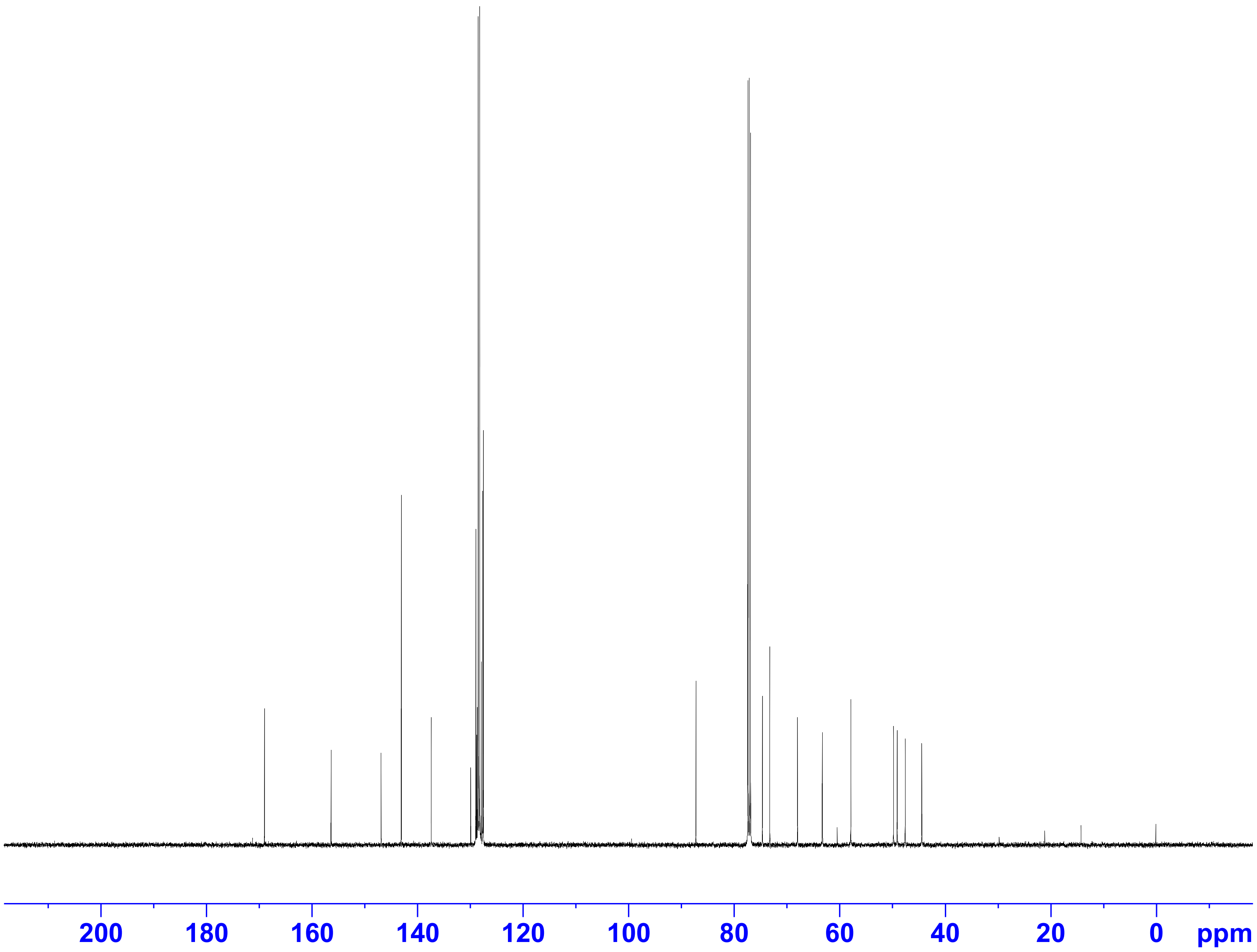


```
Current Data Parameters
NAME        Oct31-2019-SB
EXPNO                  11
PROCNO                  1

F2 - Acquisition Parameters
Date_            20191031
Time                12.57
INSTRUM             spect
PROBHD   5 mm PABBO BB/
PULPROG           zgpg30
SOLVENT            CDCl3
NS                   1024
DS                      4
SWH             29761.904 Hz
FIDRES           0.454131 Hz
AQ              1.1010048 sec
RG                 191.93
DW                 16.800 usec
DE                   6.50 usec
TE                  298.0 K
TD                  65536
D1             2.00000000 sec
D11            0.03000000 sec
TD0                     1

======== CHANNEL f1 ========
SFO1          125.7854522 MHz
NUC1                  13C
P1                   9.65 usec
PLW1          78.00000000 W

======== CHANNEL f2 ========
SFO2          500.1920008 MHz
NUC2                   1H
CPDPRG[2          waltz16
PCPD2               80.00 usec
PLW2          18.75000000 W
PLW12          0.29297000 W
PLW13          0.18750000 W

F2 - Processing parameters
SI                  32768
SF            125.7728696 MHz
WDW                    EM
SSB                     0
LB                   1.00 Hz
GB                      0
PC                   1.40
```

6a

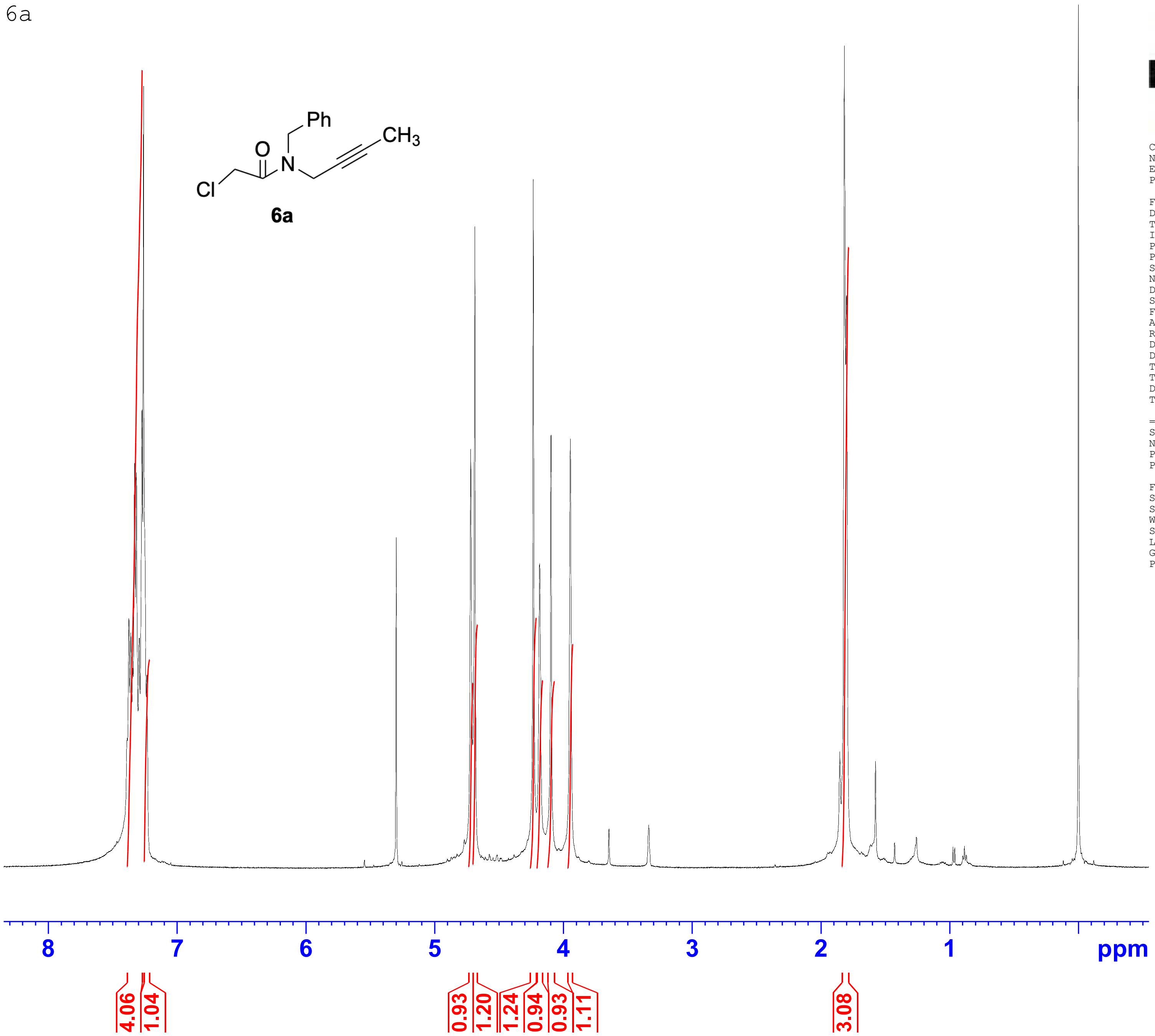


```
Current Data Parameters
NAME       Feb06-2020-SB
EXPNO                 50
PROCNO                 1

F2 - Acquisition Parameters
Date_           20200206
Time               15.16
INSTRUM            spect
PROBHD   5 mm PABBO BB/
PULPROG             zg30
SOLVENT            CDCl3
NS                    16
DS                     2
SWH            10000.000 Hz
FIDRES          0.152588 Hz
AQ             3.2767999 sec
RG                137.92
DW                50.000 usec
DE                  6.50 usec
TE                 298.2 K
TD                 65536
D1            1.00000000 sec
TD0                    1

======== CHANNEL f1 ========
SFO1         500.1930889 MHz
NUC1                  1H
P1                 10.00 usec
PLW1         18.75000000 W

F2 - Processing parameters
SI                 65536
SF           500.1900122 MHz
WDW                   EM
SSB                    0
LB                  0.30 Hz
GB                     0
PC                  1.00
```

6b

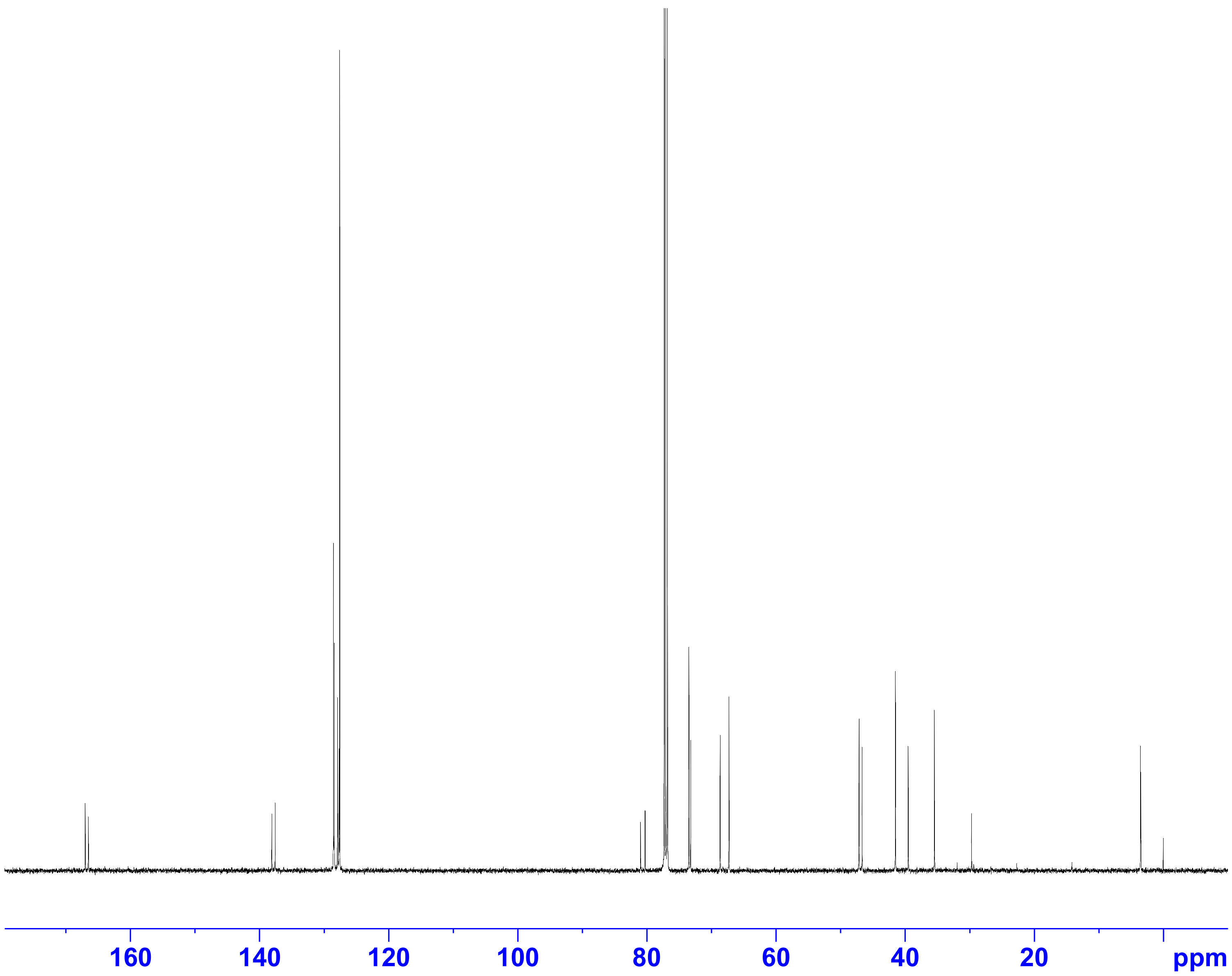


```
Current Data Parameters
NAME       Nov12-2019-SB
EXPNO                 11
PROCNO                 1

F2 - Acquisition Parameters
Date_           20191112
Time               13.54
INSTRUM            spect
PROBHD    5 mm PABBO BB/
PULPROG          zgpg30
SOLVENT            CDCl3
NS                  2048
DS                     4
SWH            29761.904 Hz
FIDRES          0.454131 Hz
AQ             1.1010048 sec
RG                191.93
DW                16.800 usec
DE                  6.50 usec
TE                 298.0 K
TD                 65536
D1            2.00000000 sec
D11           0.03000000 sec
TD0                    1

======== CHANNEL f1 ========
SFO1         125.7854522 MHz
NUC1                 13C
P1                  9.65 usec
PLW1         78.00000000 W

======== CHANNEL f2 ========
SFO2         500.1920008 MHz
NUC2                  1H
CPDPRG[2         waltz16
PCPD2              80.00 usec
PLW2         18.75000000 W
PLW12         0.29297000 W
PLW13         0.18750000 W

F2 - Processing parameters
SI                 32768
SF           125.7728774 MHz
WDW                   EM
SSB                    0
LB                  1.00 Hz
GB                     0
PC                  1.40
```

6b

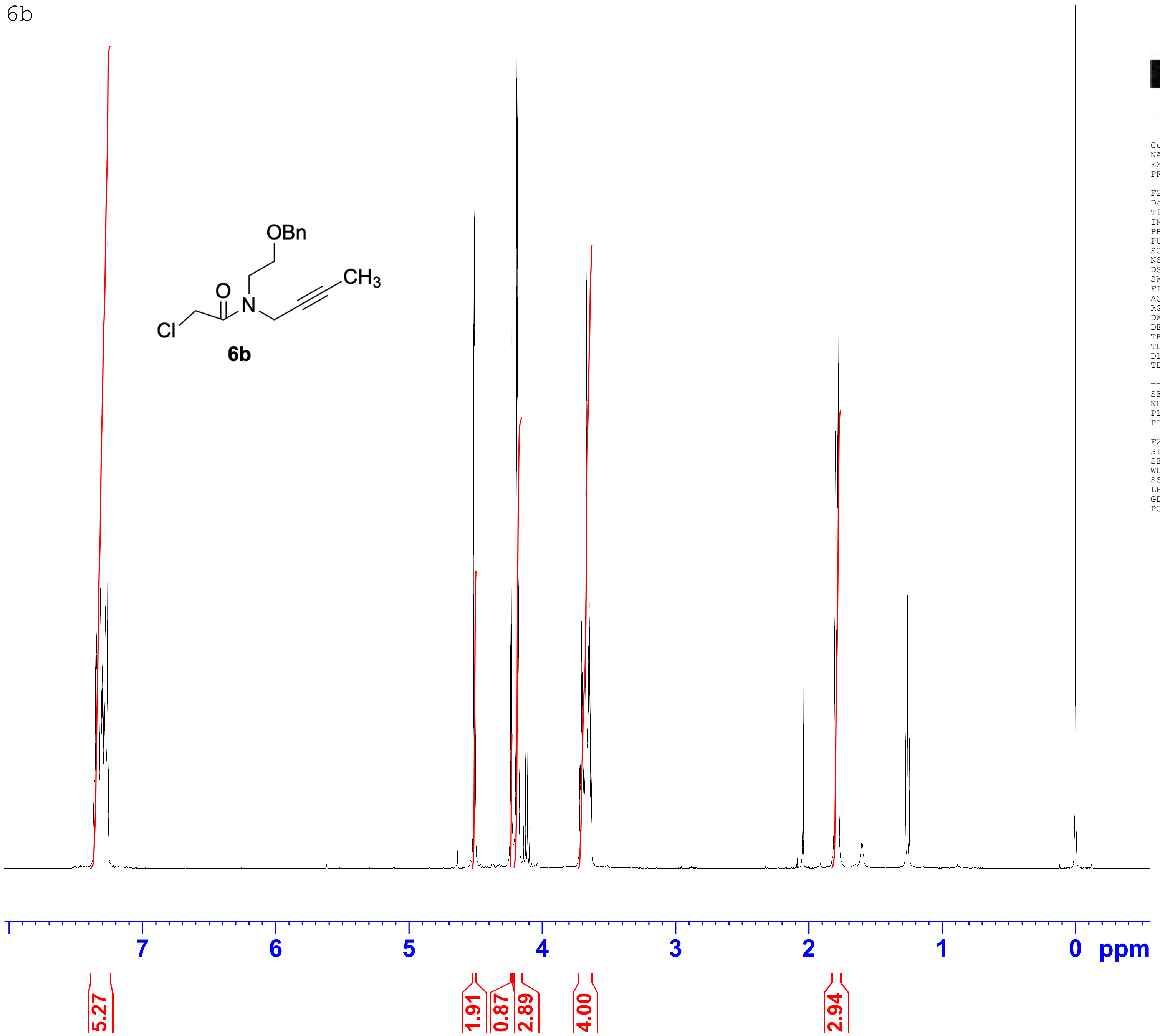

Current Data Parameters
NAME Jan24-2020-SB
EXPNO 20
PROCNO 1

F2 - Acquisition Parameters
Date_ 20200124
Time 15.00
INSTRUM spect
PROBHD 5 mm PABBO BB/
PULPROG zg30
SOLVENT CDCl3
NS 16
DS 2
SWH 10000.000 Hz
FIDRES 0.152588 Hz
AQ 3.2767999 sec
RG 122.2
DW 50.000 usec
DE 6.50 usec
TE 298.0 K
TD 65536
D1 1.00000000 sec
TD0 1

======== CHANNEL f1 ========
SFO1 500.1930889 MHz
NUC1 1H
P1 10.00 usec
PLW1 18.75000000 W

F2 - Processing parameters
SI 65536
SF 500.1900124 MHz
WDW EM
SSB 0
LB 0.30 Hz
GB 0
PC 1.00

6c

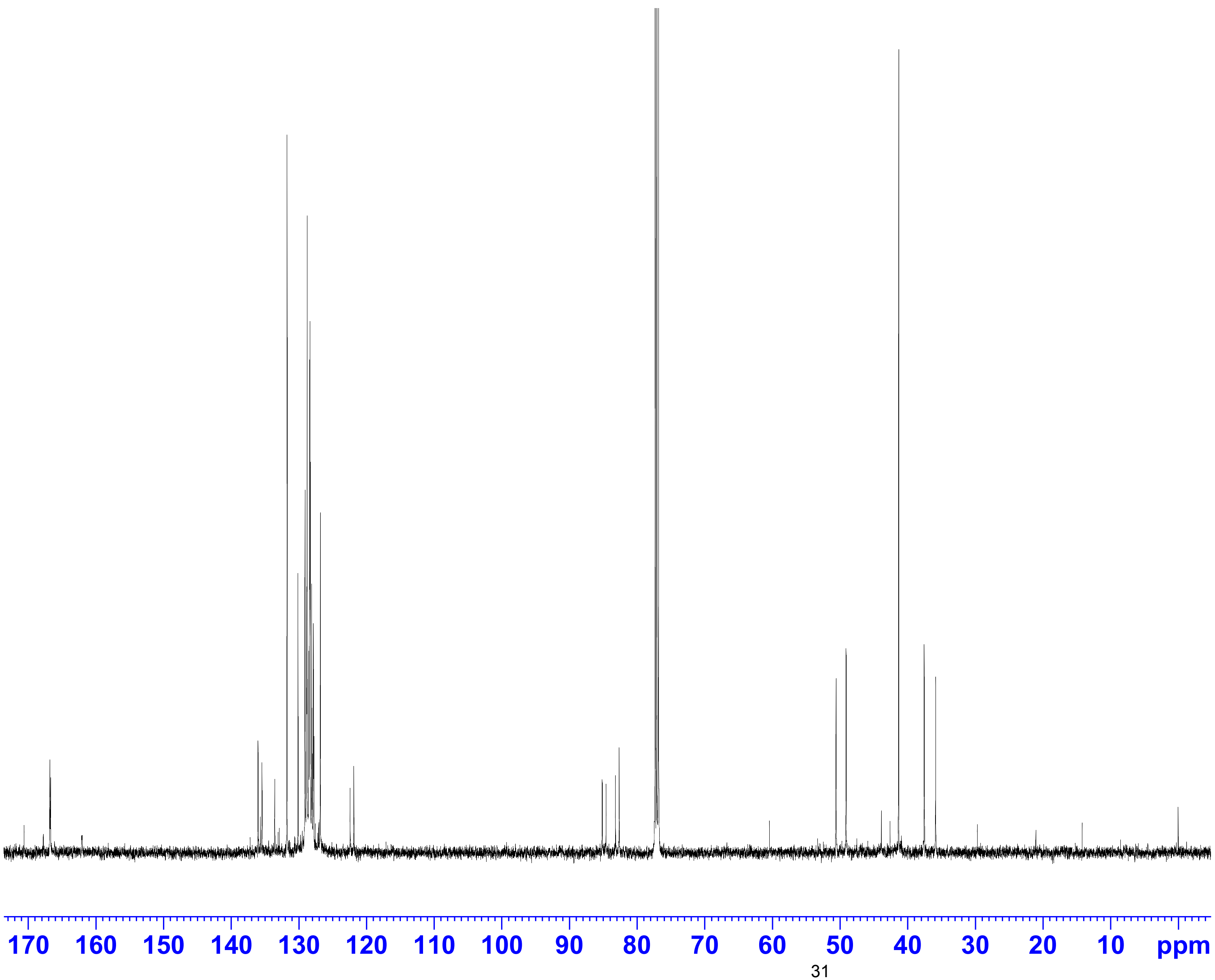

BRUKER

```
Current Data Parameters
NAME        Jul12-2019-SB
EXPNO                  31
PROCNO                  1

F2 - Acquisition Parameters
Date_            20190712
Time                10.56
INSTRUM             spect
PROBHD    5 mm PABBO BB/
PULPROG           zgpg30
SOLVENT            CDCl3
NS                   768
DS                     4
SWH            29761.904 Hz
FIDRES          0.454131 Hz
AQ             1.1010048 sec
RG                191.93
DW                16.800 usec
DE                  6.50 usec
TE                 298.0 K
TD                 65536
D1            2.00000000 sec
D11           0.03000000 sec
TD0                    1

======== CHANNEL f1 ========
SFO1         125.7854522 MHz
NUC1                 13C
P1                  9.65 usec
PLW1         78.00000000 W

======== CHANNEL f2 ========
SFO2         500.1920008 MHz
NUC2                  1H
CPDPRG[2          waltz16
PCPD2              80.00 usec
PLW2         18.75000000 W
PLW12         0.29297000 W
PLW13         0.18750000 W

F2 - Processing parameters
SI                 32768
SF           125.7728810 MHz
WDW                   EM
SSB                    0
LB                  1.00 Hz
GB                     0
PC                  1.40
```

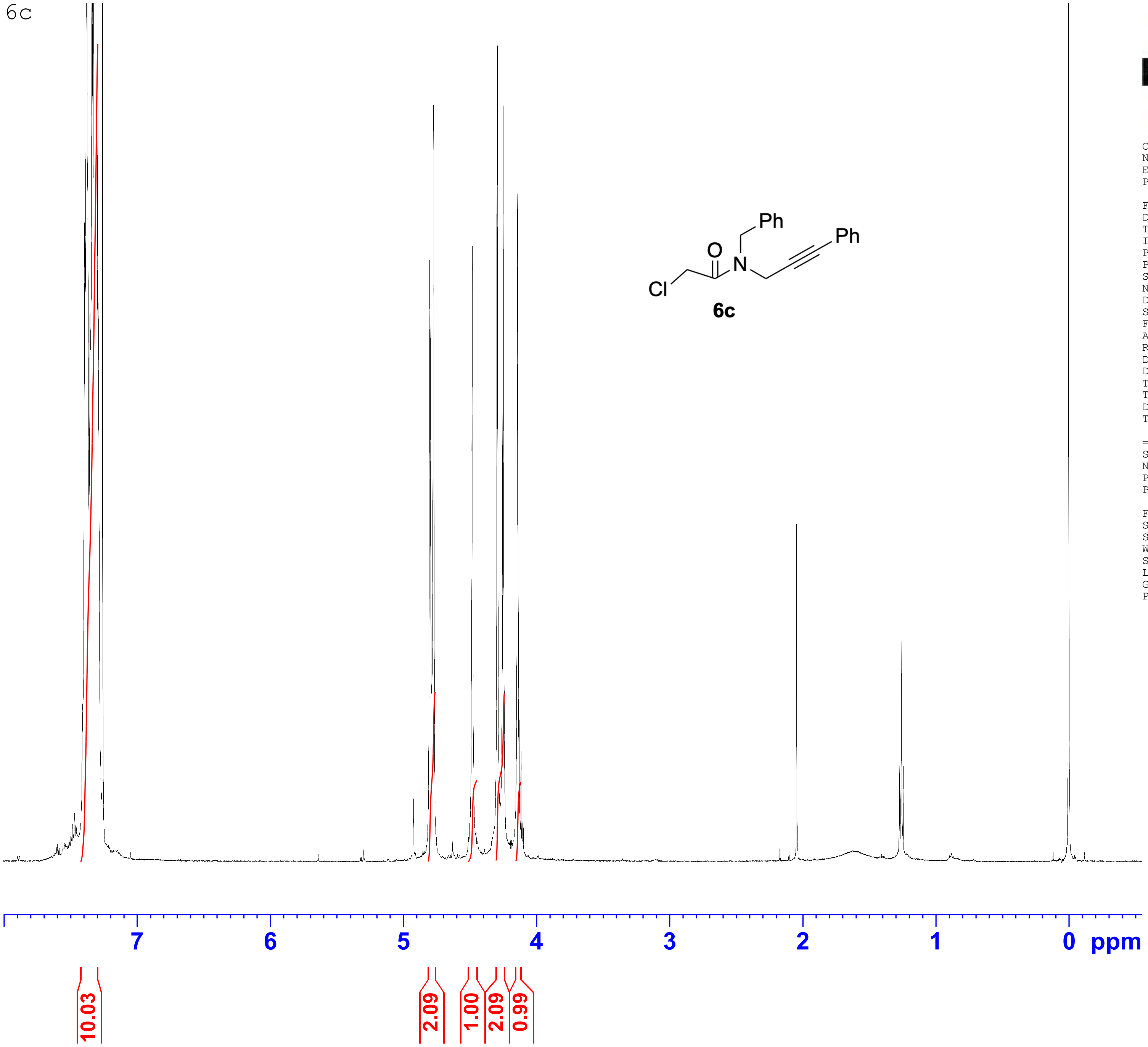

Current Data Parameters
NAME Aug22-2020-SB
EXPNO 10
PROCNO 1

F2 - Acquisition Parameters
Date_ 20200822
Time 11.36
INSTRUM spect
PROBHD 5 mm PABBO BB/
PULPROG zg30
SOLVENT CDCl3
NS 16
DS 2
SWH 10000.000 Hz
FIDRES 0.152588 Hz
AQ 3.2767999 sec
RG 137.92
DW 50.000 usec
DE 6.50 usec
TE 298.0 K
TD 65536
D1 1.00000000 sec
TD0 1

======== CHANNEL f1 ========
SFO1 500.1930889 MHz
NUC1 1H
P1 10.00 usec
PLW1 18.75000000 W

F2 - Processing parameters
SI 65536
SF 500.1900125 MHz
WDW EM
SSB 0
LB 0.30 Hz
GB 0
PC 1.00

6d

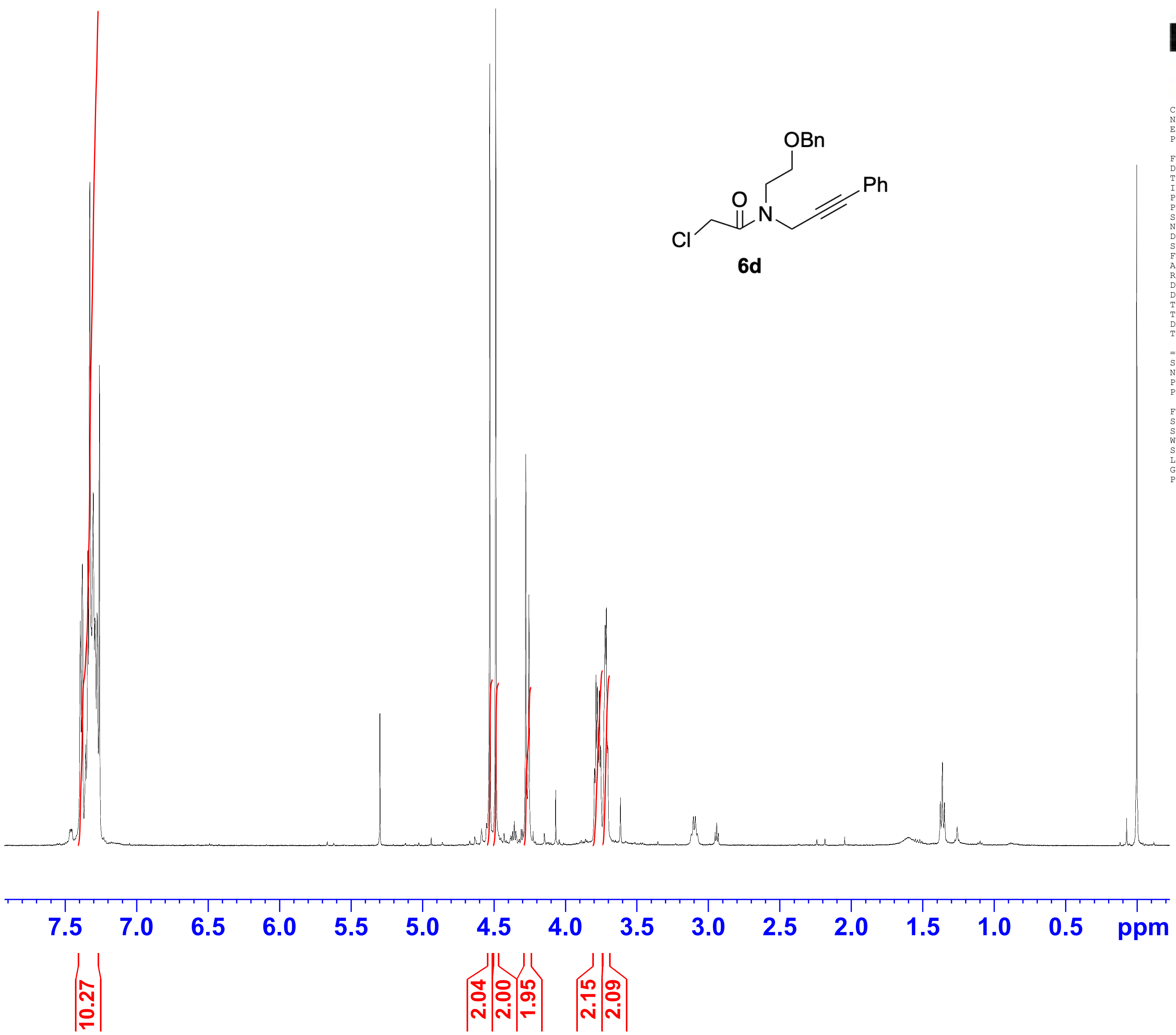

6d

```
Current Data Parameters
NAME       Feb03-2020-SB
EXPNO                 30
PROCNO                 1

F2 - Acquisition Parameters
Date_           20200203
Time               13.04
INSTRUM            spect
PROBHD   5 mm PABBO BB/
PULPROG             zg30
SOLVENT            CDCl3
NS                    16
DS                     2
SWH            10000.000 Hz
FIDRES          0.152588 Hz
AQ             3.2767999 sec
RG                137.92
DW                50.000 usec
DE                  6.50 usec
TE                 298.0 K
TD                 65536
D1            1.00000000 sec
TD0                    1

======== CHANNEL f1 ========
SFO1         500.1930889 MHz
NUC1                  1H
P1                 10.00 usec
PLW1         18.75000000 W

F2 - Processing parameters
SI                 65536
SF           500.1900122 MHz
WDW                   EM
SSB                    0
LB                  0.30 Hz
GB                     0
PC                  1.00
```

HRMS spectra for tested compounds

3a

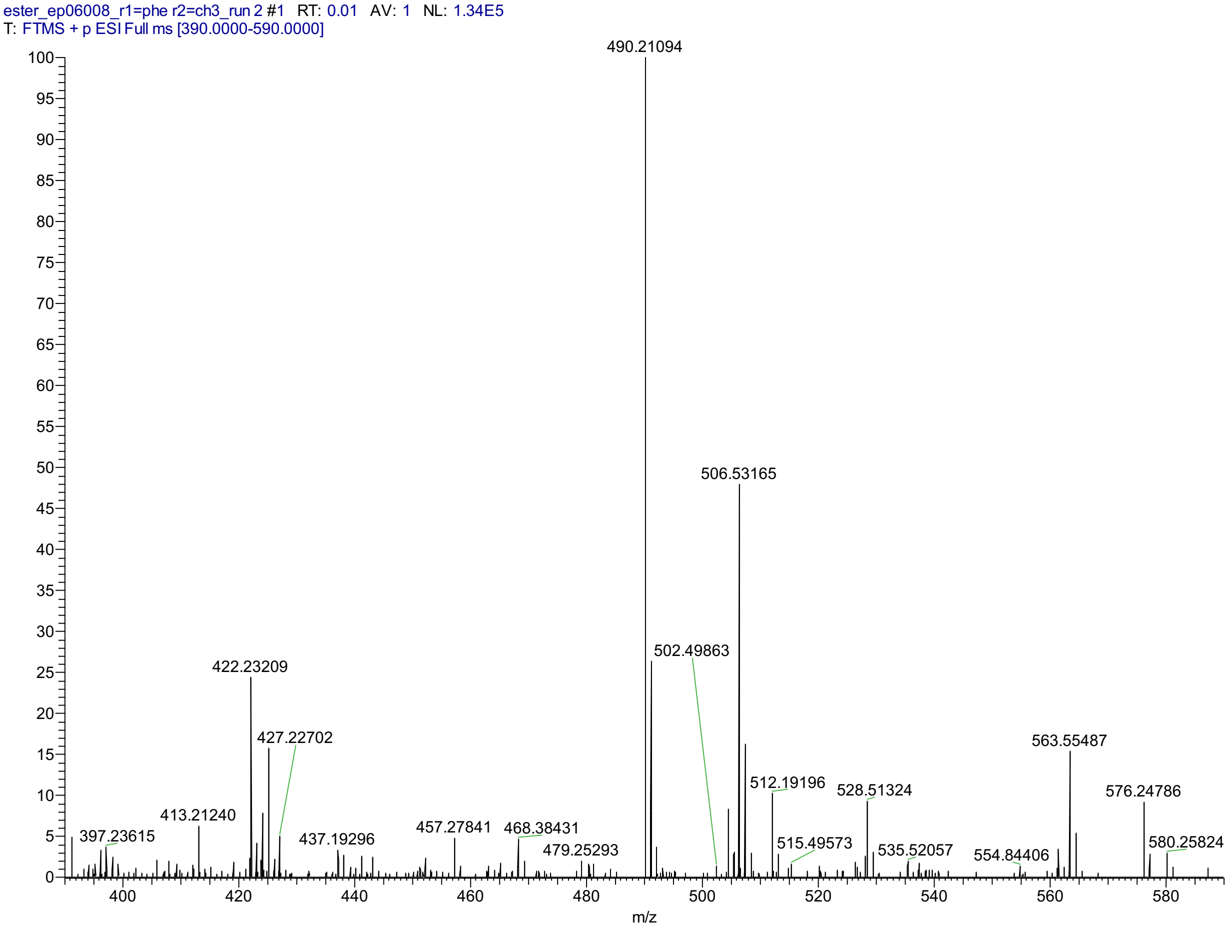
ester_ep06008_r1=phe r2=ch3_run 2 #1 RT: 0.01 AV: 1 NL: 1.34E5
T: FTMS + p ESI Full ms [390.0000-590.0000]
490.21094
506.53165
502.49863
422.23209
427.22702
563.55487
512.19196
528.51324
576.24786
413.21240
397.23615
437.19296
457.27841
468.38431
479.25293
515.49573
535.52057
554.84406
580.25824
m/z

3c

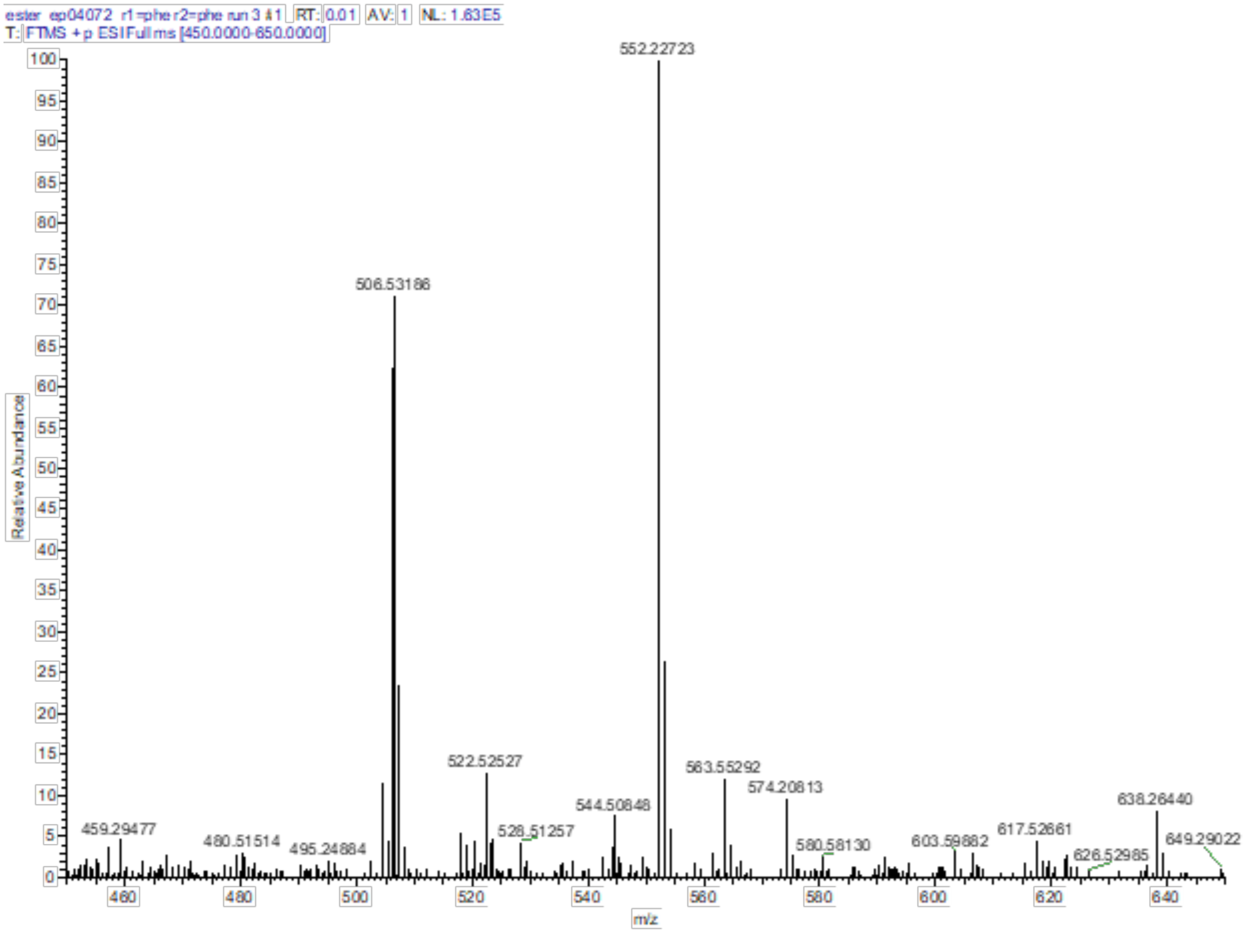
ester ep04072 r1=phe r2=phe run 3 #1 RT: 0.01 AV: 1 NL: 1.63E5
T: FTMS + p ESI Full ms [450.0000-650.0000]
552.22723
506.53186
522.52527
563.55292
574.20813
544.50848
638.26440
459.29477
480.51514
495.24884
528.51257
580.58130
603.59882
617.52661
626.52985
649.29022
Relative Abundance
m/z

3e

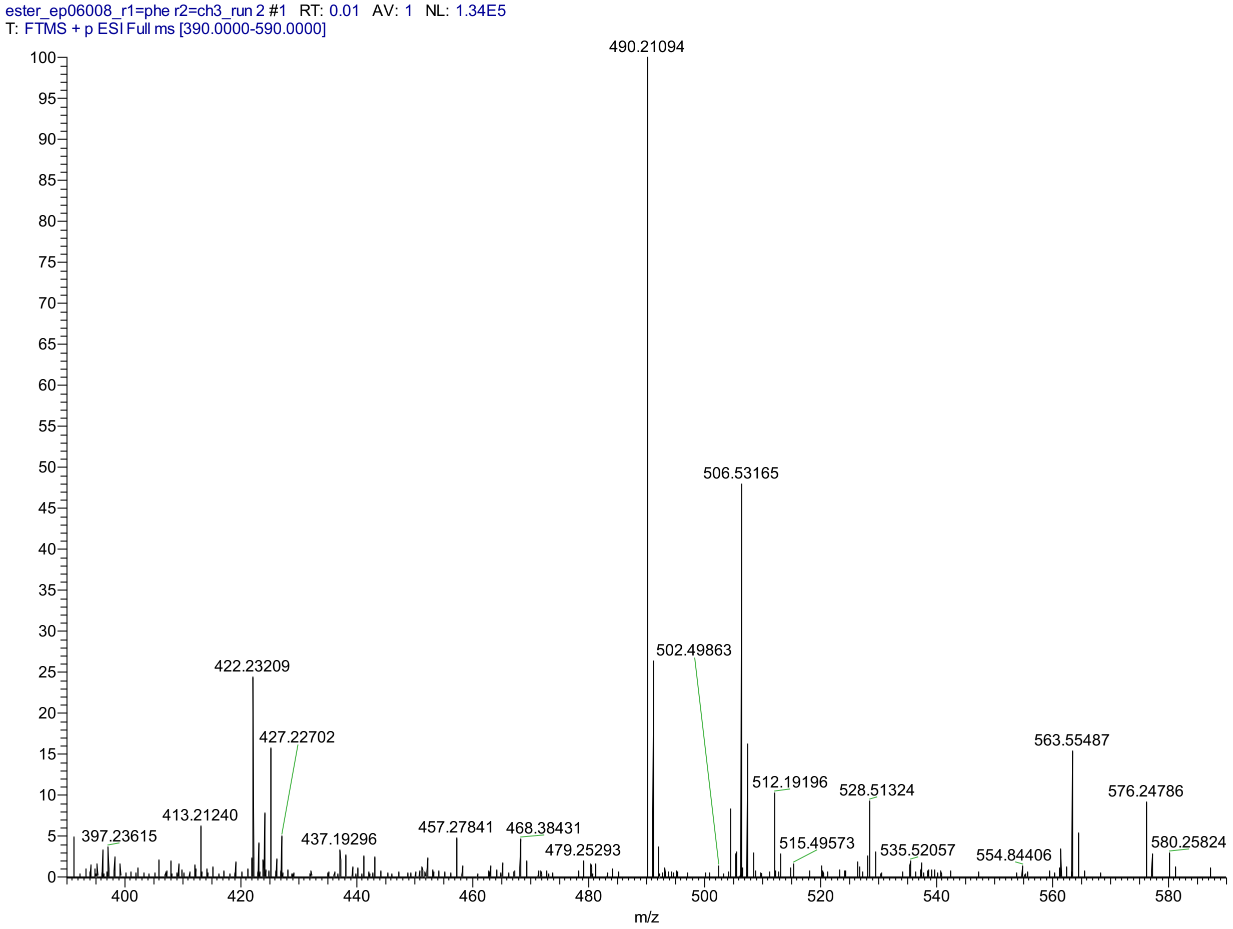

ester_ep06008_r1=phe r2=ch3_run 2 #1 RT: 0.01 AV: 1 NL: 1.34E5
T: FTMS + p ESI Full ms [390.0000-590.0000]
490.21094
506.53165
502.49863
422.23209
427.22702
563.55487
512.19196
528.51324
576.24786
413.21240
397.23615
437.19296
457.27841
468.38431
479.25293
515.49573
535.52057
554.84406
580.25824
m/z

3g

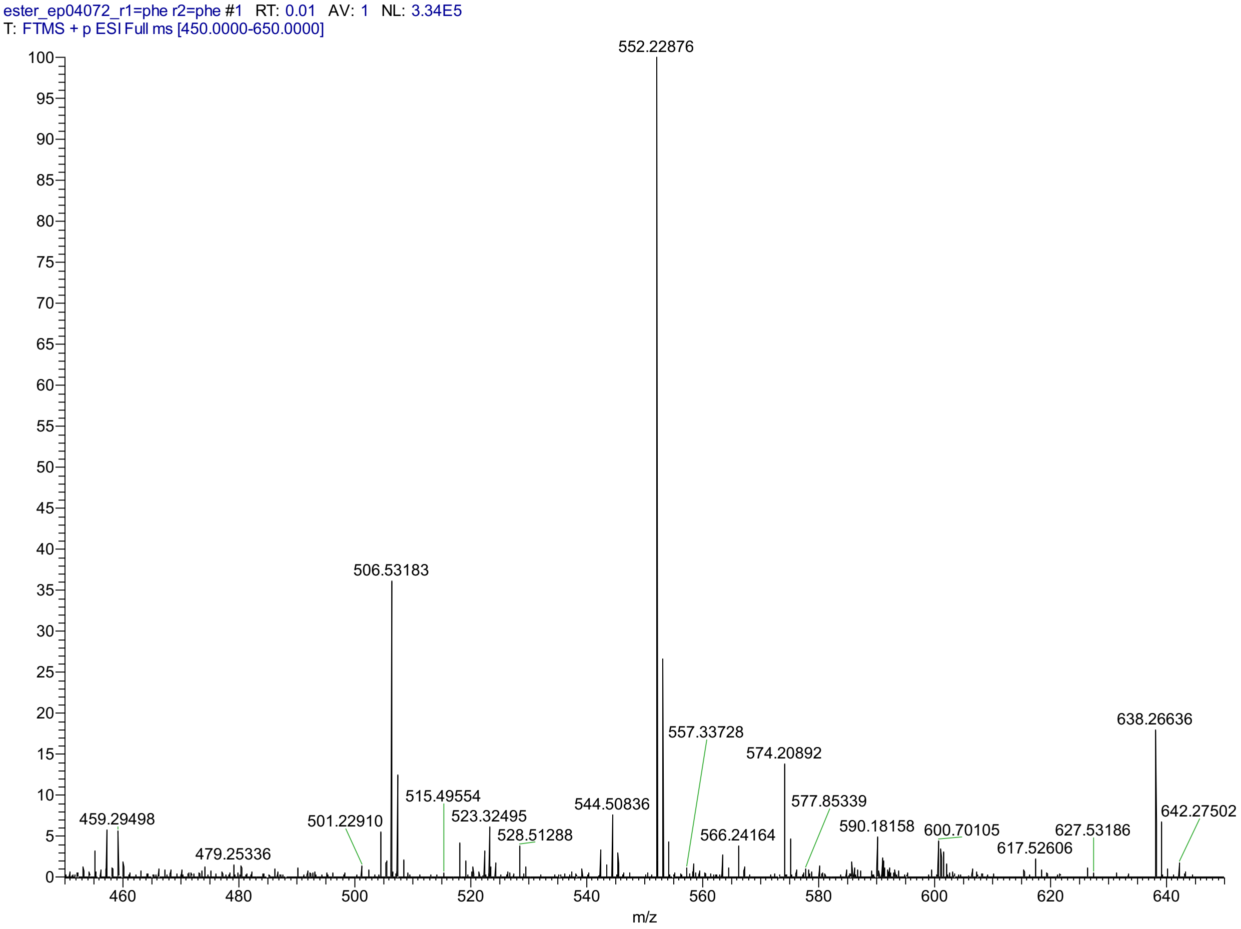

ester_ep04072_r1=phe r2=phe #1 RT: 0.01 AV: 1 NL: 3.34E5
T: FTMS + p ESI Full ms [450.0000-650.0000]
552.22876
506.53183
557.33728
574.20892
638.26636
515.49554
544.50836
577.85339
642.27502
459.29498
501.22910
523.32495
528.51288
566.24164
590.18158
600.70105
627.53186
617.52606
479.25336
m/z

3i

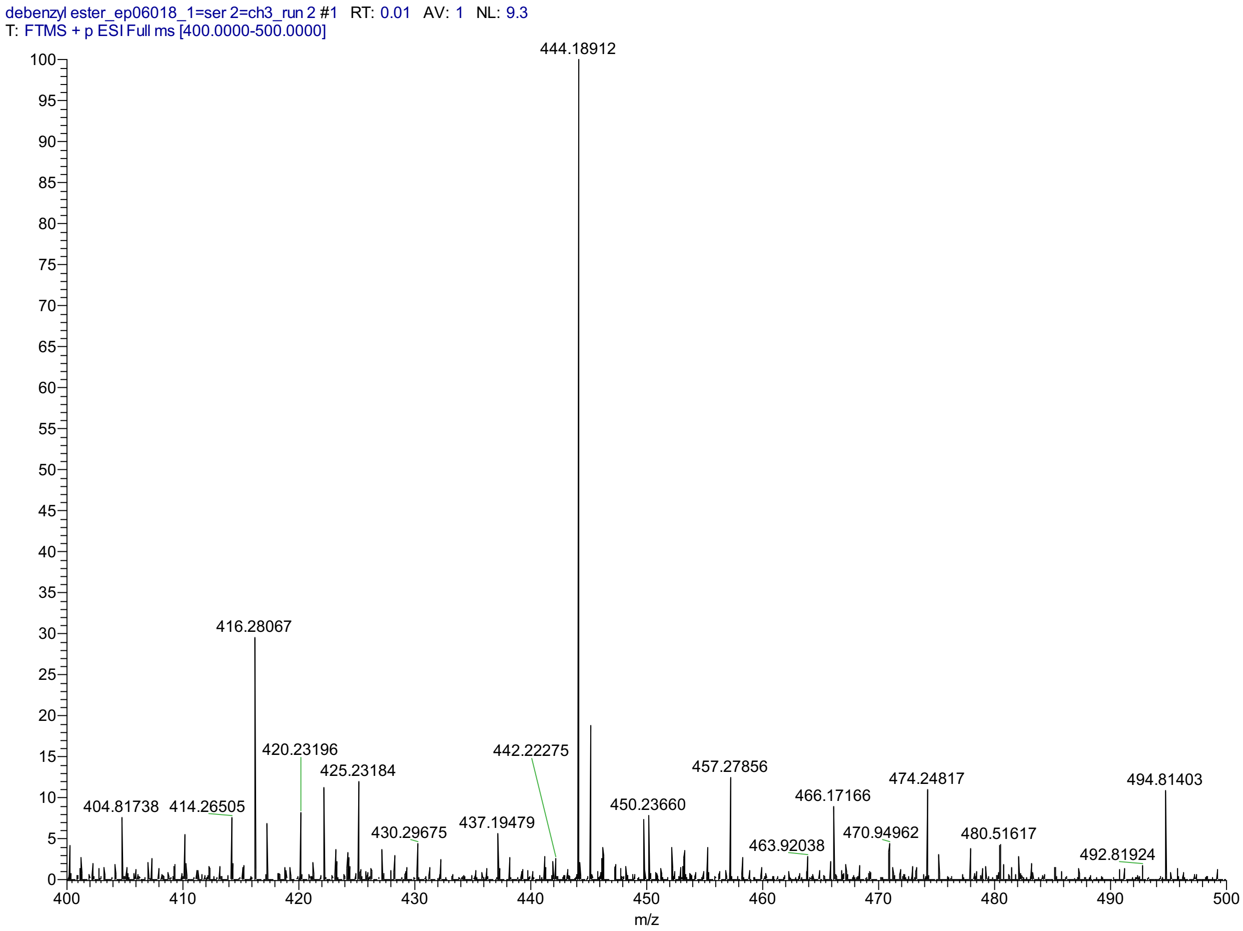
debenzyl ester_ep06018_1=ser 2=ch3_run 2 #1 RT: 0.01 AV: 1 NL: 9.3
T: FTMS + p ESI Full ms [400.0000-500.0000]
444.18912
416.28067
420.23196
442.22275
425.23184
457.27856
474.24817
494.81403
404.81738
414.26505
450.23660
466.17166
430.29675
437.19479
470.94962
480.51617
463.92038
492.81924
m/z

3j

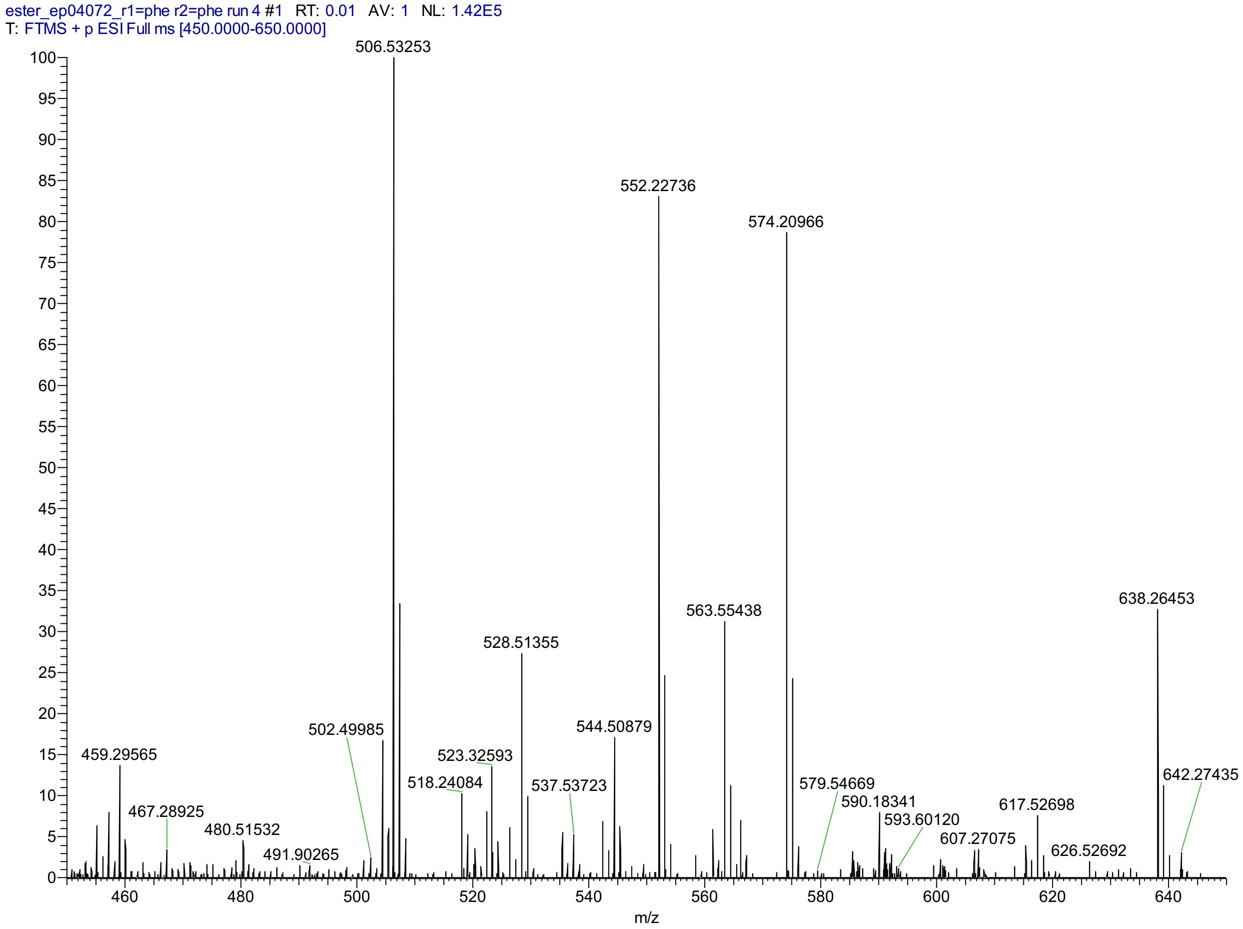

ester_ep04072_r1=phe r2=phe run 4 #1 RT: 0.01 AV: 1 NL: 1.42E5
T: FTMS + p ESI Full ms [450.0000-650.0000]
506.53253
552.22736
574.20966
638.26453
563.55438
528.51355
544.50879
502.49985
459.29565
523.32593
518.24084
537.53723
579.54669
642.27435
590.18341
617.52698
467.28925
593.60120
480.51532
607.27075
626.52692
491.90265
m/z

3k

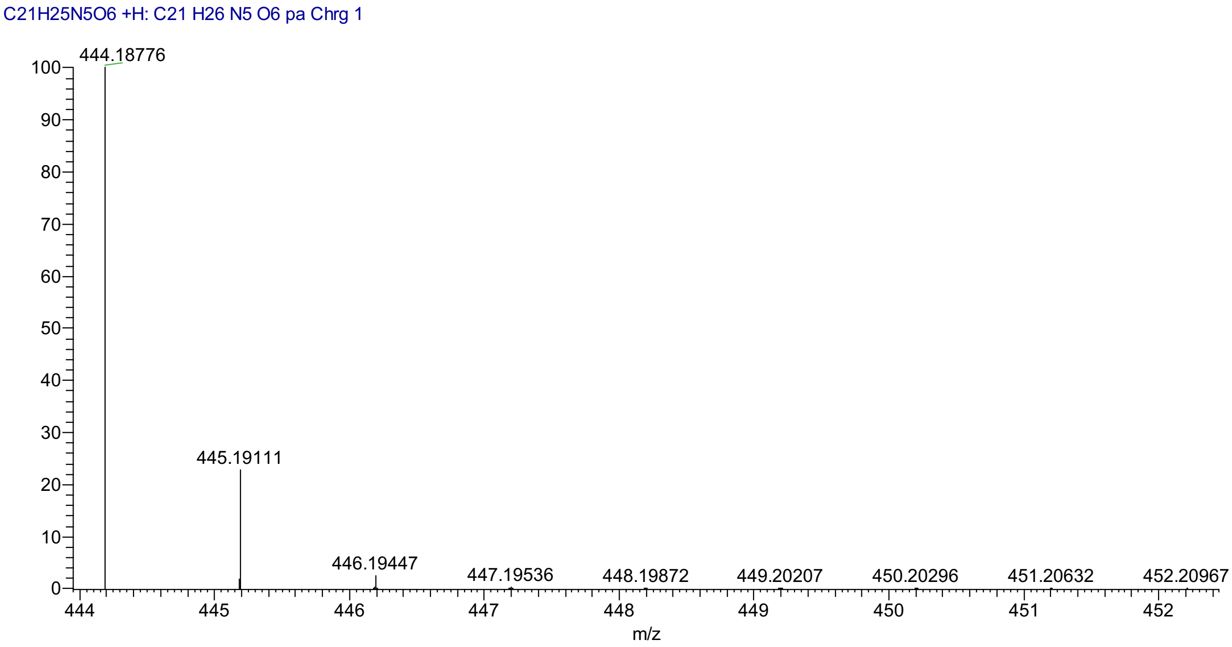
C21H25N5O6 +H: C21 H26 N5 O6 pa Chrg 1
444.18776
445.19111
446.19447
447.19536
448.19872
449.20207
450.20296
451.20632
452.20967
100
90
80
70
60
50
40
30
20
10
0
444
445
446
447
448
449
450
451
452
m/z

3l

C26H27N5O6 +H: C26 H28 N5 O6 pa Chrg 1

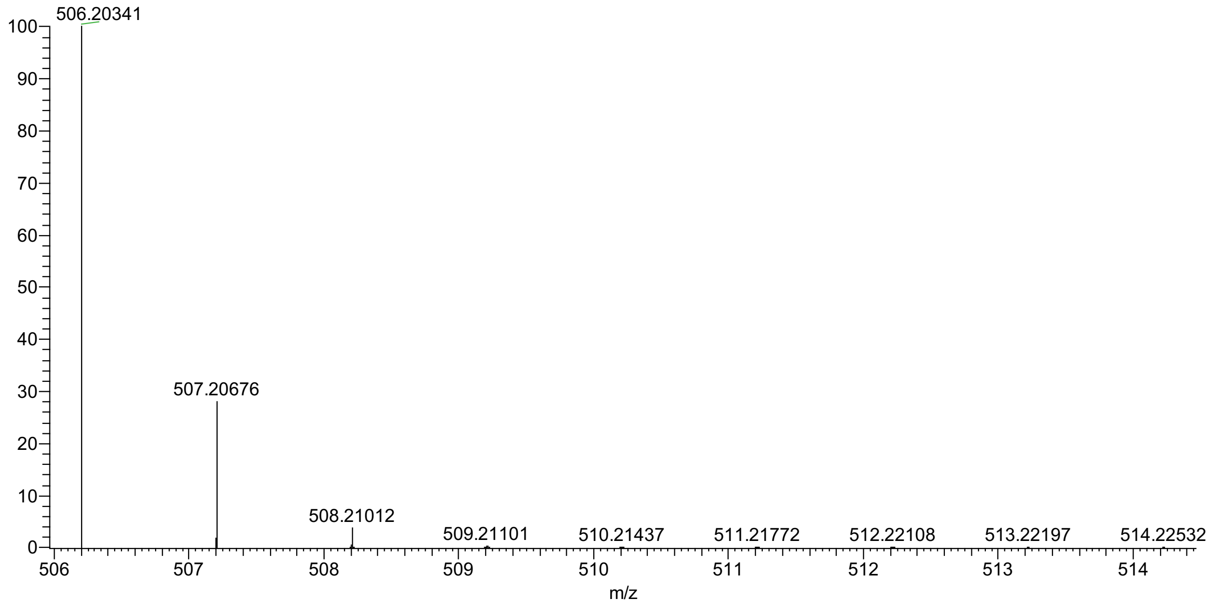

Docking pose of each ligand with lowest Emodel score for docking in the A1 helix

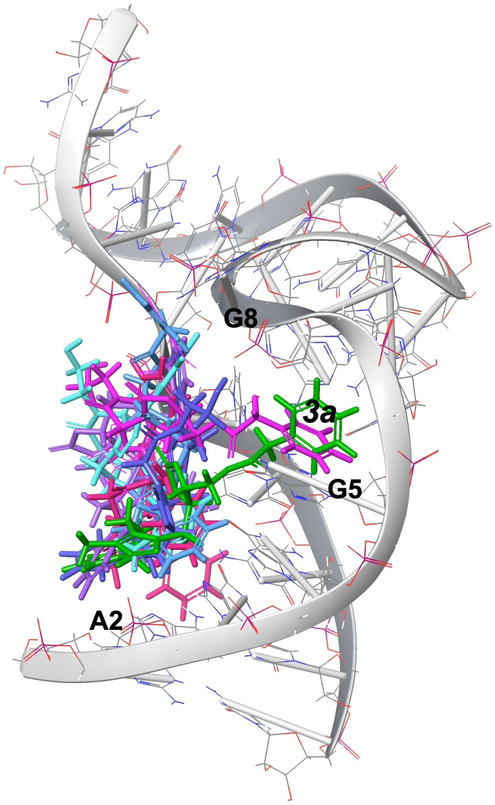

Supplement: Supplementary file 1 [file molecules-31-00029-s001.zip › molecules-3944154-supplementary.pdf]
